# SUMOylation and ubiquitination reciprocally regulate SMCHD1 antiviral activity against herpes simplex virus 1

**DOI:** 10.1371/journal.ppat.1014371

**Published:** 2026-06-24

**Authors:** Xuezhang Tian, Xinyue Wang, Shaowei Wang, Yunhong Zhong, Yanlin Xia, Yang Chen, Ling He, Dongli Pan, Ke Lan, Junjie Zhang

**Affiliations:** 1 State Key Laboratory of Oral & Maxillofacial Reconstruction and Regeneration, Key Laboratory of Oral Biomedicine Ministry of Education, Hubei Key Laboratory of Stomatology, School & Hospital of Stomatology, State Key Laboratory of Virology and Biosafety, Medical Research Institute, Wuhan University, Wuhan, China; 2 Frontier Science Center for Immunology and Metabolism, Medical Research Institute, Wuhan University, Wuhan, China; 3 Hubei Key Laboratory of Tumor Biological Behavior, Hubei Province Cancer Clinical Study Center, Zhongnan Hospital of Wuhan University, Wuhan, China; 4 State Key Laboratory for Diagnosis and Treatment of Infectious Diseases, the First Affiliated Hospital, Zhejiang University School of Medicine, Hangzhou, Zhejiang, China; 5 Department of Medical Microbiology and Parasitology, Zhejiang University School of Medicine, Hangzhou, Zhejiang, China; 6 State Key Laboratory of Virology and Biosafety, School of Life Sciences, Wuhan University, Wuhan, China; Washington State University, UNITED STATES OF AMERICA

## Abstract

Host restriction factors serve as intrinsic barriers against viral infection, and are frequently counteracted by viral antagonists. Previous studies, including our own, have identified SMCHD1 as a restriction factor that suppresses the replication of multiple viruses. Here, we reveal that the antiviral activity of SMCHD1 is dynamically regulated by two different post-translational modifications. SUMOylation of SMCHD1 promotes its association with the viral genome and enhances its antiviral activity. In contrast, during herpes simplex virus 1 (HSV-1) infection, the viral E3 ligase ICP0 induces SMCHD1 ubiquitination and proteasomal degradation, thereby relieving viral restriction. Loss of ICP0 stabilizes SMCHD1 and leads to marked accumulation of SUMOylated SMCHD1, rendering ICP0-deficient HSV-1 more sensitive to SMCHD1-mediated inhibition. Together, our findings uncover a reciprocal SUMO-ubiquitin regulatory mechanism that governs SMCHD1 antiviral activity and highlight a refined virus-host arms race centered on biphasic modification of a single restriction factor.

## Author summary

Herpes simplex virus 1 (HSV-1) establishes lifelong infection in humans and must overcome host antiviral defenses to replicate efficiently. One critical layer of this defense is formed by cellular “restriction factors,” proteins that can directly suppress viral infection. In this study, we investigated how the host restriction factor SMCHD1 inhibits HSV-1 and how the virus counteracts this antiviral activity. We found that a cellular modification called SUMOylation enhances the ability of SMCHD1 to associate with viral genomes and suppress viral gene expression. In response, HSV-1 uses the viral E3 ubiquitin ligase ICP0 to trigger SMCHD1 degradation through ubiquitination, thereby relieving viral restriction and promoting infection. Our findings reveal a dynamic molecular battle in which host cells and viruses use different protein modifications to regulate antiviral defense. This work provides new insight into how herpesviruses evade intrinsic immunity and identifies regulatory pathways that could potentially be exploited for future antiviral strategies.

## Introduction

Host-encoded restriction factors lie at the center of the evolutionary arms race between viruses and their hosts, providing intrinsic barriers that curtail viral replication before inducible immune responses are activated [1–3]. Classic examples in retroviral infection include BST2/Tetherin, which restricts HIV-1 viral particle release and is counteracted by the viral protein Vpu, and SAMHD1, which depletes intracellular dNTP pools to impede reverse transcription and is antagonized by HIV-2/SIV Vpx [4–8]. Extending these insights, accumulating evidence has uncovered a growing repertoire of intrinsic effectors targeting herpesviruses [9], including Mx2/MxB [10–12], TRIM family members [13,14], TMEFF1 [15,16], SP140L [17], LSD1 [18], SFPQ [19], and DR5 [20].

Importantly, many host factors restrict HSV-1 infection through chromatin-based repression of viral genomes and are actively counteracted by the viral E3 ubiquitin ligase ICP0. Daxx and PML cooperate to promote the deposition of histone H3.3 onto the HSV-1 genome, thereby limiting viral genome decompaction and suppressing viral gene expression [21]. HSV-1 ICP0 antagonizes this antiviral defense by inducing PML degradation, leading to the dissociation of Daxx and H3.3 from viral genomes and promoting immediate-early viral gene expression [21]. Similarly, the histone chaperones HIRA and ATRX restrict HSV-1 infection through maintenance of repressive chromatin on viral genomes, whereas ICP0 disrupts HIRA recruitment and induces ATRX degradation to facilitate viral infection [22–25]. Together, these findings highlight chromatin-associated antiviral restriction as a major target of HSV-1 immune evasion strategies.

Among these host factors, SMCHD1 (Structural Maintenance of Chromosomes Hinge Domain 1) has recently emerged as a versatile restriction factor that suppresses the replication of multiple viruses [9]. SMCHD1 was initially characterized for its critical role in X chromosome inactivation, chromatin remodeling, and facioscapulohumeral muscular dystrophy (FSHD) pathogenesis [26]. More recently, a genome-wide CRISPR-Cas9 knockout screen identified SMCHD1 as a pan-herpesvirus restriction factor [27], while subsequent studies demonstrated its ability to restrict adeno-associated virus (AAV) transduction [28]. Moreover, SMCHD1 associates with covalently closed circular DNA (cccDNA) of hepadnaviruses and suppresses viral gene transcription [29]. However, the detailed molecular mechanism by which SMCHD1 restricts viral replication, and whether its antiviral activity is targeted by viral countermeasures, remain unclear.

In this study, we uncover a previously unrecognized regulatory mechanism that controls SMCHD1 antiviral function through reciprocal post-translational modifications. We show that SUMOylation of SMCHD1 markedly enhances its association with viral genomes and potentiates its antiviral capacity. In contrast, during HSV-1 infection, the viral E3 ubiquitin ligase ICP0 triggers rapid ubiquitination of SMCHD1 at K1958 and K1976, leading to its proteasomal degradation and thereby relieving viral restriction. Together, these findings reveal a reciprocal SUMO-ubiquitin regulatory mechanism that governs SMCHD1 antiviral activity and exemplify a distinctive host-virus antagonistic strategy centered on a single restriction factor. These findings deepen our understanding of the molecular arms race between host intrinsic immunity and viral evasion, and position SMCHD1 as a potential pivot for antiviral intervention.

## Results

### SMCHD1 restricts HSV-1 replication

**Fig 1:**
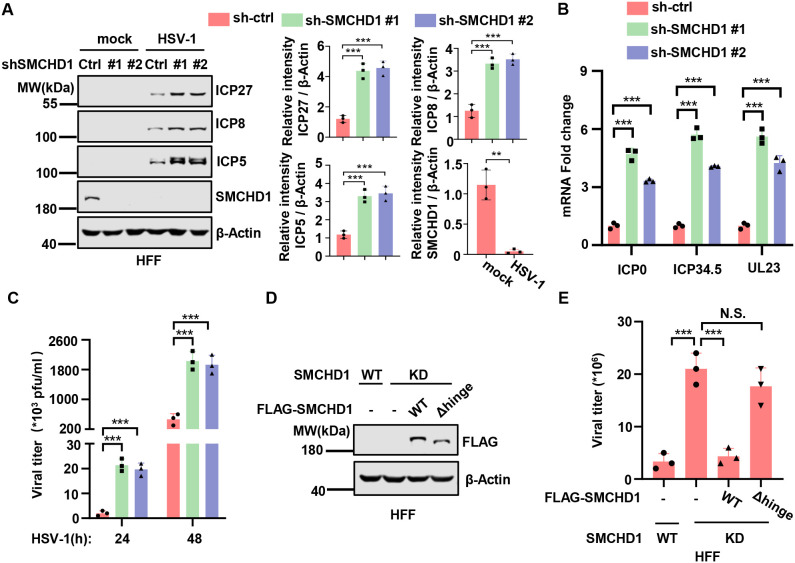
SMCHD1 restricts HSV-1 replication. (A-C) HFF cells transduced with control shRNA or shRNA targeting *SMCHD1* were infected with HSV-1 (MOI = 0.1). Whole-cell lysates (WCLs) were analyzed by immunoblotting at 24 h post-infection, followed by densitometric quantification of band intensities (A). Viral gene expression was quantified by qRT-PCR at 24 h post-infection (B). Viral titers were measured at the indicated time points (C). (D, E) *SMCHD1*-knockdown HFF cells were stably reconstituted with vector control, *SMCHD1* wild-type (WT) or Δhinge mutant through lentiviral transduction. WCLs were analyzed by immunoblotting (D). The reconstituted cells were infected with HSV-1 (MOI = 0.1), and viral titers were determined at 48 h post-infection (E). KD, knockdown. Data are presented as mean ± SD from three independent experiments (n = 3). Statistical significance was determined by one-way ANOVA, unpaired two-tailed Student’s t test, or two-way ANOVA. p value: *, p < 0.05; **, p < 0.01; ***, p < 0.005. N.S.: no significance.

Our previous work identified SMCHD1 as a restriction factor against herpesviruses [27]. To further investigate the role of SMCHD1 in HSV-1 infection, we knocked down *SMCHD1* in HFF and U2OS cells and found that SMCHD1 depletion markedly enhanced viral protein expression by approximately 3–5-fold (Figs 1A and S1A). Notably, HSV-1 infection reduced SMCHD1 protein levels by more than 90% (Figs 1A and S1A), suggesting that HSV-1 may downregulate SMCHD1 to counteract its restrictive activity. Consistently, *SMCHD1* knockdown increased viral gene expression by approximately 6-fold and enhanced progeny virion production by nearly 10-fold (Figs 1B, 1C, S1B, and S1C). Conversely, ectopic expression of SMCHD1 suppressed HSV-1 protein expression, viral gene transcription, and virion production (S1D–S1F Fig). Notably, even stably expressed SMCHD1 was downregulated upon HSV-1 infection (S1D Fig), further confirming that SMCHD1 is actively antagonized during HSV-1 infection.

Our previous study revealed that the DNA-binding hinge domain of SMCHD1 is essential for restricting Kaposi’s sarcoma-associated herpesvirus (KSHV) lytic replication [27]. To determine whether the hinge domain is also required for SMCHD1-mediated restriction of HSV-1, we performed rescue experiments in SMCHD1-depleted cells (Fig 1D). Reintroduction of wild-type (WT) SMCHD1 effectively suppressed HSV-1 replication, whereas a hinge-deletion (Δhinge) mutant failed to do so (Fig 1E), indicating that the hinge domain is critical for the antiviral activity of SMCHD1 against HSV-1. Together, these data indicate that SMCHD1 restricts HSV-1 replication dependent on its hinge domain and is actively counteracted by HSV-1.

### ICP0 targets SMCHD1 for proteasomal degradation

**Fig 2:**
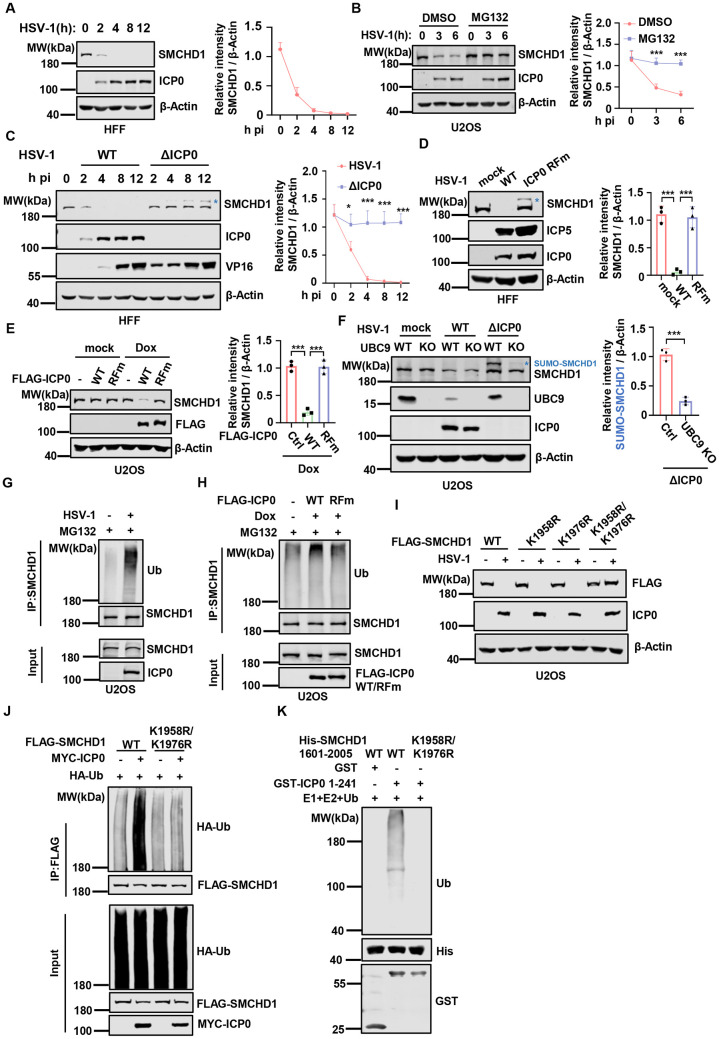
HSV-1 ICP0 targets SMCHD1 for proteasomal degradation. (A) HFF cells were infected with HSV-1 (MOI = 3), and WCLs were analyzed by immunoblotting at the indicated time points post-infection, followed by densitometric quantification of band intensities. (B) U2OS cells were infected with HSV-1 (MOI = 3), and treated with MG132 (10 μM) at 1 h post-infection. WCLs were analyzed by immunoblotting at the indicated time points post-infection, followed by densitometric quantification of band intensities. (C) HFF cells were infected with HSV-1 (MOI = 3) or ΔICP0 (MOI = 5). WCLs were analyzed by immunoblotting at the indicated time points post-infection, followed by densitometric quantification of band intensities. (D) HFF cells were infected with HSV-1 (MOI = 3) or ICP0 RFm (RING finger mutant) (MOI = 5). WCLs were analyzed by immunoblotting at 12 h post-infection, followed by densitometric quantification of band intensities. (E) U2OS cells stably expressing tetracycline-inducible FLAG-ICP0, ICP0 RFm or control vector were treated with doxycycline (Dox, 1 μg/mL) for 24 h. WCLs were analyzed by immunoblotting, followed by densitometric quantification of band intensities. (F) U2OS cells transduced with control sgRNA or sgRNA targeting *UBE2I* were infected with HSV-1 (MOI = 3) or HSV-1 ΔICP0 (MOI = 5) for 24 h. WCLs were analyzed by immunoblotting, followed by densitometric quantification of band intensities. (G) U2OS cells were infected with HSV-1 (MOI = 1) for 6 h, and MG132 (10 μM) was added at 1 h post-infection. Denaturing immunoprecipitation was performed using anti-SMCHD1 antibody, followed by immunoblotting. (H) U2OS stable cells as described in Fig 2E were treated with Dox (1 μg/mL) for 24 h, and MG132 (10 μM) was added at 12 h post-induction. Denaturing immunoprecipitation was performed using anti-SMCHD1 antibody, followed by immunoblotting. (I) U2OS cells stably transduced with SMCHD1 WT or its mutants (K1958R, K1976R, or K1958R/K1976R) were infected with HSV-1 (MOI = 3), and WCLs were analyzed by immunoblotting at 12 h post-infection. (J) HEK293T cells were co-transfected with FLAG-SMCHD1 (WT or K1958R/1976R), MYC-ICP0, and HA-Ub for 24 h, followed by MG132 (10 μM) treatment for 12 h. Denaturing immunoprecipitation was performed using anti-FLAG beads, followed by immunoblotting. (K) In vitro ubiquitination assay was performed using bacterially purified E1 (UBE1), E2 (UBE2D1), ubiquitin, His-SMCHD1 (1601-2005 aa, WT or K1958R/K1976R), and GST or GST-ICP0 (1-241 aa) in the presence of ATP at 37 °C for 1 h, followed by immunoblotting. Data are presented as mean ± SD from three independent experiments (n = 3). Statistical significance was determined by one-way ANOVA, unpaired two-tailed Student’s t test, or two-way ANOVA. p value: *, p < 0.05; **, p < 0.01; ***, p < 0.005. N.S.: no significance.

To investigate how HSV-1 reduces SMCHD1 protein levels, we first confirmed that SMCHD1 protein abundance declined over the course of HSV-1 infection in both HFF and U2OS cells (Figs 2A and S2A). *SMCHD1* mRNA abundance remained unchanged (S2B Fig), suggesting a post-transcriptional regulatory mechanism. Importantly, the proteasome inhibitor MG132 treatment fully restored SMCHD1 abundance (Fig 2B), suggesting that SMCHD1 degradation is proteasome-dependent.

To determine which stage of viral gene expression drives SMCHD1 degradation, we treated HSV-1-infected cells with the viral DNA polymerase inhibitor phosphonoacetic acid (PAA), which blocks viral DNA replication and late gene expression. SMCHD1 degradation still occurred under PAA treatment (S2C Fig), implicating the involvement of immediate-early or early viral gene products in triggering SMCHD1 degradation. Screening HSV-1 mutants lacking immediate-early genes revealed that only deletion of ICP0 abrogated SMCHD1 degradation, whereas all other mutant viruses reduced SMCHD1 levels to an extent similar to wild-type HSV-1 (S2D Fig). Unexpectedly, HSV-1 ΔICP0 infection induced a pronounced upward shift in SMCHD1 electrophoretic mobility, suggesting that SMCHD1 may undergo post-translational modifications during HSV-1 infection (S2D Fig). Consistently, SMCHD1 remained stable throughout the time-course infection with HSV-1 ΔICP0, whereas WT HSV-1 infection led to progressive SMCHD1 degradation (Figs 2C and S2E). Given that ICP0 is a well-established RING-type E3 ubiquitin ligase [30,31], we next examined whether its ligase activity is required for SMCHD1 degradation. Infection with the E3 ligase-deficient ICP0 RFm mutant failed to induce SMCHD1 degradation (Fig 2D), indicating that ICP0 E3 ligase activity is required for SMCHD1 degradation.

To test whether ICP0 expression alone is sufficient to trigger SMCHD1 degradation, we generated tetracycline-inducible ICP0 expression cell lines. Induction of WT ICP0, but not the E3 ligase-deficient RFm mutant, markedly decreased SMCHD1 protein abundance (Fig 2E). As expected, MG132 treatment restored SMCHD1 levels in ICP0-expressing cells, whereas the lysosomal inhibitor bafilomycin A1 (BafA1) had no effect (S2F Fig). These results indicate that ICP0 mediates proteasomal degradation of SMCHD1.

Given that ICP0 can function as a SUMO-targeted E3 ubiquitin ligase [31,32], we next assessed whether SUMOylation contributes to ICP0-mediated SMCHD1 degradation. SMCHD1 degradation proceeded normally in *UBC9*-knockout cells, regardless of whether ICP0 was ectopically expressed or introduced during viral infection (Figs 2F and S2G). Interestingly, the pronounced upward mobility shift of SMCHD1 observed following HSV-1 ΔICP0 infection was abolished in UBC9-knockout cells, suggesting that SMCHD1 undergoes robust SUMOylation during HSV-1 infection (Fig 2F). These results indicate that, although SMCHD1 undergoes strong SUMOylation during HSV-1 infection, SUMOylation is not required for ICP0-driven degradation.

We then examined whether ICP0 induces SMCHD1 ubiquitination. Both HSV-1 infection and inducible ICP0 expression markedly increased SMCHD1 ubiquitination (Fig 2G and 2H). Moreover, only WT ICP0, but not the RFm mutant, promoted SMCHD1 ubiquitination (Fig 2H). To map the ICP0-targeted degron within SMCHD1, we generated a panel of truncation mutants based on its domain structures and nuclear localization signals [26,33] (S2H Fig). All SMCHD1 mutants were degraded during WT HSV-1 infection but remained stable following infection with HSV-1 ΔICP0 (S2I Fig), suggesting that the C-terminal region of SMCHD1 is critical for ICP0-mediated degradation.

To pinpoint the ubiquitination sites by ICP0, we mutated lysine residues within the C-terminal region of SMCHD1 and found that K1958R and K1976R mutations reduced ICP0-mediated ubiquitination (S2J Fig). Consistently, while SMCHD1 WT and the single mutants (K1958R and K1976R) were efficiently degraded during HSV-1 infection, the double mutant (K1958R/K1976R) was fully resistant to degradation (Fig 2I). Furthermore, ICP0 efficiently ubiquitinated WT SMCHD1 but not the double mutant (Fig 2J). To test whether ICP0 directly ubiquitinates SMCHD1, we performed an *in vitro* ubiquitination assay. The ICP0 RING domain (residues 1–241), which is sufficient for E3 ligase activity [30], robustly ubiquitinated a purified C-terminal fragment of SMCHD1 (residues 1601–2005), whereas mutation of K1958 and K1976 abolished ubiquitination (Fig 2K).

Collectively, these data indicate that HSV-1 ICP0 directly ubiquitinates SMCHD1 at lysine residues K1958 and K1976 and targets it for proteasomal degradation.

### ICP0 interacts with SMCHD1

**Fig 3:**
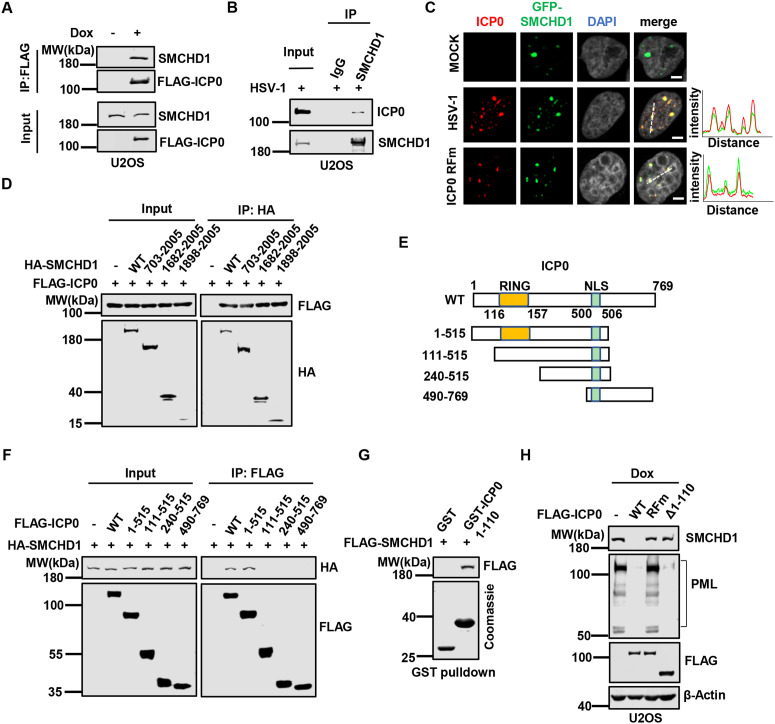
HSV-1 ICP0 interacts with SMCHD1. (A) U2OS cells stably expressing tetracycline-inducible FLAG-ICP0 or vector control were induced with Dox (1 μg/mL) for 12 h, followed by treatment with MG132 (10 μM) for an additional 12 h. Immunoprecipitation was performed using anti-FLAG beads, followed by immunoblotting. (B) U2OS cells were infected with HSV-1 (MOI = 1), and MG132 (10 μM) was added at 1 h post-infection. Immunoprecipitation was performed at 4 h post-infection with anti-SMCHD1 antibody or control IgG, followed by immunoblotting. (C) *SMCHD1*-knockdown U2OS cells were transfected with GFP-SMCHD1 for 24 h and then infected with HSV-1 WT (MOI = 3) or HSV-1 ICP0 RFm (MOI = 3) for 2 h. Immunofluorescence staining was performed using anti-ICP0 antibody, and fluorescence intensity profiles along the indicated lines were shown. Scale bars, 5 μm. (D) HEK293T cells were co-transfected with HA-SMCHD1 or its mutants and FLAG-ICP0. Immunoprecipitation was performed using anti-HA beads, followed by immunoblotting. (E, F) Schematic diagram of ICP0 full-length and truncated mutants (E). HEK293T cells were co-transfected with FLAG-ICP0 or truncated mutants and HA-SMCHD1. Immunoprecipitation was performed with anti-FLAG beads, followed by immunoblotting (F). (G) Bacterially purified GST-ICP0 (1-110 aa) was incubated with FLAG-SMCHD1 purified from mammalian cells for pull-down assays. Input and bound fractions were analyzed by immunoblotting. (H) U2OS cells stably expressing tetracycline-inducible FLAG-ICP0 WT or mutants were induced with Dox (1 μg/mL) for 24 h. WCLs were analyzed by immunoblotting.

Given that ICP0 directly ubiquitinates SMCHD1 and promotes its degradation, we next asked whether ICP0 physically interacts with SMCHD1. Both doxycycline-induced ICP0 and virally expressed ICP0 co-immunoprecipitated with endogenous SMCHD1 (Fig 3A and 3B). Moreover, confocal microscopy revealed that GFP-SMCHD1 formed discrete nuclear foci that strongly colocalized with ICP0 in cells infected with either WT HSV-1 or the ICP0 RFm mutant virus during the early stage of infection (Fig 3C).

Given that ICP0 is known to associate with PML nuclear bodies during HSV-1 infection [34], we further performed immunofluorescence assays to examine the subcellular localization of ICP0, SMCHD1, and PML during the early phase of infection. Our results showed that HSV-1 infection induced prominent colocalization of ICP0, SMCHD1, and PML within discrete nuclear puncta (S3A Fig). In contrast, PML and SMCHD1 did not exhibit detectable colocalization in uninfected cells (S3A Fig).

To define the region of SMCHD1 mediating ICP0 binding, we leveraged our panel of SMCHD1 truncation mutants generated previously (S2H Fig) and found that the C-terminal region of SMCHD1 (residues 1898–2005) was sufficient to mediate the interaction with ICP0, consistent with this region also being required for ICP0-mediated degradation (Figs 3D and S2I).

We next sought to identify the reciprocal ICP0 region responsible for SMCHD1 binding. Guided by previously characterized ICP0 domain architecture [31], we generated a series of ICP0 truncation mutants and found that the N-terminal region of ICP0 (residues 1–110) was essential for SMCHD1 interaction (Fig 3E and 3F). This finding was further supported by *in vitro* binding assays using purified proteins showing that GST-tagged ICP0(1–110) directly interacted with purified SMCHD1 (Fig 3G).

Next, we sought to functionally validate the role of ICP0(1–110) in mediating SMCHD1 degradation. As expected, doxycycline-inducible expression of WT ICP0 efficiently degraded SMCHD1, whereas the RFm mutant did not. Importantly, ICP0(Δ1–110), which fails to bind SMCHD1, also failed to promote its degradation (Fig 3H). As a control, PML, a known ICP0 substrate [32,35], was efficiently degraded by both WT ICP0 and ICP0(Δ1–110) but not by the RFm mutant (Fig 3H), confirming that loss of SMCHD1 degradation by ICP0(Δ1–110) was not due to a general defect in protein turnover.

Previous studies have shown that ICP0 targets RNF8 and RNF168 for ubiquitin-mediated degradation and that CK1-dependent phosphorylation of ICP0 at T67 is required for RNF8 binding [36,37]. To investigate whether RNF8 or RNF168 is involved in ICP0-mediated SMCHD1 degradation, we depleted RNF8 or RNF168 and examined SMCHD1 stability following HSV-1 infection. Notably, loss of either RNF8 or RNF168 did not affect SMCHD1 degradation (S3B Fig). In addition, the ICP0-T67A mutant, which is defective in RNF8 recruitment, still efficiently induced SMCHD1 ubiquitination and degradation (S3C Fig). These findings suggest that ICP0-mediated degradation of SMCHD1 occurs independently of RNF8 and RNF168.

Collectively, these data indicate that ICP0 directly interacts with SMCHD1, and that the N-terminal 1–110 region of ICP0 and the C-terminal 1898–2005 region of SMCHD1 mediate the interaction.

### HSV-1 infection induces SUMOylation of SMCHD1

**Fig 4:**
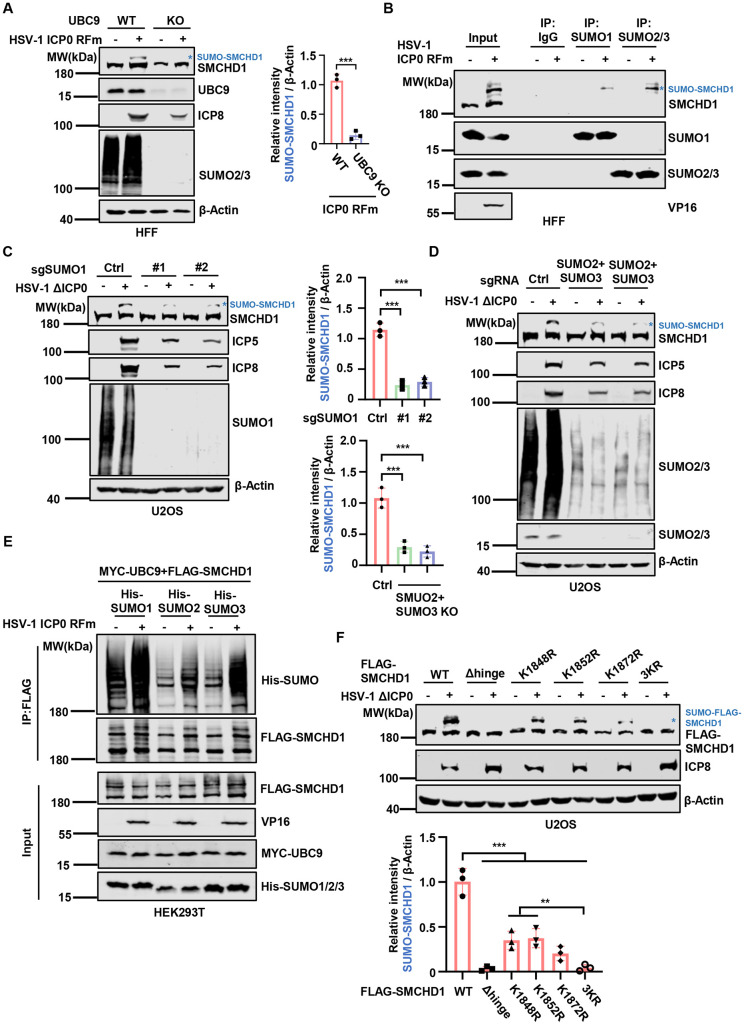
HSV-1 infection induces SUMOylation of SMCHD1. (A) HFF cells stably transduced with control sgRNA or sgRNA targeting *UBE2I* were infected with HSV-1 ICP0 RFm (MOI = 5). WCLs were analyzed by immunoblotting at 24 h post-infection, followed by densitometric quantification of band intensities. (B) HFF cells were infected with HSV-1 ICP0 RFm (MOI = 5) for 24 h. Denaturing immunoprecipitation was performed using anti-SUMO1 or anti-SUMO2/3 antibodies, and both input and immunoprecipitated samples were analyzed by immunoblotting. (C) U2OS cells stably transduced with control sgRNA or sgRNA targeting *SUMO1* were infected with HSV-1 ΔICP0 (MOI = 5) for 24 h. WCLs were analyzed by immunoblotting, followed by densitometric quantification of band intensities. (D) U2OS cells stably transduced with control sgRNA or sgRNA targeting *SUMO2* and *SUMO3* were infected with HSV-1 ΔICP0 (MOI = 5) for 24 h. WCLs were analyzed by immunoblotting, followed by densitometric quantification of band intensities. (E) HEK293T cells were co-transfected with the indicated plasmids for 24 h and subsequently infected with HSV-1 ICP0 RFm (MOI = 5) for 24 h. Denaturing immunoprecipitation was performed, and both input and immunoprecipitated samples were analyzed by immunoblotting. (F) *SMCHD1*-knockdown U2OS cells reconstituted with *SMCHD1* WT, or the indicated mutants (Δhinge, K1848R, K1852R, K1872R, or the 3KR mutant [K1848R/K1852R/K1872R]) were infected with HSV-1 ΔICP0 (MOI = 5) for 24 h. WCLs were analyzed by immunoblotting, followed by densitometric quantification of band intensities. Data are presented as mean ± SD from three independent experiments (n = 3). Statistical significance was determined by one-way ANOVA or unpaired two tailed Student’s t test. p value: *, p < 0.05; **, p < 0.01; ***, p < 0.005. N.S.: no significance.

Our prior data indicated robust SUMOylation of SMCHD1 during HSV-1 infection (Fig 2F). To validate this observation, we generated *UBC9*-knockout HFF and U2OS cells and infected them with HSV-1 ICP0 RFm and ΔICP0, respectively. In both cell types, HSV-1 infection induced a prominent shifted SMCHD1 band that was eliminated upon UBC9 depletion (Figs 4A and S4A). Of note, HSV-1 ΔICP0 was used for infection in U2OS cells because U2OS cells are well recognized to support efficient replication of HSV-1 ΔICP0 [38,39]. In contrast, HSV-1 ICP0 RFm was used for infection in HFF and HEK293T cells because we consistently observed that HSV-1 ΔICP0 exhibited a much more severe replication defect than HSV-1 ICP0 RFm in these restrictive cell types (S4B Fig). ICP0 RFm infection markedly increased the recovery of SMCHD1 in SUMO1 and SUMO2/3 immunoprecipitates (Fig 4B), suggesting that SMCHD1 is modified by both SUMO1 and SUMO2/SUMO3. To further investigate the involvement of SUMO1 and SUMO2/3 in SMCHD1 SUMOylation, we generated *SUMO1*-knockout cells and *SUMO2/3* double-knockout cells. In both cases, SMCHD1 SUMOylation was reduced but not abolished following HSV-1 ΔICP0 infection (Fig 4C and 4D), confirming that SMCHD1 undergoes conjugation by SUMO1 and SUMO2/3 concurrently. Consistently, co-expression of SMCHD1 with SUMO1, SUMO2, or SUMO3 followed by infection with HSV-1 ICP0 RFm significantly increased SUMOylation of SMCHD1 (Fig 4E). Together, these results indicate that HSV-1 infection promotes robust SUMOylation of SMCHD1.

Interestingly, co-expression of SUMO proteins with SMCHD1 induced a detectable electrophoretic mobility shift of SMCHD1 in the absence of HSV-1 infection, suggesting that SMCHD1 may undergo basal SUMO modification under steady-state conditions (Fig 4E). Consistently, in HEK293T cells, SMCHD1 exhibited a weak basal mobility shift that was markedly enhanced upon co-expression of UBC9 and SUMO1–3 (S4C Fig), further supporting basal SUMOylation of SMCHD1.

Given that SMCHD1 hinge domain is required for restricting HSV-1 replication, and that SUMOylation frequently modulates the DNA-binding activity of chromatin-associated proteins [40,41], we hypothesized that the hinge domain may contribute to infection-induced SUMOylation of SMCHD1. Indeed, when reintroduced into SMCHD1-depleted U2OS cells, WT SMCHD1 exhibited robust SUMOylation upon HSV-1 ΔICP0 infection, whereas the Δhinge mutation completely abolished SUMO modification (Fig 4F).

Next, to identify residues potentially involved in infection-induced SUMOylation, we generated hinge domain mutants at lysine residues previously implicated in DNA binding [42], either individually (K1848R, K1852R, and K1872R) or in combination (K1848R/K1852R/K1872R; hereafter referred to as 3KR). When reintroduced into SMCHD1-depleted U2OS cells, single lysine mutants partially reduced HSV-1 ΔICP0-induced SUMOylation, whereas the 3KR mutant almost completely abolished infection-induced SUMO modification (Fig 4F). We further validated these findings by co-expressing WT or mutant SMCHD1 with SUMO1–3 in HEK293T cells followed by HSV-1 ICP0 RFm infection. WT SMCHD1 underwent robust SUMOylation, whereas SUMOylation of the Δhinge and 3KR mutants was barely detectable (S4D–S4F Fig).

Collectively, these data indicate that HSV-1 infection induces SUMOylation of SMCHD1, and suggest that residues K1848, K1852, and K1872 within the hinge domain are linked to infection-induced SUMOylation.

### SUMOylation-associated hinge domain residues contribute to antiviral restriction

**Fig 5:**
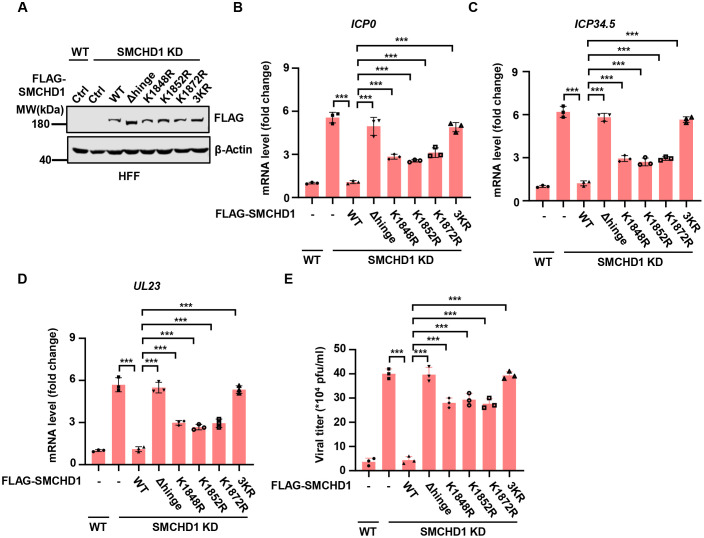
SUMOylation of the SMCHD1 hinge domain confers antiviral restriction. (A-E) *SMCHD1*-knockdown HFF cells were stably reconstituted with vector control, SMCHD1 WT or mutants (Δhinge, K1848R, K1852R, K1872R, and 3KR [K1848R/K1852R/K1872R]) through lentiviral transduction, followed by immunoblotting (A). The stable cells were infected with HSV-1 (MOI = 0.1). Viral gene expression was quantified by qRT-PCR at 24 h post-infection (B-D), and viral titers were quantified at 48 h post-infection (E). Data are presented as mean ± SD from three independent experiments (n = 3). Statistical significance was determined by one-way ANOVA. p value: *, p < 0.05; **, p < 0.01; ***, p < 0.005. N.S.: no significance.

To determine whether SUMO modification contributes to the antiviral function of SMCHD1, we reconstituted SMCHD1 expression in HFF and U2OS cells depleted of endogenous SMCHD1 using either WT or the mutants, followed by HSV-1 infection (Figs 5A and S5A). As expected, reintroduction of WT SMCHD1 significantly suppressed viral gene transcription and replication (Figs 5B-5E and S5B–S5E). In contrast, the hinge domain deletion Δhinge mutant failed to restrict viral replication (Figs 5B-5E and S5B–S5E). Single-lysine substitutions (K1848R, K1852R, or K1872R) each partially impaired SMCHD1-mediated restriction, whereas the triple mutant (3KR), which almost completely abolished infection-induced SUMOylation, largely lost antiviral activity and phenocopied the hinge domain deletion (Figs 5B-5E and S5B–S5E). These data support that infection-induced SUMOylation of the SMCHD1 hinge domain contributes critically to its antiviral activity against HSV-1.

### SMCHD1 associates with HSV-1 genome to repress viral gene transcription

**Fig 6:**
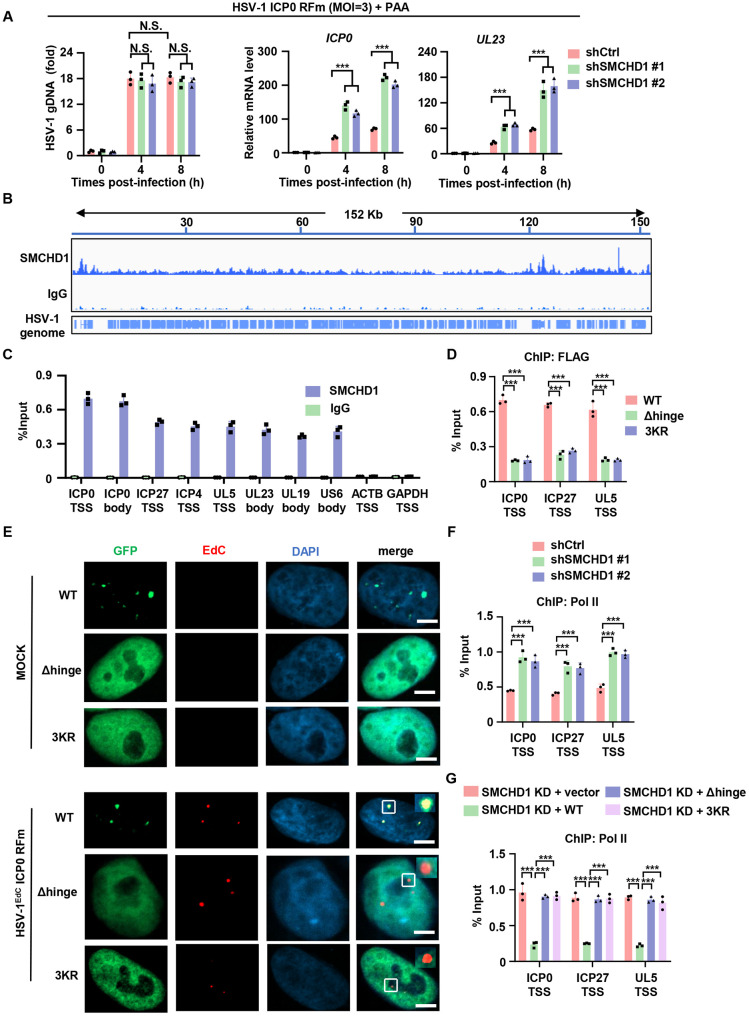
SMCHD1 associates with HSV-1 genome to repress viral gene transcription. (A) Control or *SMCHD1*-knockdown U2OS cells were infected with HSV-1 ICP0 RFm (MOI = 3) in the presence of phosphonoacetic acid (PAA, 200 μg/mL), and viral genomic DNA and viral gene transcripts were quantified at the indicated time points. (B, C) U2OS cells were infected with HSV-1 ICP0 RFm (MOI = 5) for 4 h, followed by CUT&Tag profiling (B) or ChIP assay (C) using anti-SMCHD1 antibody or control IgG. TSS, transcription start site; Body, gene coding sequence. (D) *SMCHD1*-knockdown U2OS cells stably reconstituted with *SMCHD1* WT or mutants (Δhinge and 3KR [K1848R/K1852R/K1872R]) were infected with HSV-1 ICP0 RFm (MOI = 5). ChIP assay with anti-FLAG antibody was performed at 4 h post-infection. (E) *SMCHD1*-knockdown U2OS cells transfected with *SMCHD1* WT or mutants (Δhinge and 3KR) were infected with EdC-labeled HSV-1 ICP0 RFm (MOI = 3) for 1 h at 4°C, followed by an additional 2 h incubation at 37°C in the presence of cycloheximide (100 μg/mL). GFP-tagged SMCHD1 or its mutants were visualized by immunofluorescence, nuclei were counterstained with DAPI, and viral genome was detected by click chemistry. Scale bars, 5 μm. (F) ChIP assay with anti-RNA polymerase II (Pol II) antibody was performed in control or SMCHD1-depleted U2OS cells infected with HSV-1 ICP0 RFm (MOI = 5) at 4 h post-infection. (G) *SMCHD1*-knockdown U2OS cells stably reconstituted with vector control, SMCHD1 WT or mutants (Δhinge and 3KR) were infected with HSV-1 ICP0 RFm (MOI = 5) for 4 h, followed by ChIP assays with antibodies against Pol II. Data are presented as mean ± SD from three independent experiments (n = 3). Statistical significance was determined by two-way ANOVA. p value: *, p < 0.05; **, p < 0.01; ***, p < 0.005. N.S.: no significance.

To investigate how SMCHD1 restricts HSV-1 replication, we examined viral gene expression in WT and SMCHD1-depleted cells in the presence of the viral DNA polymerase inhibitor PAA. PAA treatment completely blocks viral DNA replication, allowing assessment of transcription exclusively from incoming viral genomes (Fig 6A). Under PAA treatment, transcripts of the immediate-early gene ICP0 and the early gene UL23 were significantly elevated in *SMCHD1*-knockdown cells compared with control cells (Fig 6A). Consistently, increased expression of immediate-early and early viral proteins was also observed in PAA-treated SMCHD1-depleted cells, whereas the late gene product ICP5, which requires viral DNA replication, remained undetectable (S6A Fig). These results indicate that SMCHD1 suppresses HSV-1 transcription at an early stage of infection, prior to viral DNA replication.

Given our earlier observation that the SMCHD1 Δhinge mutant, which lacks DNA-binding activity, was unable to restrict HSV-1 replication (Fig 1E), we hypothesized that SMCHD1 represses HSV-1 transcription by binding to the viral genome. To test this hypothesis, we performed CUT&Tag sequencing and found that SMCHD1 broadly associated across the viral genome without evident sequence specificity (Fig 6B). We then validated SMCHD1 occupancy on HSV-1 genome by ChIP in both U2OS and HFF cells (Figs 6C and S6B).

To investigate whether SUMOylation contributes to SMCHD1 binding to the viral genome, we performed ChIP assays in WT and UBC9-knockout cells upon HSV-1 ICP0-RFm infection. Loss of UBC9 markedly reduced SMCHD1 occupancy on the viral genome (S6C Fig), supporting a role for SUMOylation in promoting SMCHD1 viral genome association. To further determine whether SUMOylation-associated hinge domain contributes to viral genome binding, we reintroduced WT, Δhinge, or 3KR SMCHD1 into SMCHD1-depleted U2OS and HFF cells and performed ChIP assays. Neither Δhinge nor 3KR showed detectable binding to HSV-1 genome, in contrast to the robust binding of WT SMCHD1 (Figs 6D and S6D). To further evaluate the contribution of K1848, K1852, and K1872 to SMCHD1 association with the HSV-1 genome, we generated individual lysine-to-arginine mutants and examined their subcellular localization and viral genome binding. Immunofluorescence analysis showed that the K1848R and K1852R mutants displayed subcellular localization patterns comparable to WT SMCHD1, whereas the K1872R mutant exhibited markedly altered localization (S6E Fig). Notably, all three single mutants showed reduced binding to viral DNA compared with WT SMCHD1 (S6F Fig). In addition, our previous results indicate that K1848R, K1852R and K1872R each partially reduced HSV-1 infection-induced SMCHD1 SUMOylation (Fig 4F). Together, these findings suggest that K1848, K1852, and K1872 contribute to infection-induced SUMOylation-associated viral DNA binding by SMCHD1.

Next, we assessed the association of SMCHD1 with viral genomes using immunofluorescence combined with click chemistry to visualize EdC-labeled incoming vDNA in infected cells in the presence of cycloheximide [43]. Notably, GFP-SMCHD1 formed puncta that colocalized with incoming HSV-1 DNA at the early stage of infection (Fig 6E). In contrast, both the Δhinge and 3KR mutants failed to form discrete puncta associated with incoming viral genomes (Fig 6E). SMCHD1 also colocalized with ICP4-positive foci, which mark sites of viral gene expression from incoming viral genomes (S6G Fig). Moreover, SMCHD1 was recruited to viral replication compartments marked by the viral single-stranded DNA-binding protein ICP8 (S6H Fig). Together, these results indicate that SMCHD1 is recruited to incoming viral genomes during the early stage of infection in a manner dependent on the SUMOylation-associated hinge domain.

Since SMCHD1 broadly associates with HSV-1 genome and represses viral gene transcription, and since HSV-1 gene expression depends on host RNA polymerase II (Pol II), we next assessed whether SMCHD1 influences Pol II occupancy. ChIP assays revealed increased Pol II occupancy at HSV-1 promoters in SMCHD1-depleted cells (Fig 6F). Previous studies have established that HSV-1 genomes acquire histones upon nuclear entry [44] and undergo histone modifications, including H3K9me3, H3K27me3, and H3K4me3, which modulates Pol II–mediated transcription [45–48]. Given that SMCHD1 functions as an epigenetic repressor [26], we hypothesized that SMCHD1 promotes heterochromatinization of the viral genome. Supporting this, SMCHD1 depletion reduced the enrichment of heterochromatin marks H3K9me3 and H3K27me3 at HSV-1 promoters, while increasing the enrichment of the euchromatin mark H3K4me3 (S6I and S6J Fig).

To further assess the contribution of SUMOylation-associated hinge domain to transcriptional repression, WT, Δhinge, and 3KR SMCHD1 were reintroduced in SMCHD1-depleted cells. WT SMCHD1 reduced Pol II recruitment to viral promoters, whereas Δhinge and 3KR mutants failed to do so (Fig 6G). Consistently, WT SMCHD1 enhanced deposition of heterochromatin marks and reduced euchromatin marks at viral promoters, whereas Δhinge and 3KR mutants had little effect (S6K and S6L Fig).

Together, these data indicate that SMCHD1 engages HSV-1 genome through its SUMOylation-associated hinge domain, promotes heterochromatinization of viral chromatin, and restricts viral gene transcription.

### ICP0 counteracts SMCHD1-mediated restriction of HSV-1 replication

**Fig 7:**
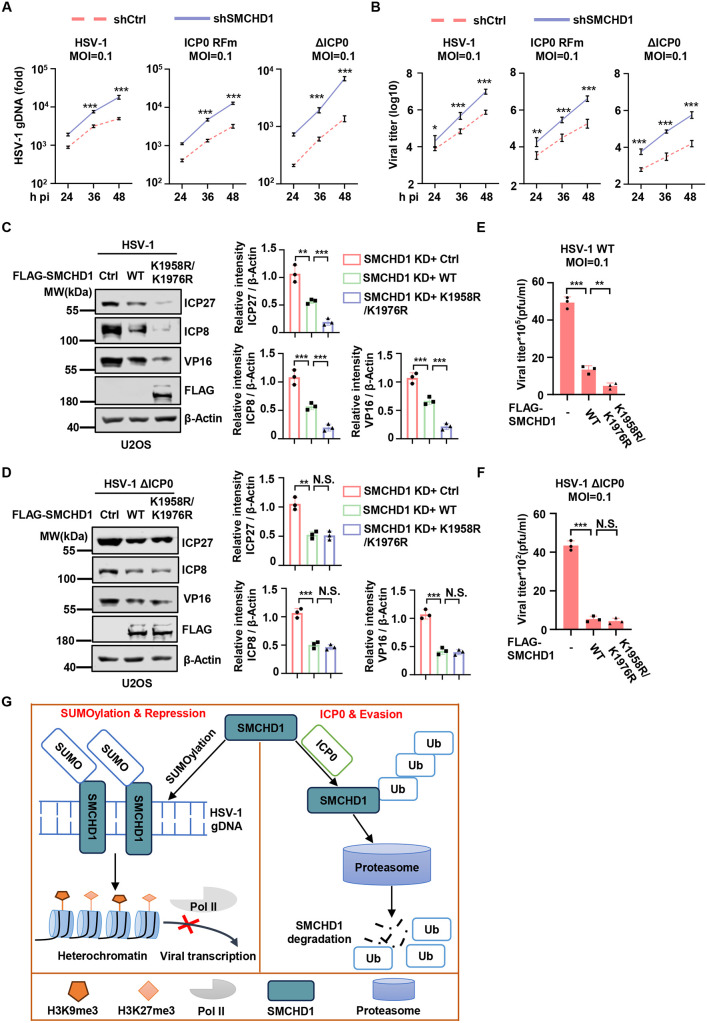
ICP0 counteracts SMCHD1-mediated restriction of HSV-1 replication. (A, B) HFF cells transduced with control shRNA or shRNA targeting *SMCHD1* were infected with HSV-1 (MOI = 0.1), HSV-1 ICP0 RFm (MOI = 0.1), or HSV-1 ΔICP0 (MOI = 0.1). Viral genome copy numbers were quantified by qPCR at the indicated time points (A). Viral titers were quantified for the indicated times post-infection (B). (C-F) *SMCHD1*-knockdown U2OS cells were stably reconstituted with vector control, *SMCHD1* WT, or the K1958R/K1976R mutant. The stable cells were infected with HSV-1 (MOI = 0.1) or HSV-1 ΔICP0 (MOI = 0.1). WCLs were analyzed by immunoblotting at 24 h post-infection, followed by densitometric quantification of band intensities (C, D). HSV-1 viral titers were quantified at 48 h post-infection (E, F). (G) Schematic model illustrating that SUMOylation of SMCHD1 promotes restriction of HSV-1 replication, whereas ICP0-mediated ubiquitination and degradation of SMCHD1 relieves this antiviral restriction. Data are presented as mean ± SD from three independent experiments (n = 3). Statistical significance was determined by one-way ANOVA or two-way ANOVA. p value: *, p < 0.05; **, p < 0.01; ***, p < 0.005. N.S.: no significance.

To assess the functional relevance of SMCHD1 degradation during HSV-1 replication, we compared viral genome replication in HFF cells with either wild-type or SMCHD1 depletion following infection with HSV-1 WT, ICP0 RFm, or ΔICP0 viruses (Fig 7A). To minimize potential bias arising from differences in relative PFU-to-genome ratios among viral strains, we quantified viral genome copy numbers and normalized infections across WT, ΔICP0, and ICP0-RFm viruses based on equivalent viral genome loads in the functional comparison experiments (S7A Fig). In SMCHD1-depleted cells, HSV-1 WT genome replication increased by approximately 3-fold, whereas replication of the ICP0 RFm and ΔICP0 viruses increased more substantially, by approximately 4-fold and 5-fold, respectively, relative to control cells (Fig 7A). Consistently, progeny virion production increased by approximately 13-fold for HSV-1 WT, 21-fold for ICP0 RFm, and 36-fold for ΔICP0 in SMCHD1-depleted cells (Fig 7B). Similar results were obtained in U2OS cells (S7B and S7C Fig). Although U2OS cells have been reported to partially complement ICP0 deficiency, under our experimental conditions WT HSV-1 consistently replicated more efficiently than ICP0-null or ICP0-RFm viruses in U2OS cells (S7D Fig). Together, these data demonstrate that ICP0’s E3 ligase activity plays a central role in counteracting SMCHD1-mediated restriction.

We further assessed the effect of ectopic SMCHD1 expression on HSV-1 replication. Stable expression of SMCHD1 modestly reduced HSV-1 WT titers by approximately 3-fold, whereas HSV-1 ΔICP0 titers were reduced much more substantially, by approximately 9-fold (S7E Fig). These results further confirm that ICP0 antagonizes SMCHD1 and that its absence renders HSV-1 substantially more sensitive to SMCHD1-mediated restriction.

To directly evaluate the impact of ICP0-resistant SMCHD1 on HSV-1 replication, we expressed either SMCHD1 WT or the ubiquitination-deficient mutant (K1958R/K1976R) in SMCHD1-depleted cells (Fig 7C-7F). Upon HSV-1 WT infection, the double mutant exhibited markedly stronger antiviral activity than WT SMCHD1, suppressing both viral protein expression and progeny virion production (Fig 7C and 7E). In contrast, following HSV-1 ΔICP0 infection, WT and mutant SMCHD1 showed comparable antiviral activity (Fig 7D and 7F).

Collectively, these results indicate that ICP0 counteracts SMCHD1-mediated restriction by targeting SMCHD1 for ubiquitination and proteasomal degradation.

## Discussion

Intrinsic restriction factors constitute the first line of cellular defense and exemplify the continuous evolutionary arms race between viruses and their hosts [1,3,49]. In this study, we uncover a previously unrecognized layer of regulation for one such restriction factor, SMCHD1 [27–29], in which reciprocal post-translational modifications, SUMOylation and ubiquitination, antagonistically govern its antiviral potency. SUMOylation of SMCHD1 promotes its association with the viral genome and enhances antiviral activity, whereas HSV-1-encoded viral E3 ubiquitin ligase ICP0 ubiquitinates SMCHD1 and triggers its proteasomal degradation, thereby relieving viral restriction (Fig 7G). To our knowledge, this biphasic post-translational regulation of a single restriction factor highlights a distinctive mechanistic example of how intrinsic immunity can be dynamically regulated by both host and viral factors.

The evolutionary significance of restriction factors that are actively targeted for proteasomal degradation during viral infection is well recognized, as viruses frequently evolve dedicated strategies to antagonize potent host defenses [3,9,50]. During herpesvirus infection, the HSV-1-encoded viral E3 ligase ICP0 targets a repertoire of host substrates, including ND10 components such as PML and Sp100, for degradation. ICP0 preferentially induces the degradation of SUMO-conjugated PML and Sp100 to disperse ND10 nuclear bodies, although ICP0 can also degrade substrates independent of their SUMOylation status [32,35,51,52]. Recently, Schlafen 5 (SLFN5) has been identified as a restriction factor for HSV-1 [53]. SLFN5 binds HSV-1 genome and suppresses viral transcription, while ICP0 promotes its ubiquitination and proteasomal degradation independent of SUMO modification [53]. Other host factors involved in epigenetic silencing of HSV-1 genomes, including HIRA, ATRX, and Daxx, are also counteracted by HSV-1 ICP0 through direct or indirect mechanisms. HIRA and Daxx are antagonized via ICP0-mediated degradation of PML [21,25], whereas ATRX is likely counteracted through ICP0-mediated protein destabilization together with reduced mRNA expression mediated by viral host shutoff (Vhs) and HSV-1-encoded microRNAs [22]. Similar to ATRX-, Daxx-, and HIRA-mediated repression of viral transcription, SMCHD1 occupancy on HSV-1 DNA contributes to the establishment or maintenance of a transcriptionally restrictive chromatin environment. Whether SMCHD1 functionally coordinates with these chromatin-associated epigenetic factors during HSV-1 infection remains to be determined.

To further investigate the mechanism underlying SMCHD1 SUMOylation during HSV-1 infection, we performed two independent SUMO proteomic analyses in the presence or absence of HSV-1 ICP0-RFm infection [54]. Although we identified more than 30 SUMOylated lysine residues within SMCHD1, most sites likely represent basal SUMOylation events because they did not exhibit substantial changes following HSV-1 ICP0-RFm infection. Despite extensive efforts, we were unable to confidently detect infection-induced SUMOylation at K1848, K1852, or K1872. Given the highly dynamic and rapidly reversible nature of SUMO conjugation, together with technical limitations in peptide enrichment and mass spectrometry sensitivity, transient infection-induced SUMOylation events may be difficult to capture experimentally.

Importantly, our functional analyses still support a role for these residues in infection-induced SUMOylation-associated viral DNA binding. K1848R and K1852R mutations did not alter SMCHD1 subcellular localization but reduced both HSV-1-induced SUMOylation and viral DNA binding. In contrast, the K1872R mutation markedly altered SMCHD1 localization, making it difficult to distinguish direct effects on SUMOylation-associated viral DNA binding from indirect effects caused by altered subcellular localization. Accordingly, although our data support that K1848, K1852, and K1872 are functionally linked to infection-induced SUMOylation and viral genome association, we cannot currently conclude that these residues represent definitive HSV-1-induced SUMO acceptor sites. Although K1848 and K1852 do not conform to the canonical ΨKxE SUMOylation consensus motif, previous proteomic studies have shown that a substantial proportion of SUMOylation events occur at non-consensus sites [55,56].

Our results further suggest that SUMOylation promotes SMCHD1 recruitment to HSV-1 genomes and facilitates establishment of a transcriptionally repressive chromatin environment. Previous studies have shown that SUMOylation frequently regulates the chromatin-binding activity of epigenetic repressors and DNA-associated proteins [57,58]. Consistently, loss of UBC9 markedly reduced SMCHD1 association with HSV-1 genomes, accompanied by reduced enrichment of heterochromatin-associated marks and increased recruitment of RNA polymerase II to viral promoters. These findings support a model in which SUMOylation enhances the ability of SMCHD1 to engage viral chromatin and suppress viral transcription. Whether SUMOylation directly alters the DNA-binding affinity of SMCHD1 or instead promotes interactions with additional chromatin-associated cofactors remains an important question for future investigation.

Based on these findings, it will be interesting to determine whether SMCHD1 similarly restricts HIV-1 or other viruses through chromatin-associated mechanisms targeting viral nucleic acids. After HIV-1 enters host cells, its RNA genome is reverse transcribed into DNA, raising the possibility that chromatin-based restriction mechanisms may contribute to suppression of viral transcription. Consistent with our findings during HSV-1 infection, SMCHD1 has been reported to form a complex with LRIF1 and HP1 that associates with the AAV genome, maintaining it in a heterochromatic state and repressing viral transcription [28]. Together, these findings suggest that SMCHD1 may function more broadly as a chromatin-associated restriction factor that suppresses viral gene expression.

Beyond antiviral defense, SMCHD1 is best known for roles in X-chromosome inactivation and its implications in facioscapulohumeral muscular dystrophy (FSHD), where loss-of-function or missense mutations disrupt chromatin architecture [26,59]. In this study, we functionally characterized three hinge-domain lysine residues, K1848, K1852, and K1872, that are functionally linked to infection-induced SUMOylation, viral genome association, and antiviral activity. While derepression of the double homeobox protein 4 (DUX4) resulting from SMCHD1 mutations contributes to FSHD pathogenesis [26], our preliminary query of ClinVar and LOVD did not reveal reported germline variants at these positions, suggesting these residues are not common polymorphic sites in current cohorts. Whether rare alterations affecting these residues influence SMCHD1 chromatin-associated functions or disease susceptibility remains an open question.

In summary, our study defines a reciprocal SUMO–ubiquitin regulatory mechanism that dynamically tunes SMCHD1 antiviral activity. SUMOylation enhances SMCHD1-mediated restriction of viral replication, whereas HSV-1 ICP0-mediated ubiquitination triggers SMCHD1 degradation, relieving viral inhibition. This molecular tug-of-war highlights how a single host factor can be regulated during viral infection and provides a framework for understanding post-translational regulation of intrinsic antiviral immunity.

## Materials and methods

### Cell culture

HEK293T, U2OS, primary human foreskin fibroblast (HFF), and VERO cells (ATCC) were maintained in Dulbecco’s modified Eagle’s medium (DMEM; Hyclone) supplemented with 10% fetal bovine serum (FBS; LONSERA, Shanghai, China) and 1% penicillin–streptomycin (Hyclone). All cell lines were routinely tested for mycoplasma contamination and mycoplasma-negative cells were used in the study.

### Viruses

Herpes simplex virus type 1 (HSV-1), ICP22-deficient HSV-1 (ΔICP22), and ICP47&ICP34.5-deficient HSV-1 (ΔICP47&ΔICP34.5) (F strains) were propagated and titrated in VERO cells as previously described [27,60,61]. ICP27-deficient HSV-1 (ΔICP27; F strain) was propagated and titrated in VERO cells stably expressing ICP27. ICP0-deficient HSV-1 (ΔICP0; F strain) was propagated and titrated using U2OS cells [62]. HSV-1 (KOS strain), HSV-1 ICP0 RING finger mutant (RFm; KOS strain; C116G/C156A), and HSV-1 ΔICP0 (KOS strain; 7134) were kindly provided by Dr. David Knipe (Harvard Medical School) [63–65]. HSV-1, HSV-1 ICP0 RFm, and HSV-1 ΔICP0 (KOS strain) were propagated and titrated in U2OS cells. All viral titers were titrated by standard plaque assays [66].

### Antibodies and other reagents

The following antibodies were used for immunoblotting analysis: rabbit anti-SMCHD1 polyclonal antibody (25589–1-AP, Proteintech; 1:500), rabbit anti-FLAG polyclonal antibody (20543–1-AP, Proteintech; 1:3000); rabbit anti-SMCHD1 monoclonal antibody (CY8117, Abways, Shanghai, China; 1:500); mouse anti-ICP0 monoclonal antibody (sc-53070; 1:1000), mouse anti-ICP8 monoclonal antibody (sc-53329; 1:1000), mouse anti-ICP5 monoclonal antibody (sc-56989; 1:1000), mouse anti-ICP27 monoclonal antibody (sc-69806; 1:1000), and mouse anti-VP16 monoclonal antibody (sc-7545; 1:1000) (Santa Cruz); mouse anti-HA monoclonal antibody (GS20004; 1:5000), mouse anti-FLAG monoclonal antibody (GS20002; 1:5000), and mouse anti-β-Actin monoclonal (GS30002; 1:5000) (Mabuns, Wuhan, China); rabbit anti-RNF8 monoclonal antibody (A22524, Abclonal; 1:1000), rabbit anti-RNF168 polyclonal antibody (A3556, Abclonal; 1:1000), rabbit anti-HA monoclonal antibody (AE105, Abclonal; 1:3000), and rabbit anti-PML monoclonal antibody (A27714, Abclonal; 1:1000). Secondary antibodies included IRDye 800CW goat anti-rabbit, IRDye 800CW goat anti-mouse, IRDye 680CW goat anti-rabbit, and IRDye 680CW goat anti-mouse antibodies (LI-COR; 1:20,000).

Chemical reagents included MG132 (HY-13259, MedChemExpress), and bafilomycin A1 (BafA1; S1413, Selleckchem), doxycycline (Sigma-Aldrich) and puromycin, G418, and blasticidin (Invivogen).

### Constructs

Full-length and mutant SMCHD1 constructs were generated as described previously [27]. GFP-SMCHD1 was sub-cloned into pEF-EF1a-GFP-N. Full-length HSV-1 ICP0 and its mutants were sub-cloned into pEF-EF1a-FLAG-N or pLVX-TetOne-Puro. His-SUMO1/2/3 and MYC-UBC9 plasmids were kindly provided by Dr. Hong-Bing Shu (Wuhan University), and HA-Ub plasmid was kindly provided by Dr. Bo Zhong (Wuhan University). GST-ICP0 (1–241) and GST-ICP0 (1–110) were sub-cloned into pGEX-6P-1. All constructs were verified by Sanger sequencing.

### Stable cell line generation

shRNA-mediated knockdown of *SMCHD1* was performed as previously described [27,67]. sgRNAs targeting SUMO1, SUMO2, RNF168, or RNF8 were cloned into Lenti-CRISPR v2 (Addgene), and sgRNAs targeting UBC9 and SUMO3 were cloned into Lenti-CRISPR-blast. The following sequences were used:

sgUBC9, 5’-CCCAGGAGAGGAAAGCATGG-3’;
sgSUMO1 #1, 5’-GAAGTTTATCAGGAACAAAC-3’;
sgSUMO1 #2, 5’-ACCTTCAACTGAGGACTTGG-3’.
sgSUMO2 #1, 5’-ATATTAATTTGAAGGTGGCG-3’;
sgSUMO2 #2, 5’-GCGGGGCAGGATGGTTCTG-3’;
sgSUMO3 #1, 5’-TGACCACATCAACCTGAAGG-3’;
sgSUMO3 #2, 5’-CTTGATCTTGAACTGCACCA-3’;
sgRNF168, 5’-ATCTGCATGGAAATCCTCG-3’;
sgRNF8, 5’-TTCGTCACAGGAGACCGCGC-3’;

Lentiviruses were produced in HEK293T cells as previously described [68,69]. U2OS or HFF cells were transduced with the indicated lentiviruses for 48 h, followed by selection with puromycin (1 μg/mL) or blasticidin (10 μg/mL) for 48 h. For reconstitution experiments, *SMCHD1*-depleted cells were infected with lentiviruses expressing vector control, SMCHD1 WT, or the mutants, and selected with G418 (500 μg/mL) for 3 days.

### RNA extraction and qRT-PCR

U2OS or HFF cells were infected with HSV-1 or HSV-1 ICP0 RFm at a multiplicity of infection (MOI) of 0.1 or 1 for the indicated times. The infected cells were washed three times with ice-cold PBS, and total RNA was isolated using TRIzol (Takara) according to the manufacturer’s instructions. One microgram of total RNA was reverse-transcribed using TRUEscript RT MasterMix (Aidlab, Beijing, China) according to the manufacturer’s instructions. cDNA was diluted 40-fold and analyzed by quantitative real-time PCR (qRT-PCR) with SYBR Green ChemoHS qPCR Mix (Monad, Wuhan, China). Relative mRNA levels were normalized to the housekeeping gene *ACTB*. Primer sequences are listed in S1 Table.

### CUT&Tag

CUT&Tag was performed using the CUT&Tag 4.0 High-Sensitivity Kit (Novoprotein, Suzhou, China) following the manufacturer’s instructions. In brief, U2OS cells (2 x 10^4^) were infected with HSV-1 ICP0 RFm (MOI = 5) for 4 h, fixed with 1% formaldehyde for 10 min at room temperature, and quenched with glycine. Fixed cells were bound to concanavalin A-coated magnetic beads, and permeabilized with digitonin. After permeabilization, cells were incubated overnight at 4°C with control IgG (2 μg; 30000–0-AP, Proteintech) or anti-SMCHD1 antibody (2 μg; 25589–1-AP, Proteintech), followed by incubation with the secondary antibody (1 μg; AS070, Abclonal) for 2 h at room temperature. A protein A/G-Tn5 (pAG-Tn5) transposome fusion protein was then added to generate chromatin fragments. DNA was purified using Tagment DNA Extract Beads (Novoprotein, Suzhou, China) and amplified to prepare Illumina sequencing libraries.

For data processing, raw data were filtered using Trim Galore (v.0.6.2) and aligned to the human reference genome (hg 38) using Bowtie2 (v.2.5.2) [70] with default parameters. Unmapped clean data were subsequently aligned to HSV-1 KOS genome (GenBank: JQ673480.1) with default parameters. The CUT&Tag peaks were called using SEACR (v1.3) [71]. SMCHD1 CUT&Tag data in wild type U2OS cells mock-infected or infected with HSV-1 ICP0 RFm are available at GEO Database: GSE311667.

### Chromatin immunoprecipitation (ChIP)

ChIP assays were performed as previously described [27,72]. In brief, U2OS or HFF cells were infected with HSV-1 ICP0 RFm (MOI = 5) for 4 h. Approximately 1 x 10^7^ cells were washed three times with ice-cold PBS, and fixed with 1% formaldehyde (Sigma-Aldrich, F8775) for 20 min at room temperature, followed by quenching with glycine. The fixed cells were then lysed with cell lysis buffer (10 mM Tris-Cl [pH 8.0], 10 mM NaCl, 0.5% NP-40, supplemented with a protease inhibitor cocktail). After centrifugation, the nuclear pellets were resuspended in nuclear lysis buffer (50 mM Tris-Cl [pH 8.0], 10 mM EDTA, 1% SDS, supplemented with a protease inhibitor cocktail), and sonicated (Covaris S220) according to the manufacturer’s instructions. The sonicated chromatin was cleared by centrifugation, and diluted in dilution buffer (50 mM Tris-Cl [pH 8.0], 10 mM EDTA, 1% Triton X-100, 0.1% Na-deoxycholate, supplemented with a protease inhibitor cocktail). Five percent of each sample was kept as input. The remaining samples were incubated overnight at 4°C with 1 µg of the following antibodies: anti-FLAG (GS20002, Mabuns), anti-RNA polymerase II (Pol II) (A11181, Abclonal), anti-H3K27me3 (#9733, Cell Signaling), anti-H3K9me3 (A22295, Abclonal), anti-H3K4me3 (A22146, Abclonal), mouse control IgG (sc-2025, Santa Cruz), or rabbit control IgG (30000–0-AP, Proteintech), followed by incubation with 30 μL of magnetic protein A/G beads (Bio-Linkedin, Wuhan, China) or anti-FLAG beads (Mabuns, Wuhan, China). The beads were washed sequentially with washing buffer I (20 mM Tris-HCl [pH 8.0], 2 mM EDTA [pH 8.0], 150 mM NaCl, 1% Triton X-100, 0.1% SDS) twice, washing buffer II (20 mM Tris-HCl [pH 8.0], 2 mM EDTA [pH 8.0], 500 mM NaCl, 1% Triton X-100, 0.1% SDS) twice, washing buffer III (10 mM Tris-HCl [pH 8.0], 1 mM EDTA [pH 8.0], 250 mM LiCl, 1% Triton X-100, 0.1% Na-deoxycholate) twice, and finally once with TE buffer. DNA was eluted with elution buffer (10 mM Tris-Cl [pH 8.0], 1 mM EDTA, 1% SDS), and incubated overnight at 65°C to reverse crosslinking, followed by digestion with Proteinase K and RNase A. The samples were purified by phenol-chloroform extraction, and purified DNA was analyzed by quantitative PCR (qPCR). ChIP-qPCR primers are listed in S2 Table.

### Immunofluorescence

*SMCHD1*-knockdown U2OS cells grown on glass coverslips in 12-well plates were transfected with GFP-SMCHD1 or the indicated mutants for 24 h. Cells were either mock-infected or infected with HSV-1 WT (MOI = 3) or HSV-1 ICP0 RFm (MOI = 3) for 2 h or 6 h. After washing three times with PBS, cells were fixed in 4% paraformaldehyde (PFA) for 15 min, permeabilized with 0.5% Triton X-100 in PBS for 5 min, and blocked with 10% goat serum for 1 h at room temperature. Cells were then incubated overnight at 4°C with primary antibodies against ICP0 (sc-53070; 1:50), ICP8 (sc-53329; 1:50), ICP4 (sc-69809, 1:50), or PML (A27714; 1:50) followed by incubation with Alexa Fluor 594–conjugated anti-mouse secondary antibody or Alexa Fluor 643–conjugated anti-rabbit secondary antibody (Invitrogen; 1:1000) for 1 h at room temperature. Nuclei were counterstained with DAPI (SouthernBiotech). Images were acquired using a Zeiss LSM 980 confocal microscope and processed with ZEN 3.1 software.

### Analysis of HSV-1 genome localization by click chemistry

For EdC (A638729, Aladdin, Shanghai, China) labelling of the HSV-1 genome, U2OS cells maintained in DMEM supplemented with 2% FBS were infected with HSV-1 ICP0 RFm (MOI = 0.1). EdC was added at 6 h post-infection to a final concentration of 5 μM, with fresh EdC replenished every 24 h until full cytopathic effect was observed. The supernatants containing labelled viral particles were collected, and viral titer was determined by standard plaque assay [43,60].

For click chemistry-based imaging of viral genomic DNA, U2OS cells with stable SMCHD1 knockdown were seeded on glass coverslips in 12-well plates overnight. Cells were transfected with GFP-SMCHD1 WT, Δhinge, or 3KR mutant for 24 h, followed by infection with EdC labelled HSV-1 ICP0 RFm (MOI = 3). Specifically, cells were incubated with the virus for 1 h at 4°C, followed by an additional 2 h incubation at 37°C in the presence of cycloheximide (CHX, 100 μg/mL). After infection, cells were washed twice with PBS, fixed in 4% PFA for 10 min, permeabilized with 0.5% Triton X-100 for 10 min, and blocked with 10% goat serum in PBS for 30 min. EdC-labelled viral DNA was detected using copper(I)-catalyzed azide–alkyne cycloaddition (CuAAC) as previously described [60,73]. In brief, the samples were incubated with a click chemistry reaction mixture containing 1 mM CuSO4 (7758-99-8, Sinopharm Chemical, Shanghai, China), 10 mM amino-guanidine (A151036, Aladdin, Shanghai, China), 1 mM THPTA (T405015, Aladdin), 10 mM sodium ascorbate (S105026, Aladdin), and 10 μM Azide AF594 (ST3403, Beyotime Biotech, Shanghai, China) for 2 h at room temperature in the dark. Nuclei were counterstained with DAPI, and images were acquired using a Zeiss LSM 980 confocal microscope as described in the immunofluorescence section.

### Quantification of viral genomes

U2OS or HFF cells were infected with HSV-1 WT, ΔICP0, or ICP0 RFm (MOI = 0.1 or 3) for the indicated times. Intracellular DNA was extracted using the TIANamp Genomic DNA Kit (DP304; TIANGEN, Beijing, China) according to the manufacturer’s instructions. Briefly, cells were lysed in GA buffer and digested with Proteinase K, followed by addition of GB buffer and incubation at 70°C for 10 min. Absolute ethanol was then added, and DNA was purified using adsorption columns, washed twice with wash buffer, and eluted with 50 μL of elution buffer. Viral DNA present in the cell culture supernatant was extracted as previously described [27]. Ten nanograms of purified genomic DNA was used as the template for quantitative PCR (qPCR) targeting the HSV-1 UL19 coding region. Primer sequences are listed in S1 Table.

### Immunoprecipitation

For immunoprecipitation of ectopically expressed proteins, HEK293T cells were transfected with the indicated plasmids for 36 h and lysed in NP-40 buffer (50 mM Tris-HCl [pH 7.5], 150 mM NaCl, 1% NP-40, 1 mM EDTA) supplemented with a protease inhibitor cocktail. Lysates were clarified by centrifugation at 12,000 rpm for 10 min at 4°C, and the supernatants were incubated with 30 μL of anti-FLAG or anti-HA beads (Mabuns) for 5 h at 4°C.

For immunoprecipitation of endogenous proteins, U2OS cells were infected with HSV-1 (MOI = 3) in the presence of MG132 (10 μM) for 4 h. Lysates were prepared as described above and incubated with rabbit IgG (Proteintech) or anti-SMCHD1 antibody (2 μg; 25589–1-AP, Proteintech) for 4 h, followed by incubation with protein A/G magnetic beads (Bio-Linkedin) for 12 h at 4°C.

For experiments with U2OS cells stably expressing tetracycline-inducible FLAG-ICP0, cells were mock-treated or treated with doxycycline (1 μg/mL) for 12 h, followed by MG132 treatment (10 μM) for an additional 12 h. Lysates were prepared as described above and incubated with anti-FLAG beads overnight at 4°C.

All beads (anti-FLAG, anti-HA, or protein A/G) were washed four times with NP-40 lysis buffer, and bound proteins were eluted by boiling in SDS sample buffer for 15 min at 95°C, followed by immunoblotting analysis.

### GST pull-down assay

GST and GST-ICP0 (aa 1–110) were expressed in *Escherichia coli* BL21-DE3 cells by induction with 0.2 mM isopropyl-β-D-thiogalactopyranoside (IPTG) at 18°C overnight. Cells were harvested and lysed by sonication in lysis buffer (150 mM NaCl, 50 mM Tris-HCl, pH 8.0, 10% Glycerol, supplemented with protease inhibitors). The supernatants were collected and incubated with glutathione beads (Smart-Lifesciences, Changzhou, China) for 5 h at 4°C, followed by three washes with wash buffer (150 mM NaCl, 50 mM Tris-HCl [pH 8.0]). HEK293T cells were transfected with FLAG-SMCHD1 for 48 h, harvested, and lysed in lysis buffer (50 mM Tris-HCl [pH 7.5], 150 mM NaCl, 1% NP-40, 1 mM EDTA, supplemented with a protease inhibitor cocktail). The supernatants were collected and incubated with anti-FLAG beads (Mabuns), followed by three washes with lysis buffer. Bound FLAG-SMCHD1 was eluted using 3 x FLAG peptide diluted in PBS (T510425-0010, Sangon Biotech). Purified FLAG-SMCHD1 was incubated with GST or GST-ICP0 (aa 1–110) immobilized on glutathione beads. After incubation, bound fractions were eluted by boiling in SDS loading buffer and analyzed by immunoblotting.

### Ubiquitination assay

For ubiquitination of ectopically expressed SMCHD1, HEK293T cells were transfected with the indicated plasmids for 24 h, followed by treatment with MG132 (10 μM) for an additional 12 h. Cells were lysed in lysis buffer (50 mM Tris-HCl [pH 7.4], 150 mM NaCl, 1% Triton X-100, 1 mM EDTA, supplemented with a protease inhibitor cocktail). The supernatants were mixed with 1% SDS and boiled at 95°C for 10 min to denature proteins. After cooling on ice, the samples were diluted 10-fold with lysis buffer to reduce the SDS concentration to 0.1%, and then incubated with 30 μL of anti-FLAG beads for 6 h at 4°C.

For ubiquitination of endogenous SMCHD1, U2OS cells were infected with HSV-1 (MOI = 1) in the presence of MG132 (10 μM) for 6 h, or treated with doxycycline (1 μg/mL) for 24 h to induce the expression of ICP0 WT or RFm. Cell lysates were prepared as described above, and immunoprecipitation was performed by incubating with rabbit control IgG (Proteintech) or anti-SMCHD1 antibody (2 μg; 25589–1-AP, Proteintech) for 4 h at 4°C, followed by incubation with 30 μL of protein A/G magnetic beads (Bio-Linkedin) for 12 h at 4°C.

The beads (anti-FLAG or protein A/G magnetic beads) were washed four times with lysis buffer. Bound proteins were eluted by boiling in 1% SDS loading buffer, and analyzed by immunoblotting.

For protein purification, His-SMCHD1 (aa 1601–2005), either WT or mutant (K1958R/K1976R), was expressed in *E. coli* BL21-DE3 by induction with 0.2 mM IPTG at 18°C overnight. Cells were harvested and lysed by sonication in lysis buffer (50 mM Tris-Cl [pH 7.5], 150 mM NaCl, 10% glycerol, 10 mM imidazole, 1% Triton X-100, supplemented with protease inhibitors). Lysates were clarified by centrifugation, and the supernatants were incubated with Ni-NTA agarose beads (Smart-Lifesciences, Changzhou, China) for 5 h at 4°C. After incubation, beads were washed three times with washing buffer (50 mM Tris-Cl [pH 7.5], 150 mM NaCl, 10% glycerol, 20 mM imidazole) and used for in vitro ubiquitination reactions.

GST-ICP0 (aa 1–241) was purified from *E. coli* BL21-DE3 using the same procedure as GST fusion protein purification as previously described, and was eluted with elution buffer (150 mM NaCl, 50 mM Tris-HCl [pH 8.0], 15 mM reduced glutathione).

For in vitro ubiquitination assay, 500 ng of His-SMCHD1 (aa 1601–2005) WT or mutant (K1958R/K1976R) was incubated with E1 (UBE1, 40 ng; #20433ES25, YEASEN), E2 (UBE2D1, 200 ng; #20434ES65, YEASEN), ubiquitin (5 μg; #20431ES08, YEASEN), and ATP (2 mM) in the presence of GST (50 ng) or GST-ICP0 (aa 1–241) (50 ng) in reaction buffer (50 mM Tris-HCl [pH 7.5], 50 mM NaCl, and 1 mM DTT) for 1 h at 37°C. Reactions were terminated by adding SDS loading buffer and boiling at 95°C for 5 min, followed by immunoblotting analysis.

### SUMOylation assay

For detection of exogenous SUMOylated SMCHD1, HEK293T cells were cotransfected with FLAG-SMCHD1 WT or its mutants together with MYC-UBC9 and His-SUMO1/2/3 for 24 h, followed by infection with HSV-1 ICP0 RFm (MOI = 5) for an additional 24 h. For detection of endogenous SUMOylated SMCHD1, HFF cells were infected with HSV-1 ICP0 RFm (MOI = 5) for 24 h.

SUMOylated SMCHD1, derived from both exogenous and endogenous sources, was detected under denaturing conditions. In brief, cells were collected and lysed in denaturing lysis buffer (50 mM Tris-HCl, pH 6.8, 1% SDS, 40 mM DTT, 20 μM NEM, 5% glycerol, supplemented with a protease inhibitor cocktail), sonicated until lysate became homogeneous, and then boiled for 10 min. After centrifugation at 12,000 g for 10 min at 4°C, 10% of the supernatant was kept as input. The remaining lysate was diluted 10-fold with lysis buffer (50 mM Tris-HCl [pH 7.4], 150 mM NaCl, 40 mM DTT, 20 μM NEM, and 5% glycerol). The diluted samples were incubated with anti-FLAG beads or with protein A/G beads together with rabbit control IgG (2 µg), anti-SUMO1 antibody (2 µg; A19121, Abclonal), or anti-SUMO2/3 antibody (2 µg; A22734, Abclonal) overnight at 4°C. Beads were washed four times with lysis buffer. Immunoprecipitates were eluted by boiling in 1% SDS loading buffer and analyzed by immunoblotting.

### Western blot and densitometry analysis

Cells were washed with cold PBS two times and lysed in lysis buffer (50 mM Tris-HCl, pH 7.4, 150 mM NaCl, 1% NP-40) supplemented with a protease inhibitor cocktail. Equal amounts of protein were resolved by SDS–PAGE and transferred onto nitrocellulose (NC) membranes (0.45 μm, Millipore) for immunoblotting. NC membranes were blocked with 5% skimmed milk in Tris-buffered saline containing 0.1% Tween-20 (TBST) for 0.5 h at room temperature and incubated with primary antibodies overnight at 4°C. After three washes with TBST, membranes were incubated with IRDye-conjugated secondary antibodies for 1 h at room temperature. Protein bands were visualized using Image Studio software (LI-COR).

For densitometric analysis, band intensities were quantified using Fiji software. Band intensities were normalized to the corresponding loading control (β-Actin), and relative protein levels were calculated accordingly. Densitometric quantification shown in the figures was derived from at least three independent biological replicates.

### Statistical analysis

Data represent the means of at least three independent experiments, and error bars denote standard deviations (SD). An unpaired two-tailed Student’s t test or analysis of variance (ANOVA) was used for statistical analysis. GraphPad Prism 8 (v.8.0.2) was used for all statistical analysis. Significant differences are represented by p value: *, p < 0.05; **, p < 0.01; ***, p < 0.005.

## Supporting information

S1 Fig SMCHD1 restricts HSV-1 replication. (A-C) U2OS cells transduced with control shRNA or shRNA targeting *SMCHD1* were infected with HSV-1 (MOI = 0.1), and WCLs were analyzed by immunoblotting at 24 h post-infection (A). Viral gene expression was determined by qRT-PCR at 24 h post-infection (B), and viral titers were quantified at the indicated time points (C). (D-F) U2OS cells transduced with control vector or FLAG-SMCHD1 through lentiviral transduction were infected with HSV-1 (MOI = 1). WCLs were analyzed by immunoblotting at the indicated time points post-infection (D). Viral gene expression was quantified by qRT-PCR at 24 h post-infection (E), and viral titers were quantified at 48 h post-infection (F). Data are presented as mean ± SD from three independent experiments (n = 3). Statistical significance was determined by unpaired two tailed Student’s t test or two-way ANOVA. p value: *, p < 0.05; **, p < 0.01; ***, p < 0.005. N.S.: no significance.
(TIF)

S2 Fig HSV-1 ICP0 targets SMCHD1 for proteasomal degradation. (A) U2OS cells were infected with HSV-1 (MOI = 3). WCLs were analyzed by immunoblotting at the indicated time points post-infection. (B) U2OS cells were infected with HSV-1 (MOI = 1), and *SMCHD1* mRNA levels were quantified by qRT-PCR at 24 h post-infection. (C) U2OS cells were infected with HSV-1 (MOI = 1), and PAA (200 μg/mL) was added at 1 h post-infection. WCLs were analyzed by immunoblotting at 24 h post-infection. (D) U2OS cells were infected with HSV-1 (MOI = 1), ΔICP0 (MOI = 3), ΔICP22 (MOI = 3), ΔICP27 (MOI = 3), or ΔICP47/ΔICP34.5 (MOI = 3) for 24 h. WCLs were analyzed by immunoblotting. (E) U2OS cells were infected with HSV-1 (MOI = 3) or ΔICP0 (MOI = 3), and WCLs were analyzed by immunoblotting at the indicated time points post-infection. (F) U2OS cells transduced with control lentivirus or lentivirus containing tetracycline-inducible FLAG-ICP0 gene were treated with Dox (1 μg/mL) for 12 h, followed by MG132 (10 μM) or BafA1 (2 μM) treatment for another 12 h. WCLs were analyzed by immunoblotting. (G) Control and tetracycline-inducible FLAG-ICP0 U2OS cells were transduced with control sgRNA or sgRNA targeting *UBE2I* to generate stable cells. The stable cells were treated with Dox (1 μg/mL) for 24 h, and WCLs were analyzed by immunoblotting. (H-I) Schematic diagram of SMCHD1 full-length and truncated mutants (H). U2OS transduced with FLAG-SMCHD1 or truncated mutants through lentiviral transduction were infected with HSV-1 (MOI = 1) or HSV-1 ΔICP0 (MOI = 3) for 24 h, and WCLs were analyzed by immunoblotting (I). (J) HEK293T cells were co-transfected with FLAG-SMCHD1 or its mutants, MYC-ICP0 and HA-Ub for 24 h, followed by MG132 (10 μM) treatment for another 12 h. Denaturing immunoprecipitation was performed using anti-FLAG beads, followed by immunoblotting. Data are presented as mean ± SD from three independent experiments (n = 3). Statistical significance was determined by unpaired two tailed Student’s t test. p value: *, p < 0.05; **, p < 0.01; ***, p < 0.005. N.S.: no significance.
(TIF)

S3 Fig HSV-1 ICP0 interacts with SMCHD1. (A) *SMCHD1*-knockdown U2OS cells were transfected with GFP-SMCHD1 for 24 h and then infected with HSV-1 WT (MOI = 3) for 2 h. Immunofluorescence staining was performed using anti-ICP0 and anti-PML antibodies. Scale bars, 5 μm. (B) U2OS cells transduced with control sgRNA or sgRNA targeting *RNF8* or *RNF168* to generate stable cells. The stable cells were infected with HSV-1 (MOI = 3) for 24 h, and WCLs were analyzed by immunoblotting. (C) U2OS cells stably expressing tetracycline-inducible FLAG-ICP0 WT or FLAG-ICP0 T67A mutant were induced with Dox (1 μg/mL) for 24 h. WCLs were analyzed by immunoblotting.
(TIF)

S4 Fig HSV-1 infection induces SUMOylation of SMCHD1. (A) U2OS cells stably transduced with control sgRNA or sgRNA targeting *UBE2I* were infected with HSV-1 ΔICP0 (MOI = 5) for 24 h, and WCLs were analyzed by immunoblotting. (B) HEK293T or HFF cells were infected with HSV-1 WT, ICP0 RFm or ΔICP0 at an MOI of 0.1, and viral titers were determined at the indicated time points post-infection. (C) HEK293T cells were co-transfected with FLAG-SMCHD1, UBC9 and SUMO1–3 expression plasmids for 24 h, and WCLs were analyzed by immunoblotting. (D-F) HEK293T cells were co-transfected with the indicated plasmids for 24 h, followed by infection with HSV-1 ICP0 RFm (RING finger mutant) (MOI = 5) for an additional 24 h. Denaturing immunoprecipitation was carried out using FLAG beads, followed by immunoblotting. Data are presented as mean ± SD from three independent experiments (n = 3). Statistical significance was determined by two-way ANOVA. p value: *, p < 0.05; **, p < 0.01; ***, p < 0.005. N.S.: no significance.
(TIF)

S5 Fig SUMOylation of the SMCHD1 hinge domain confers antiviral restriction. (A-E) *SMCHD1*-knockdown U2OS cells were stably reconstituted with vector control, *SMCHD1* WT, or the indicated mutants (Δhinge, K1848R, K1852R, K1872R, or the 3KR mutant [K1848R/K1852R/K1872R]) through lentiviral transduction. WCLs were analyzed by immunoblotting (A). The reconstituted cells were infected with HSV-1 (MOI = 0.1), viral gene expression was quantified by qRT-PCR at 24 h post-infection (B-D), and viral titers were determined at 48 h post-infection (E). Data are presented as mean ± SD from three independent experiments (n = 3). Statistical significance was determined by one-way ANOVA. p value: *, p < 0.05; **, p < 0.01; ***, p < 0.005. N.S.: no significance.
(TIF)

S6 Fig SMCHD1 associates with HSV-1 genome to repress viral gene transcription. (A) Control or *SMCHD1*-knockdown U2OS cells were infected with HSV-1 ICP0 RFm (MOI = 3) in the presence of PAA (200 μg/mL) to block viral DNA replication. WCLs were analyzed by immunoblotting. (B) HFF cells were infected with HSV-1 ICP0 RFm (MOI = 5) for 4 h, followed by ChIP assay using anti-SMCHD1 antibody or control IgG. (C) U2OS cells were transduced with control sgRNA or sgRNA targeting *UBE2I* to generate stable cells. The stable cells were infected with HSV-1 ICP0 RFm (MOI = 5), and ChIP assays were performed at 4 h post-infection using anti-SMCHD1 antibody. (D) *SMCHD1*-knockdown HFF cells stably reconstituted with *SMCHD1* WT or mutants (Δhinge and 3KR [K1848R/K1852R/K1872R]) were infected with HSV-1 ICP0 RFm (MOI = 5), and ChIP assays were performed at 4 h post-infection using anti-FLAG antibody. (E) *SMCHD1*-knockdown U2OS cells were transfected with GFP-tagged SMCHD1 K1848R, K1852R, or K1872R mutants, followed by immunofluorescence analysis. Scale bars, 5 μm. (F) *SMCHD1*-knockdown U2OS cells stably reconstituted with FLAG-tagged SMCHD1 WT, K1848R, K1852R, or K1872R mutants were infected with HSV-1 ICP0 RFm (MOI = 5), and ChIP assays were performed at 4 h post-infection using anti-FLAG antibody. (G, H) *SMCHD1*-knockdown U2OS cells were transfected with GFP-SMCHD1 for 24 h and infected with HSV-1 (MOI = 3) or HSV-1 ICP0 RFm (MOI = 3). Immunofluorescence using anti-ICP4 antibody was performed at 2 h post-infection (G). Immunofluorescence using anti-ICP8 antibody was performed at 6 h post-infection (H). Scale bars, 5 μm. (I, J) Control or *SMCHD1*-knockdown U2OS cells were infected with HSV-1 ICP0 RFm (MOI = 5) for 4 h, and ChIP analysis was performed using antibodies against H3K9me3 or H3K27me3 (I), or H3K4me3 (J). (K, L) *SMCHD1*-knockdown U2OS cells stably reconstituted with vector control, SMCHD1 WT or mutants (Δhinge and 3KR) were infected with HSV-1 ICP0 RFm (MOI = 5) for 4 h, followed by ChIP assays with antibodies against H3K9me3 or H3K27me3 (K), or H3K4me3 (L). Data are presented as mean ± SD from three independent experiments (n = 3). Statistical significance was determined by two-way ANOVA. p value: *, p < 0.05; **, p < 0.01; ***, p < 0.005. N.S.: no significance.
(TIF)

S7 Fig ICP0 counteracts SMCHD1-mediated restriction of HSV-1 replication. (A) Plaque assays were performed using U2OS cells. Viral supernatants were normalized to equivalent titers, and then subjected to viral DNA extraction with or without DNase I treatment. Viral genome copy numbers were subsequently quantified by qPCR, and the ratio of viral genome copies to PFU was calculated. (B, C) U2OS stable cells transduced with control shRNA or shRNA targeting *SMCHD1* were infected with HSV-1 (MOI = 0.1), HSV-1 ICP0 RFm (MOI = 0.1) or HSV-1 ΔICP0 (MOI = 0.1). Viral genome copy numbers were quantified by qPCR at the indicated time points post-infection (B). Viral titers were quantified for the indicated times post-infection (C). (D) U2OS cells were infected with HSV-1 WT, ICP0 RFm, or ΔICP0 at an MOI of 0.1, and viral titers were determined at the indicated time points post-infection. (E) U2OS stable cells transduced with vector or FLAG-SMCHD1 were infected with HSV-1 (MOI = 0.1) or HSV-1 ΔICP0 (MOI = 0.1), and viral titers were quantified at 48 h post-infection. Data are presented as mean ± SD from three independent experiments (n = 3). Statistical significance was determined by unpaired two tailed Student’s t test or two-way ANOVA. p value: *, p < 0.05; **, p < 0.01; ***, p < 0.005. N.S.: no significance.
(TIF)

S1 Table qRT-PCR primers. (DOCX)

S2 Table ChIP-qPCR primers. (DOCX)
