# Supplementary figures and images for: SUMOylation and ubiquitination reciprocally regulate SMCHD1 antiviral activity against herpes simplex virus 1
Source: PLoS Pathog. 2026 Jun 24;22(6):e1014371. doi: 10.1371/journal.ppat.1014371 (PMC13313348; doi:10.1371/journal.ppat.1014371)

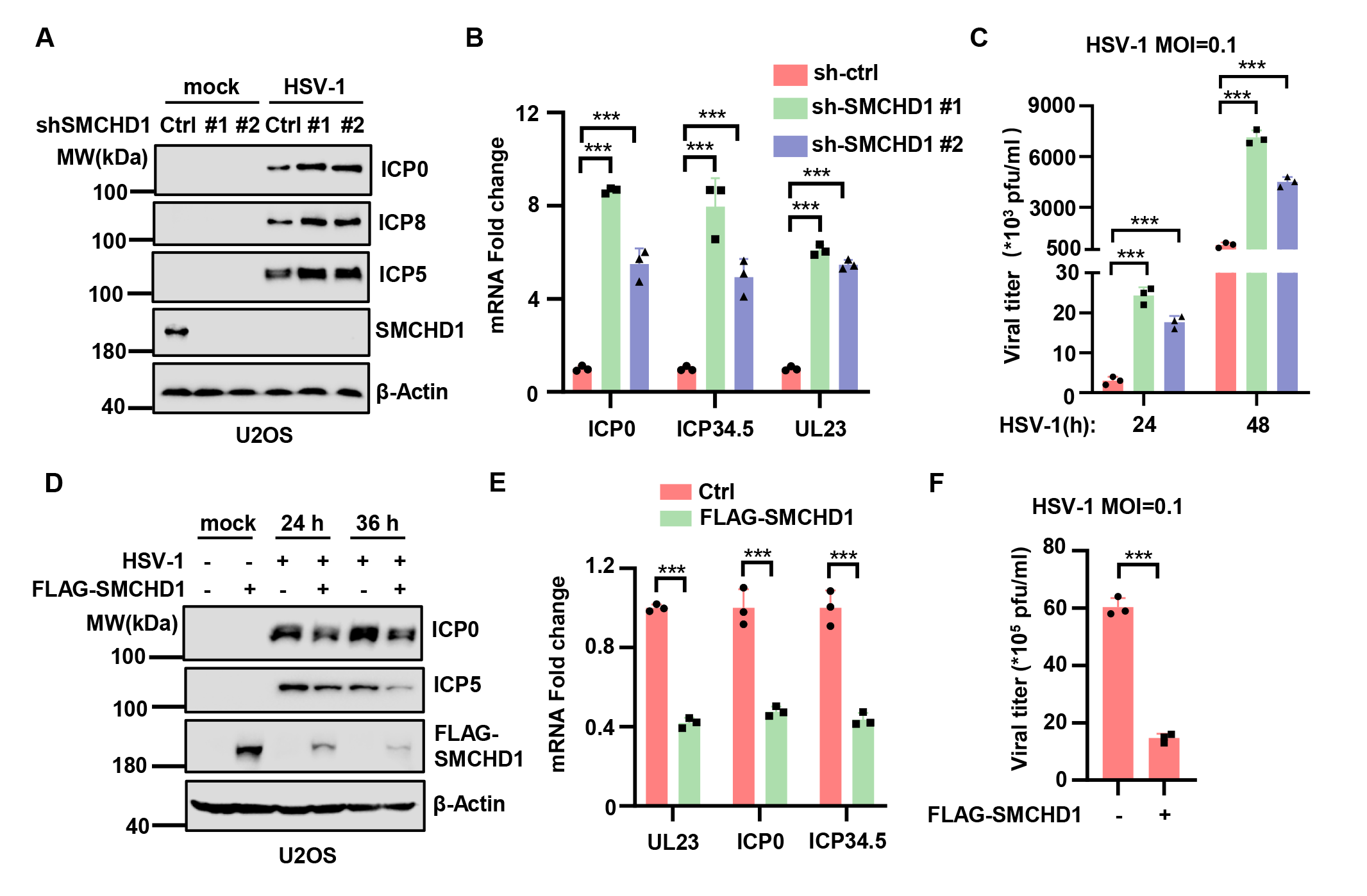

Supplement: S1 Fig — (A-C) U2OS cells transduced with control shRNA or shRNA targeting SMCHD1 were infected with HSV-1 (MOI = 0.1), and WCLs were analyzed by immunoblotting at 24 h post-infection (A). Viral gene expression was determined by qRT-PCR at 24 h post-infection (B), and viral titers were quantified at the indicated time points (C). (D-F) U2OS cells transduced with control vector or FLAG-SMCHD1 through lentiviral transduction were infected with HSV-1 (MOI = 1). WCLs were analyzed by immunoblotting at the indicated time points post-infection (D). Viral gene expression was quantified by qRT-PCR at 24 h post-infection (E), and viral titers were quantified at 48 h post-infection (F). Data are presented as mean ± SD from three independent experiments (n = 3). Statistical significance was determined by unpaired two tailed Student’s t test or two-way ANOVA. p value: *, p < 0.05; **, p < 0.01; ***, p < 0.005. N.S.: no significance. (TIF) [file ppat.1014371.s001.tif]

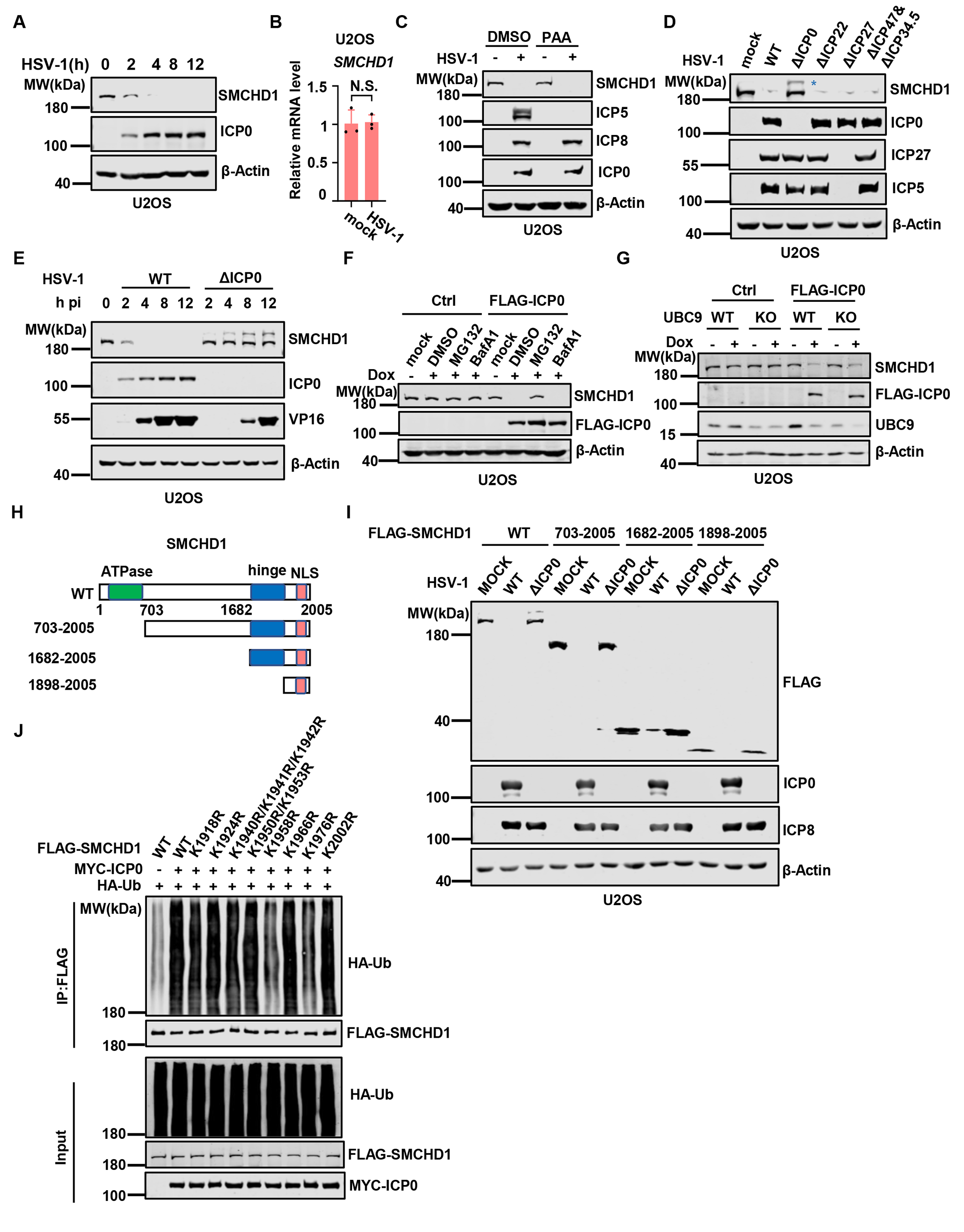

Supplement: S2 Fig — (A) U2OS cells were infected with HSV-1 (MOI = 3). WCLs were analyzed by immunoblotting at the indicated time points post-infection. (B) U2OS cells were infected with HSV-1 (MOI = 1), and SMCHD1 mRNA levels were quantified by qRT-PCR at 24 h post-infection. (C) U2OS cells were infected with HSV-1 (MOI = 1), and PAA (200 μg/mL) was added at 1 h post-infection. WCLs were analyzed by immunoblotting at 24 h post-infection. (D) U2OS cells were infected with HSV-1 (MOI = 1), ΔICP0 (MOI = 3), ΔICP22 (MOI = 3), ΔICP27 (MOI = 3), or ΔICP47/ΔICP34.5 (MOI = 3) for 24 h. WCLs were analyzed by immunoblotting. (E) U2OS cells were infected with HSV-1 (MOI = 3) or ΔICP0 (MOI = 3), and WCLs were analyzed by immunoblotting at the indicated time points post-infection. (F) U2OS cells transduced with control lentivirus or lentivirus containing tetracycline-inducible FLAG-ICP0 gene were treated with Dox (1 μg/mL) for 12 h, followed by MG132 (10 μM) or BafA1 (2 μM) treatment for another 12 h. WCLs were analyzed by immunoblotting. (G) Control and tetracycline-inducible FLAG-ICP0 U2OS cells were transduced with control sgRNA or sgRNA targeting UBE2I to generate stable cells. The stable cells were treated with Dox (1 μg/mL) for 24 h, and WCLs were analyzed by immunoblotting. (H-I) Schematic diagram of SMCHD1 full-length and truncated mutants (H). U2OS transduced with FLAG-SMCHD1 or truncated mutants through lentiviral transduction were infected with HSV-1 (MOI = 1) or HSV-1 ΔICP0 (MOI = 3) for 24 h, and WCLs were analyzed by immunoblotting (I). (J) HEK293T cells were co-transfected with FLAG-SMCHD1 or its mutants, MYC-ICP0 and HA-Ub for 24 h, followed by MG132 (10 μM) treatment for another 12 h. Denaturing immunoprecipitation was performed using anti-FLAG beads, followed by immunoblotting. Data are presented as mean ± SD from three independent experiments (n = 3). Statistical significance was determined by unpaired two tailed Student’s t test. p value: *, p < 0.05; **, p < 0.01; ***, p < 0.0 [file ppat.1014371.s002.tif]

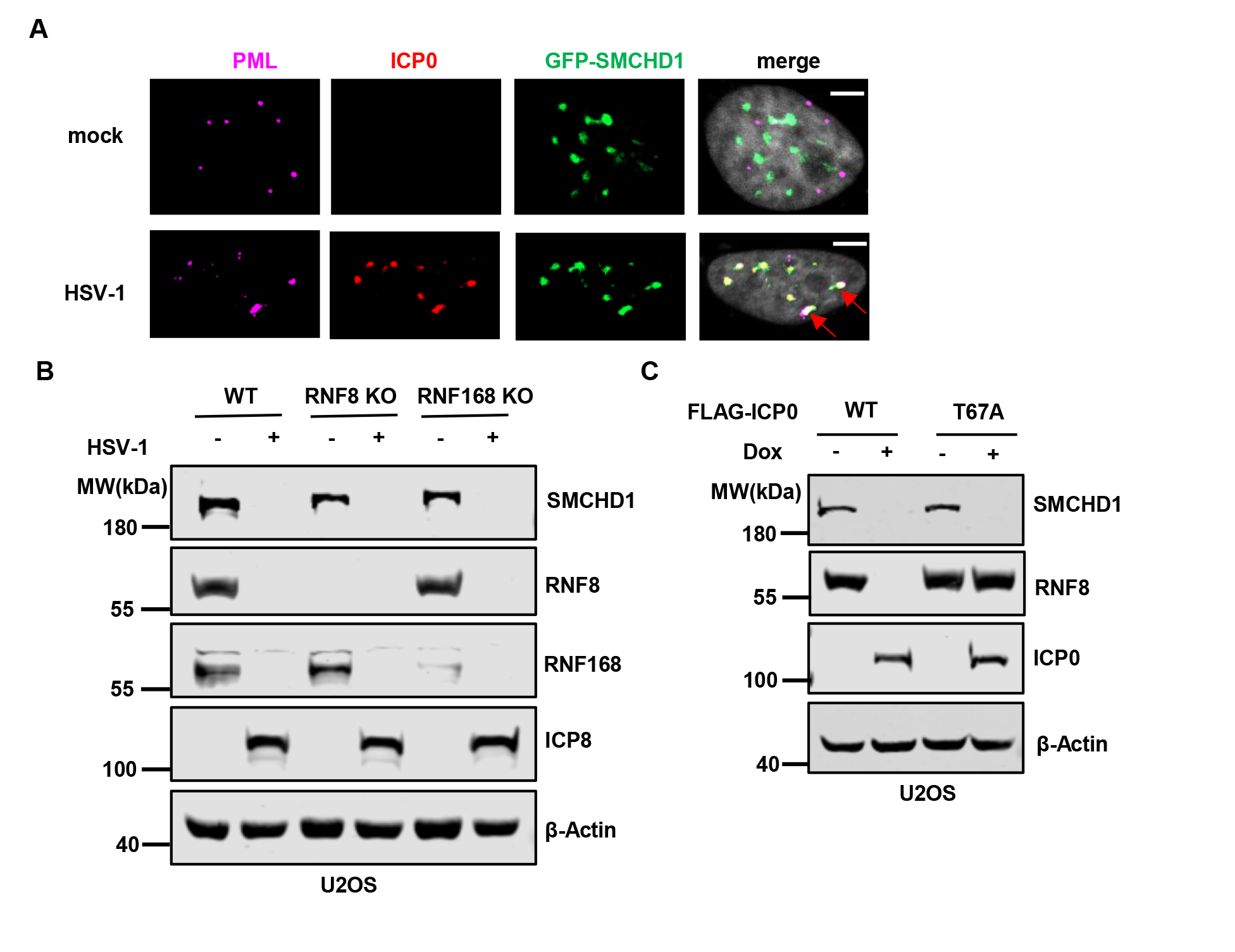

Supplement: S3 Fig — (A) SMCHD1-knockdown U2OS cells were transfected with GFP-SMCHD1 for 24 h and then infected with HSV-1 WT (MOI = 3) for 2 h. Immunofluorescence staining was performed using anti-ICP0 and anti-PML antibodies. Scale bars, 5 μm. (B) U2OS cells transduced with control sgRNA or sgRNA targeting RNF8 or RNF168 to generate stable cells. The stable cells were infected with HSV-1 (MOI = 3) for 24 h, and WCLs were analyzed by immunoblotting. (C) U2OS cells stably expressing tetracycline-inducible FLAG-ICP0 WT or FLAG-ICP0 T67A mutant were induced with Dox (1 μg/mL) for 24 h. WCLs were analyzed by immunoblotting. (TIF) [file ppat.1014371.s003.tif]

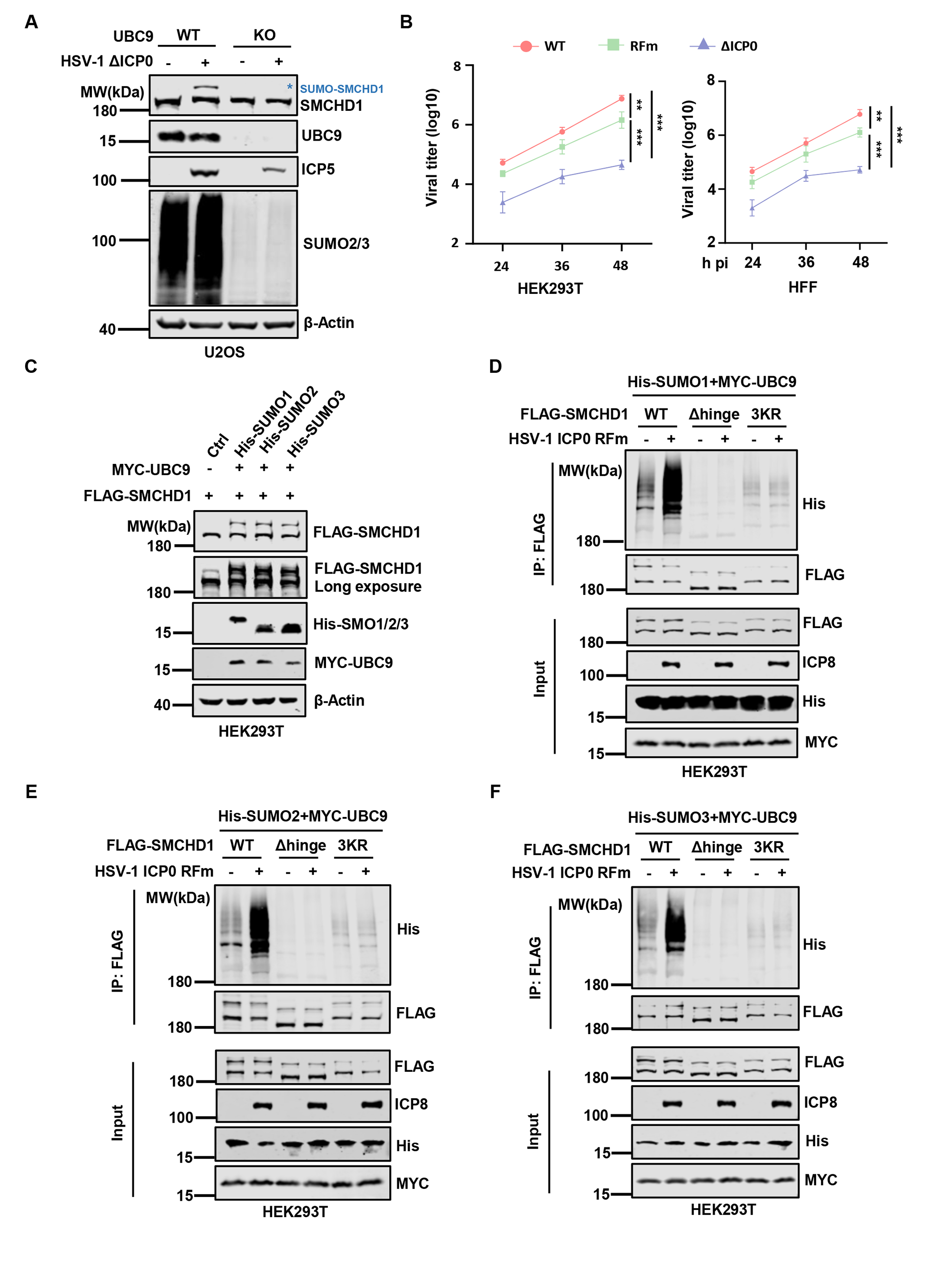

Supplement: S4 Fig — (A) U2OS cells stably transduced with control sgRNA or sgRNA targeting UBE2I were infected with HSV-1 ΔICP0 (MOI = 5) for 24 h, and WCLs were analyzed by immunoblotting. (B) HEK293T or HFF cells were infected with HSV-1 WT, ICP0 RFm or ΔICP0 at an MOI of 0.1, and viral titers were determined at the indicated time points post-infection. (C) HEK293T cells were co-transfected with FLAG-SMCHD1, UBC9 and SUMO1–3 expression plasmids for 24 h, and WCLs were analyzed by immunoblotting. (D-F) HEK293T cells were co-transfected with the indicated plasmids for 24 h, followed by infection with HSV-1 ICP0 RFm (RING finger mutant) (MOI = 5) for an additional 24 h. Denaturing immunoprecipitation was carried out using FLAG beads, followed by immunoblotting. Data are presented as mean ± SD from three independent experiments (n = 3). Statistical significance was determined by two-way ANOVA. p value: *, p < 0.05; **, p < 0.01; ***, p < 0.005. N.S.: no significance. (TIF) [file ppat.1014371.s004.tif]

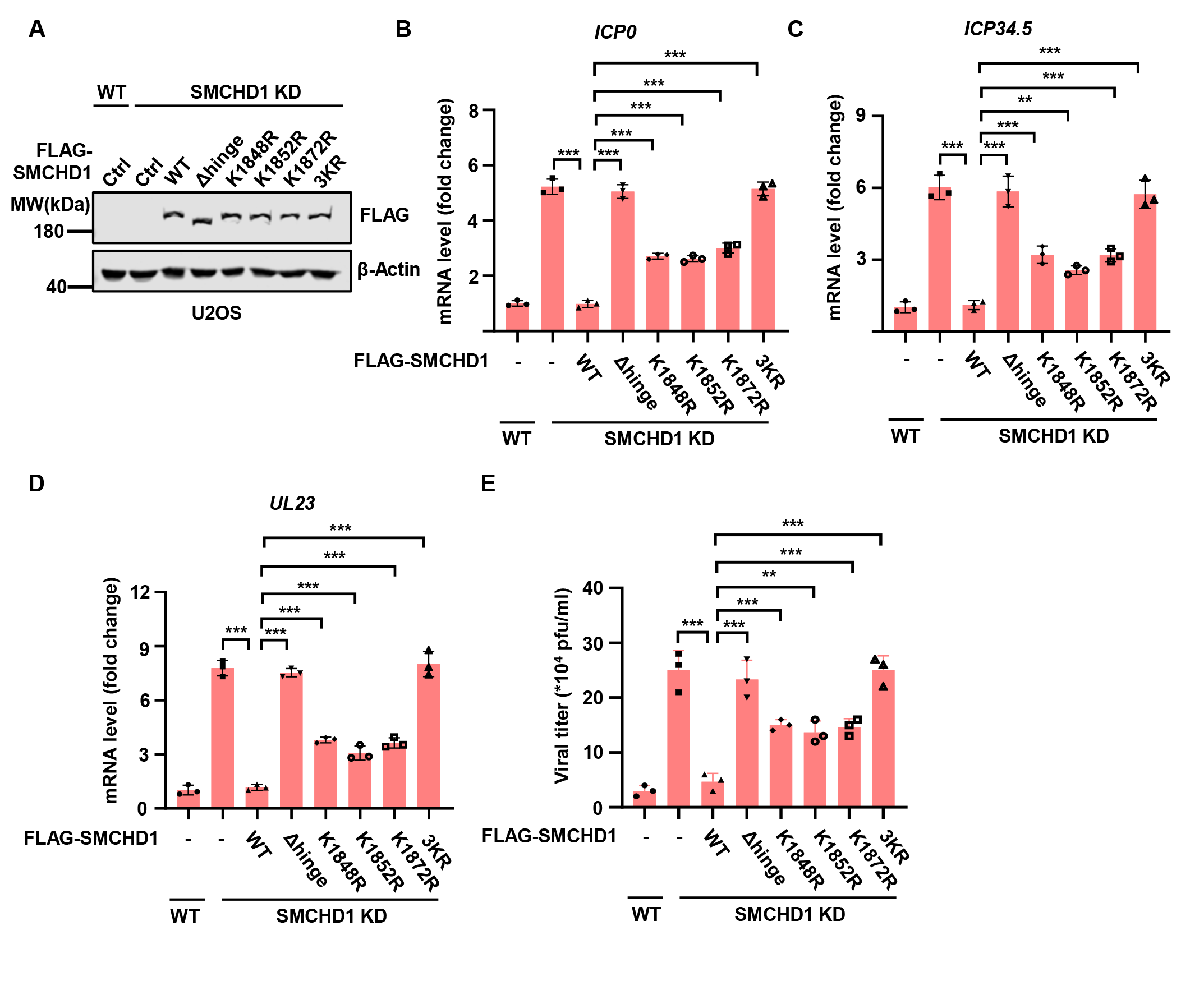

Supplement: S5 Fig — (A-E) SMCHD1-knockdown U2OS cells were stably reconstituted with vector control, SMCHD1 WT, or the indicated mutants (Δhinge, K1848R, K1852R, K1872R, or the 3KR mutant [K1848R/K1852R/K1872R]) through lentiviral transduction. WCLs were analyzed by immunoblotting (A). The reconstituted cells were infected with HSV-1 (MOI = 0.1), viral gene expression was quantified by qRT-PCR at 24 h post-infection (B-D), and viral titers were determined at 48 h post-infection (E). Data are presented as mean ± SD from three independent experiments (n = 3). Statistical significance was determined by one-way ANOVA. p value: *, p < 0.05; **, p < 0.01; ***, p < 0.005. N.S.: no significance. (TIF) [file ppat.1014371.s005.tif]

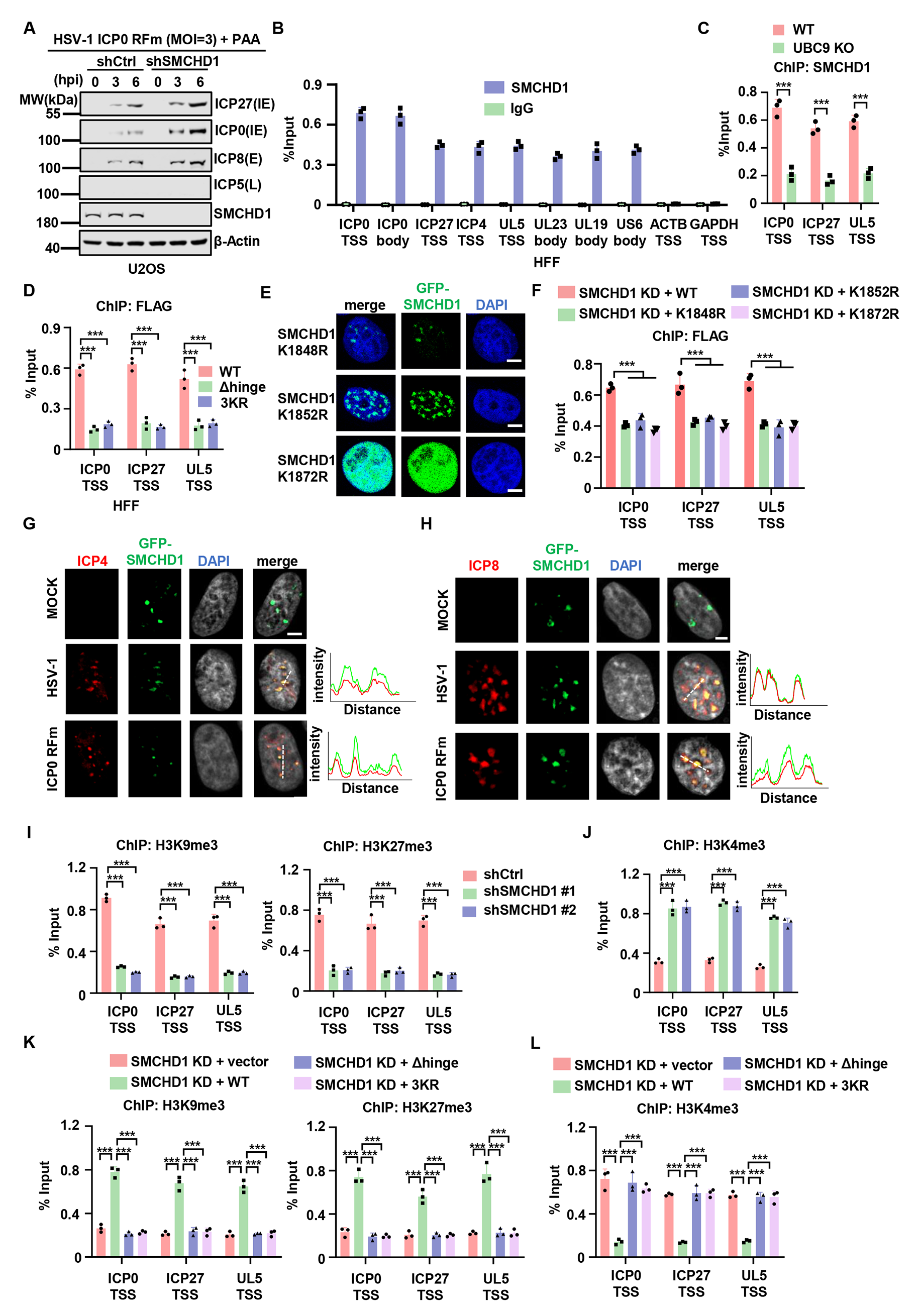

Supplement: S6 Fig — (A) Control or SMCHD1-knockdown U2OS cells were infected with HSV-1 ICP0 RFm (MOI = 3) in the presence of PAA (200 μg/mL) to block viral DNA replication. WCLs were analyzed by immunoblotting. (B) HFF cells were infected with HSV-1 ICP0 RFm (MOI = 5) for 4 h, followed by ChIP assay using anti-SMCHD1 antibody or control IgG. (C) U2OS cells were transduced with control sgRNA or sgRNA targeting UBE2I to generate stable cells. The stable cells were infected with HSV-1 ICP0 RFm (MOI = 5), and ChIP assays were performed at 4 h post-infection using anti-SMCHD1 antibody. (D) SMCHD1-knockdown HFF cells stably reconstituted with SMCHD1 WT or mutants (Δhinge and 3KR [K1848R/K1852R/K1872R]) were infected with HSV-1 ICP0 RFm (MOI = 5), and ChIP assays were performed at 4 h post-infection using anti-FLAG antibody. (E) SMCHD1-knockdown U2OS cells were transfected with GFP-tagged SMCHD1 K1848R, K1852R, or K1872R mutants, followed by immunofluorescence analysis. Scale bars, 5 μm. (F) SMCHD1-knockdown U2OS cells stably reconstituted with FLAG-tagged SMCHD1 WT, K1848R, K1852R, or K1872R mutants were infected with HSV-1 ICP0 RFm (MOI = 5), and ChIP assays were performed at 4 h post-infection using anti-FLAG antibody. (G, H) SMCHD1-knockdown U2OS cells were transfected with GFP-SMCHD1 for 24 h and infected with HSV-1 (MOI = 3) or HSV-1 ICP0 RFm (MOI = 3). Immunofluorescence using anti-ICP4 antibody was performed at 2 h post-infection (G). Immunofluorescence using anti-ICP8 antibody was performed at 6 h post-infection (H). Scale bars, 5 μm. (I, J) Control or SMCHD1-knockdown U2OS cells were infected with HSV-1 ICP0 RFm (MOI = 5) for 4 h, and ChIP analysis was performed using antibodies against H3K9me3 or H3K27me3 (I), or H3K4me3 (J). (K, L) SMCHD1-knockdown U2OS cells stably reconstituted with vector control, SMCHD1 WT or mutants (Δhinge and 3KR) were infected with HSV-1 ICP0 RFm (MOI = 5) for 4 h, followed by ChIP assays with antibodies against H3K9me3 or H3K27me3 (K), or H3K4me3 (L). Da [file ppat.1014371.s006.tif]

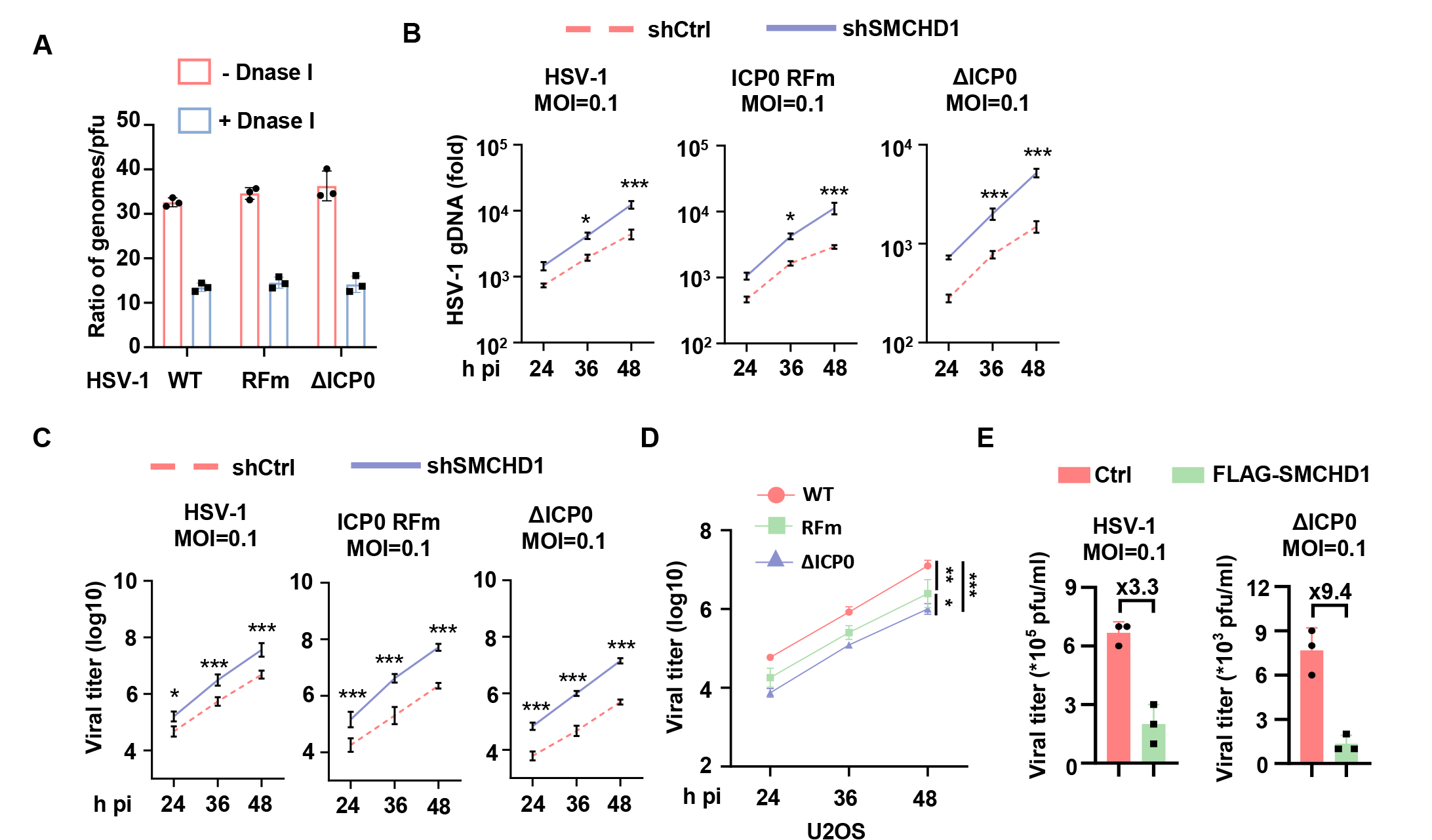

Supplement: S7 Fig — (A) Plaque assays were performed using U2OS cells. Viral supernatants were normalized to equivalent titers, and then subjected to viral DNA extraction with or without DNase I treatment. Viral genome copy numbers were subsequently quantified by qPCR, and the ratio of viral genome copies to PFU was calculated. (B, C) U2OS stable cells transduced with control shRNA or shRNA targeting SMCHD1 were infected with HSV-1 (MOI = 0.1), HSV-1 ICP0 RFm (MOI = 0.1) or HSV-1 ΔICP0 (MOI = 0.1). Viral genome copy numbers were quantified by qPCR at the indicated time points post-infection (B). Viral titers were quantified for the indicated times post-infection (C). (D) U2OS cells were infected with HSV-1 WT, ICP0 RFm, or ΔICP0 at an MOI of 0.1, and viral titers were determined at the indicated time points post-infection. (E) U2OS stable cells transduced with vector or FLAG-SMCHD1 were infected with HSV-1 (MOI = 0.1) or HSV-1 ΔICP0 (MOI = 0.1), and viral titers were quantified at 48 h post-infection. Data are presented as mean ± SD from three independent experiments (n = 3). Statistical significance was determined by unpaired two tailed Student’s t test or two-way ANOVA. p value: *, p < 0.05; **, p < 0.01; ***, p < 0.005. N.S.: no significance. (TIF) [file ppat.1014371.s007.tif]
