# Supplementary material for: SUMOylation and ubiquitination reciprocally regulate SMCHD1 antiviral activity against herpes simplex virus 1
Source: PLoS Pathog. 2026 Jun 24;22(6):e1014371. doi: 10.1371/journal.ppat.1014371 (PMC13313348; doi:10.1371/journal.ppat.1014371)
Supplement: S1 Table — (DOCX) [file ppat.1014371.s008.docx]

qRT-PCR primers

| *ICP0* | GTCGCCTTACGTGAACAAGAC |
| --- | --- |
|  | GTCGCCATGTTTCCCGTCTG |
| ICP27 | GAATCGGACAGCAGCGGGGA |
|  | CCGCCGGGCGAGCGGCGTCG |
| *UL23* | GGAGGACAGACACATCGACC |
|  | TATTGGCAAGCAGCCCGTAA |
| UL30 | CGCCTTGCTTGAGGCAAAAC |
|  | TTCGCTATAGTACGTATGGC |
| *ICP34.5* | GTCCCAGGTAACCTCCACG |
|  | GACGCGGACTCGGGAAC |
| UL19 | GGACCGCTTTGTGACTGAGA |
|  | TGAGCGTGAAGTTTACCCCC |
| *ACTB*  (β-actin) | GTTGTCGACGACGAGCG |
|  | GCACAGAGCCTCGCCTT |
