# Supplementary material for: SUMOylation and ubiquitination reciprocally regulate SMCHD1 antiviral activity against herpes simplex virus 1
Source: PLoS Pathog. 2026 Jun 24;22(6):e1014371. doi: 10.1371/journal.ppat.1014371 (PMC13313348; doi:10.1371/journal.ppat.1014371)
Supplement: S2 Table — (DOCX) [file ppat.1014371.s009.docx]

ChIP-qPCR primers

| ICP0 TSS | agacgcagcagccaggcaga |
| --- | --- |
|  | cgtatgcggctggagggtcg |
| ICP4 TSS | gggcgatcctccggggatac |
|  | ggtcgtcggggtccgtgggt |
| ICP27 TSS | ccaaccacggtcacgcttc |
|  | tgccgtcggggctggggtgt |
| UL5 TSS | tctcacggaacagcatcgta |
|  | acgctcgtgggcccccact |
| ICP0 body | cccagcgcgaggtgaggggc |
|  | ccaacatggcggccggttcc |
| UL19 body | cagggcctcgacgtgcgcca |
|  | gttcgtgaacgactactcgc |
| UL23 body | ccctggtcgaggcggtgttg |
|  | ttcgcgcgacgatatcgtct |
| US6 body | caatcgctttcgcggcaaag |
|  | ctggaacgggtccggtaggc |
| ACTB TSS | actgcctggccactccatgc |
|  | catctcttgggcactgagcg |
| GAPDH TSS | gctccggctccaattcccca |
|  | tcggtgctggttcccaggac |
