# Supplementary material for: SUMOylation and ubiquitination reciprocally regulate SMCHD1 antiviral activity against herpes simplex virus 1
Source: PLoS Pathog. 2026 Jun 24;22(6):e1014371. doi: 10.1371/journal.ppat.1014371 (PMC13313348; doi:10.1371/journal.ppat.1014371)
Supplement: Attachment — Submitted filename: Response to Reviewers.pdf [file ppat.1014371.s011.pdf]

Additional Editor Comments:

Three experts have reviewed your manuscript and have provided detailed comments. All three recommended major revision. Please address the comments and include a point-by-point response with the revised manuscript. Thank you for submitting your work to PLOS Pathogens.

Response: We sincerely thank the Editor for the careful evaluation of our manuscript. We have addressed all comments raised by the reviewers and have provided a detailed, point-by-point response below. We believe that the manuscript has been substantially improved after revision.

Journal Requirements:

1) Please ensure that the CRediT author contributions listed for every co-author are completed accurately and in full.

At this stage, the following Authors/Authors require contributions: Xuezhang Tian, Xinyue Wang, Shaowei Wang, Yunhong Zhong, Yanlin Xia, Yang Chen, Ling He, Dongli Pan, Ke Lan, Zhengjun Shang, and Junjie Zhang. Please ensure that the full contributions of each author are acknowledged in the "Add/Edit/Remove Authors" section of our submission form.

The list of CRediT author contributions may be found here:
https://journals.plos.org/plospathogens/s/authorship#loc-author-contributions

2) We ask that a manuscript source file is provided at Revision. Please upload your manuscript file as a .doc, .docx, .rtf or .tex. If you are providing a .tex file, please upload it under the item type u2018LaTeX Source Fileu2019 and leave your .pdf version as the item type u2018Manuscriptu2019.

3) Please upload all main figures as separate Figure files in .tif or .eps format. For more information about how to convert and format your figure files please see our guidelines: https://journals.plos.org/plospathogens/s/figures

4) Please amend your detailed Financial Disclosure statement. This is published with the article. It must therefore be completed in full sentences and contain the exact wording you wish to be published.

State what role the funders took in the study. If the funders had no role in your study, please state: "The funders had no role in study design, data collection and analysis, decision to publish, or preparation of the manuscript.".

If you did not receive any funding for this study, please simply state: u201cThe authors received no specific funding for this work.u201d

Reviewers' Comments:

Reviewer's Responses to Questions

Part I - Summary

Please use this section to discuss strengths/weaknesses of study, novelty/significance, general execution and scholarship.

Reviewer #1: Virus-host interactions can dictate the outcome of viral infections. For herpes simplex virus 1 (HSV-1), it encounters host restriction factors during lytic infection and in several instances, the virus has developed countermeasures against these defenses. It was previously shown that the host factor, SMCHD1, a known restriction factor for other viruses, possess antiviral activity against HSV-1. In the current study, the authors sought to determine how SMCHD1 limits HSV-1 replication and if a viral factor counteracts SMCHD1's antiviral activity. The authors show that SMCHD1 restricts HSV-1 in at least 2 cell-types and requires SUMOylation of its hinge region for its inhibitory function, which is associated with binding to the HSV-1 genome to repress virus transcription. The authors go on to demonstrate that the HSV-1 E3 ubiquitin ligase, ICP0, binds to and promotes the degradation and ubiquitination of SMCHD1, leading to its inactivation. The ICP0-mediated degradation of SMCHD1 does not require its SUMOylation. Depletion or knockout of SMCHD1 can partially complement the replication of an ICP0 RING-finger or null mutant and to a lesser degree, WT HSV-1. The authors conclude that SUMOylation and ubiquitination differentially regulate SMCHD1's restrictive activity on HSV-1. The results presented and their interpretation were relatively straightforward and support most of the authors conclusions; however, specific experiments lacked quantitation or details, and the Discussion lacked a degree of in-depth analyses.

Response: We sincerely thank the reviewer for the positive and constructive comments. We have carefully addressed all concerns raised by the reviewer, including the addition of quantitative analyses, incorporation of the previously missing experimental details, and substantial revision of the Discussion to provide deeper mechanistic insight.

Reviewer #2: SUMOylation and ubiquitination reciprocally regulate SMCHD1 antiviral activity against herpes simplex virus 1

Manuscript ref. number: PPATHOGENS-D-26-00111

Xuezhang Tian, Xinyue Wang, Shaowei Wang, Yunhong Zhong, Yanlin Xia, Yang Chen, Ling He, Dongli Pan, Ke Lan, Zhengjun Shang and Junjie Zhang

Summary
The paper by Tian et al., builds on previous work from this group that previously identified SMCHD1 (structural maintenance of chromosomes flexible hinge domain-containing protein 1) to be a general host-cell restriction factor against a number of herpesviruses, including herpes simplex virus 1 (HSV-1) [DOI: 10.1128/mbio.00549-23]. Here, Tian and colleagues specifically focus on HSV-1 mediated restriction and mechanisms of viral counteraction and inactivation. The authors show SMCHD1 to restrict HSV-1 replication through its sub-cellular re-localization to viral DNA (vDNA) replication centres and interaction with HSV-1 vDNA in a SMCHD1 hinge-domain depedent manner, analogous to their findings for Kaposi's sarcoma associated virus (KSHV; DOI: 10.1128/mbio.00549-23). The authors to go on to show that HSV-1 counteracts this SMCHD1 restriction via the E3

ubiquitin ligase activity of the viral immediate (IE) protein ICP0, which mediates the SUMO-independent ubiquitination of SMCHD1 leading to its proteasomal degradation. In the absence of ICP0, infection with an HSV-1 ICP0-null mutant and/or catalytically inactive ICP0-RING mutant leads to the SUMOylation of SMCHD1. The authors propose SUMOylation of SMCHD1 is required to mediate HSV-1 restriction, positing a ubiquitination-SUMOylation axis in the regulation of SMCHD1 to regulate herpesvirus restriction. Overall, the quality of the data present is convincing, the standard of scientific writing high, and conclusions generally in line with their experimental observations. The authors should be congratulated on producing a very interesting and novel piece of science that could have wide readership appeal. I have listed my major and minor comments to this manuscript (MS) submission below.

Response: We sincerely thank the reviewer for the thoughtful and highly positive evaluation of our work. We have carefully considered all major and minor comments and performed additional experiments and revisions to address each point in detail, as outlined below.

Reviewer #3: In this manuscript, the authors focus on a novel restriction factor for HSV-1, SMCHD1, and have identified the mechanism through which the virus evades its antiviral function. SMCHD1 appears to bind the viral genome, in a sequence-independent manner, and impairs viral gene expression. The authors determined that sumoylated SMCHD1 restricts HSV-1. To counteract its effect HSV-1 ubiquitinates and degrades SMCHD1 in an ICP0-dependent manner.
The experimental design and the results are well done.
The study needs a few additions before publication listed below.

Response: We sincerely thank the reviewer for the positive evaluation of our study and for the constructive suggestions. We have carefully addressed all concerns raised and provide detailed responses to each point below.

Part II – Major Issues: Key Experiments Required for Acceptance
Please use this section to detail the key new experiments or modifications of existing experiments that should be absolutely required to validate study conclusions.

Generally, there should be no more than 3 such required experiments or major modifications for a "Major Revision" recommendation. If more than 3 experiments are necessary to validate the study conclusions, then you are encouraged to recommend "Reject".

Reviewer #1: 1. While Western blots indicated the relative quantitation of band intensities (e.g., Fig. 1A, 2A, C-F, Fig. 5A-F, Fig. 5D, Fig. 7, Fig. 8A-D), no statistical tests were provided to support their claims. Please include this information.
Answer: We thank the reviewer for the instructive comments. We have fully taken the

reviewer's suggestions.

In the revised manuscript, we have performed densitometric analyses based on at least three independent biological replicates for the indicated Western blot experiments, and statistical analyses have now been included (Fig. 1A, 2A-2F, 4A, 4C-4D, 4F, 7C-7D). Moreover, the Methods section has been revised to provide a detailed description of the densitometric quantification and statistical analyses.

Figure 1

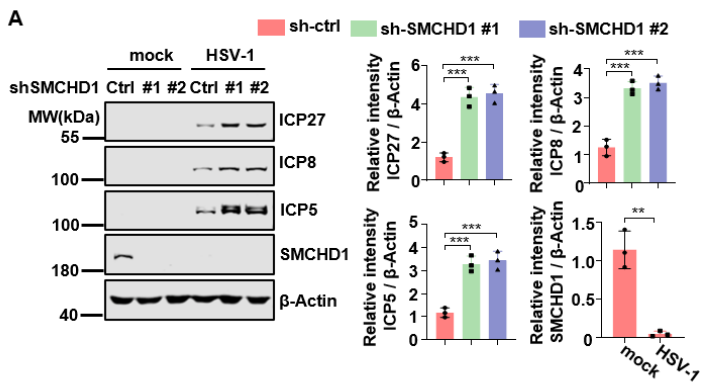

Figure 2

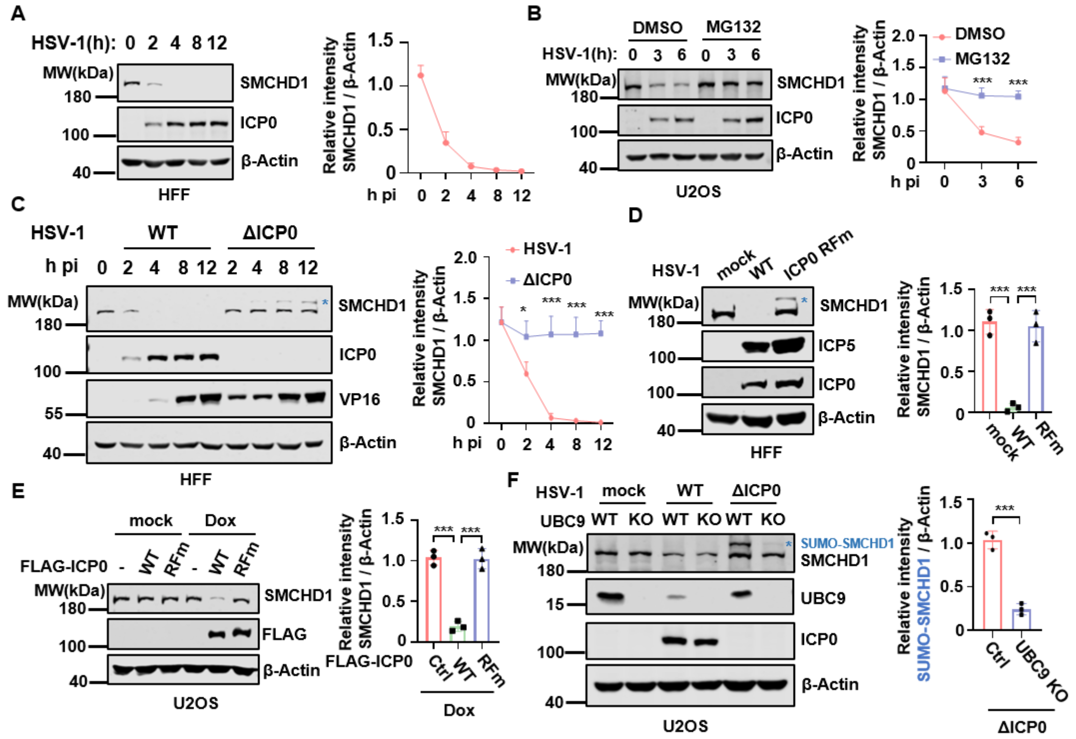

Figure 4

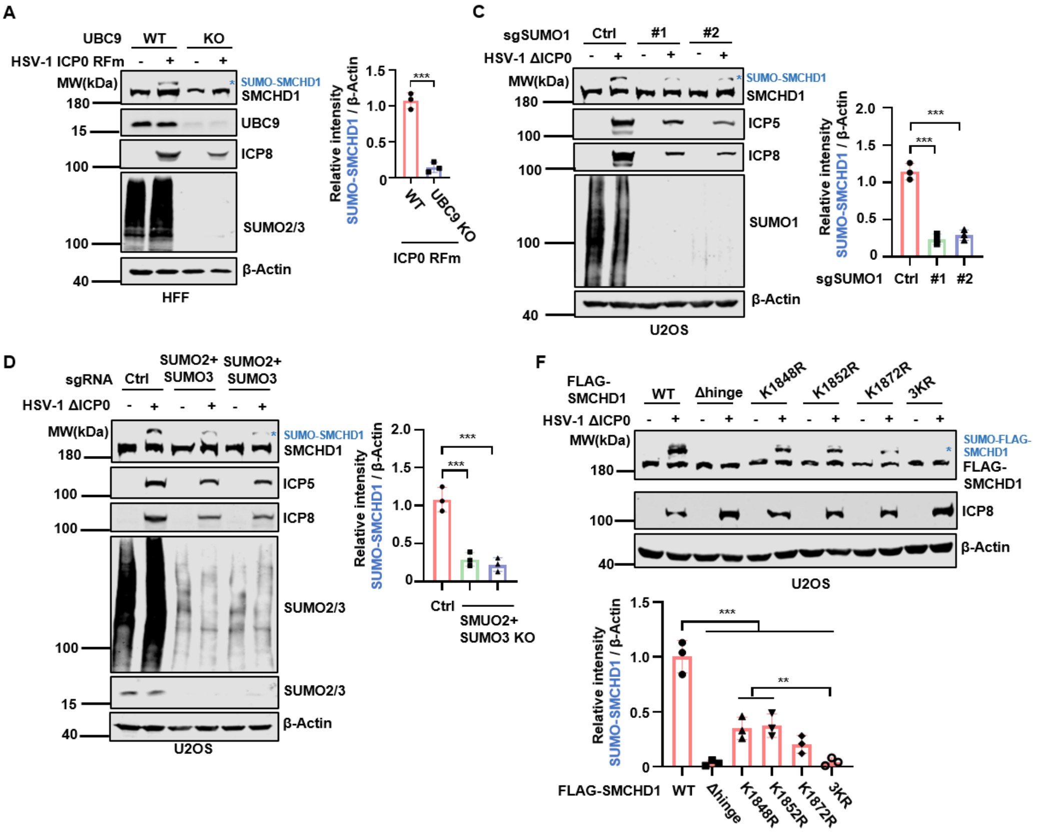

Figure 7

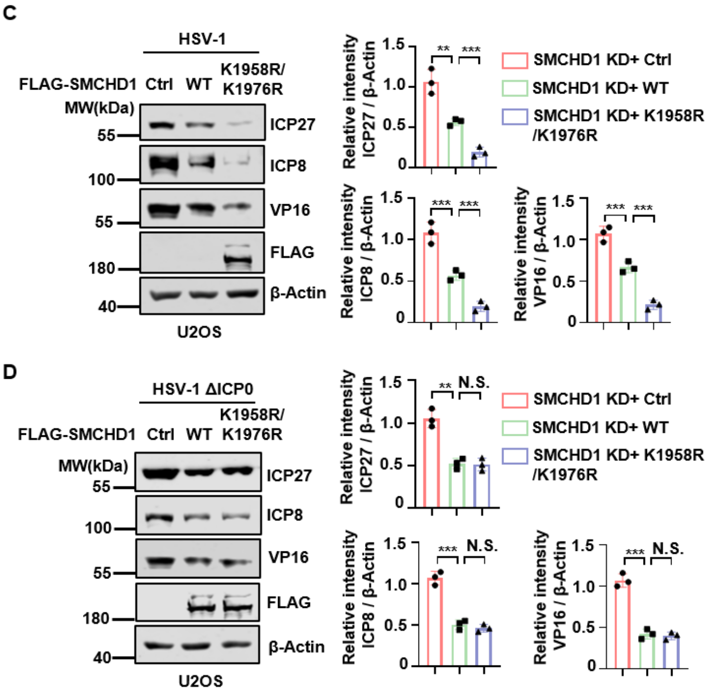

Reviewer #2: Major comments:

1. A significant proportion of the infection biology analysis conducted in this study is performed in U2OS cells, a cell line known to be permissive to HSV-1 ICP0-null mutant

infection (i.e., there is no functional requirement for the presence of ICP0 in these cells to stimulate the progress of HSV-1 infection). U2OS cells are known to be deficient or mutated in multiple host-cell factors which (in)directly regulate the outcome of HSV-1 infection (e.g., cGAS and ATRX). While the authors show similar (although not identical) infection data in human foreskin fibroblast cells to support their experimental observations derived in U2OS cells, much of this data is in supporting information. While U2OS cells can be extremely beneficial under certain experimental circumstances, there relevance to HSV-1 tropism, infection biology, and requirement for ICP0 to target host factors for degradation that actively mediate HSV-1 restriction is limited. The authors should revise the presentation of their data to be focused on human fibroblast cells (or equivalent restrictive cell type) in which there is a direct requirement for ICP0 as an accessory (non-essential) viral gene product to stimulate the progress of infection. As presented, the rationale for studying SMCHD1 in a cell line that doesn't require ICP0-mediated SMCHD1 degradation for infection to progress efficiently is confusing and undermines the interpretation of a substantial amount of their analysis. E.g., the authors show depletion of SMCHD1 in U2OS cells to lead to enhanced levels of IE gene transcription and viral replication (Figure 1). How do the authors reconcile this observation in a cell line that has been shown by multiple labs to be equally permissive to WT and ICP0-null mutant HSV-1? Notably, the authors appear to use multiple cell lines to titrate their viruses (line 659; 'Viral titers were quantified by standard plaque assays using VERO or U2OS cells25.'. As Vero cells are partially restrictive to ICP0-RFm and ICP0-null mutant HSV-1, which will skew the relative PFU/genome ratio of their titres, the authors should confirm that their infection experiments have been conducted at equivalent genome loads at the moi used throughout their study.

Answer: We sincerely thank the reviewer for the insightful comments.

We fully agree with the reviewer that U2OS cells are deficient or mutated in multiple host-cell factors that restrict HSV-1 replication. We originally selected U2OS cells because they support replication of HSV-1 ICP0-null and ICP0-RFm viruses more efficiently. Notably, even in U2OS cells, we consistently observed that WT HSV-1 replicated more efficiently than the ICP0-null mutant under our experimental conditions (revised Fig. S7D). This suggests that, although U2OS cells partially support replication of ICP0-deficient viruses, ICP0 still confers a replicative advantage, likely by counteracting residual restriction factors including SMCHD1. We therefore propose that SMCHD1 retains antiviral activity in U2OS cells and that ICP0-mediated degradation of SMCHD1 contributes to enhanced viral replication even in this partially permissive background.

Figure S7

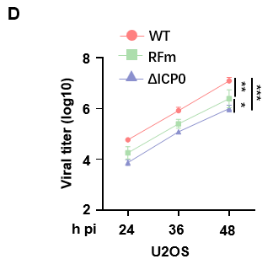

We agree with the reviewer that our presentation should emphasize data derived from restrictive cell types. Accordingly, we have reorganized the manuscript so that key

infection experiments are now presented using HFF cells in the main figures, while most U2OS-derived data have been moved to the Supplementary Information. In addition, we have performed several critical validation experiments in HFF cells (revised Fig. 1D-1E, 2C, 7A-7B) and incorporated these results into the main figures to strengthen the physiological relevance of our conclusions.

Figure 1

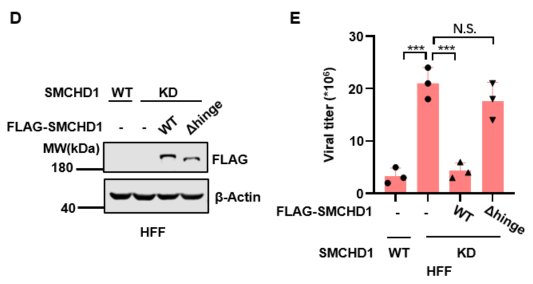

Figure 2

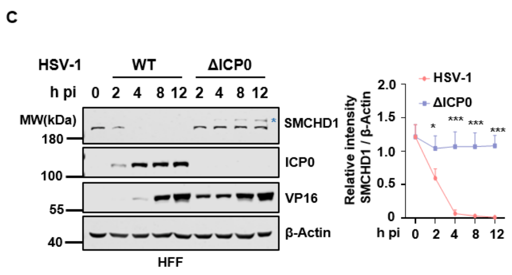

Figure 7

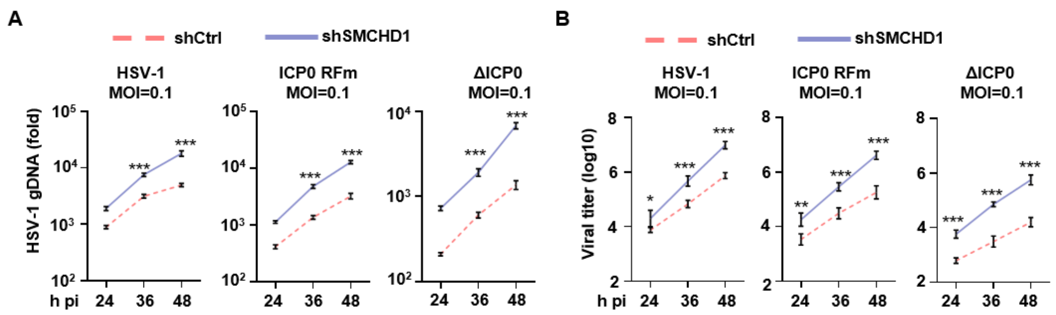

We also thank the reviewer for raising the important point regarding viral titration and normalization. Originally, viral titers of WT HSV-1 were determined using Vero cells, whereas ICP0-null and ICP0-RFm viruses were titrated in U2OS cells, given the known permissiveness of U2OS for ICP0-deficient viruses. To eliminate any potential ambiguity and ensure consistency, we have now re-titrated all viral strains using U2OS cells under identical conditions and have revised the Methods section accordingly.

Furthermore, to directly address the concern regarding potential skewing of the relative PFU/genome ratio, we quantified viral genome copy numbers and normalized infections across WT, ΔICP0, and ICP0-RFm viruses based on equivalent genome loads in the functional comparison experiments (Fig. 7 and Fig. S7). These additional analyses confirm that the observed differences in viral replication are not attributable to unequal viral input.

Figure S7

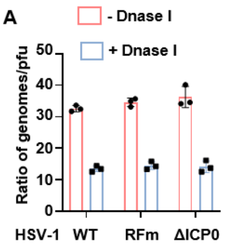

2. The authors should adjust the presentation order of their data currently presented in Figure 5 to match the narration in the text; i.e., move Fig. 5 to become Fig. 2. As presented, there is no clear rationale for investigating the SUMOylation of SMCHD1 prior to the discovery that in the absence of ICP0 during HSV-1 ICP0-null mutant infection induces its SUMOylation. This requires the reader to understand data present and described in Figure 5 first, prior to the current Figure 2.

Answer: We sincerely thank the reviewer for these insightful suggestions. We have fully taken the reviewer's suggestions.

To improve the clarity and logical flow of the manuscript, we have reorganized the figure order so that the data demonstrating ICP0-mediated degradation and ubiquitination of SMCHD1 are now presented earlier in the Results section. Specifically, the original Figure 5 has been moved to follow the initial characterization of SMCHD1 antiviral activity, thereby providing a clear mechanistic rationale for examining SUMOylation in the context of ICP0 deficiency.

3. A key conclusion in this study is the identification of SMCHD1 SUMOylation and the role this PTM plays in regulating the restriction of HSV-1. However, the rationale and data underlying SMCHD1 SUMOylation at K1848, K1852, and K1872 is inconclusive as currently presented. The PTMs for SMCHD1 have been extensively map, where multiple lysine residues have been identified to be ubiquitinated and/or SUMOylated (https://www.uniprot.org/uniprotkb/A6NHR9/entry). These data fail to identify K1848, K1852, and K1872 as acceptor sites for ubiquitin and/or SUMO modification. Moreover, these lysine residues do not comprise known consensus SUMO modification and/or SIM-motif sites. As mutation of these residues abolishes the ability of SMCHD1 to localise to vDNA, a function that occurs independently of its SUMO modification (Fig. 6C), the greater likelihood is that these residues mediate (directly or indirectly) vDNA interaction independently of SUMO-modification. Thus, the authors need to explicitly demonstrate that these residues are SUMO modified using mass spectrometry for their conclusions to stand. A loss of SUMOylation in their 3KR mutant could equally be explained by a failure to re-localize to sites at which SUMO modification occurs at alternative lysine residues that comprise known SUMO consensus motifs. While the data is clear that SMCHD1 is SUMOylated, the data as presented do not conclusively demonstrate the residues K1848, K1852, and K1872 are indeed the acceptor sites for this modification. Indeed, data present in Fig. S2 shows iteratively that transfection of flag-tagged SMCHD1 hinge domain mutant or 3KR mutant to undergo what appears to be SUMOylation. As this represents a major finding of the study, the authors need to conclusively demonstrate which residues are SUMOylated during ICP0-RFm or ICP0-null mutant HSV-1 infection and whether K1848, K1852, and K1872 are indeed SUMOylated and/or mediate vDNA binding independently of their respective SUMOylation status.

Answer: We sincerely thank the reviewer for the critical and insightful comments.

We agree with the reviewer that the identification of SMCHD1 SUMO acceptor sites at K1848, K1852, and K1872 would substantially strengthen the mechanistic conclusions of this study.

Following the reviewer's suggestions, we performed SUMO proteomics with or without

HSV-1 ICP0-RFm infection. We transfected SMCHD1, SUMO1-3, and UBC9 in 293T cells to facilitate the detection of SMCHD1 SUMOylation, followed by mock infection or HSV-1 ICP0-RFm infection. Proteins were extracted, digested, and subjected to immuno-affinity isolation of SUMOylated peptides prior to MS analysis (Lumpkin *et al.*, *Nat Commun, 2017*).

To rigorously address this point, we performed two independent SUMO proteomic analyses. These analyses identified more than 30 SUMOylated lysine residues within SMCHD1. However, we were unable to confidently detect SUMOylation at K1848, K1852, or K1872. In addition, most identified SUMOylation sites did not show substantial changes following HSV-1 ICP0-RFm infection, suggesting that these detected modifications likely represent basal SUMOylation events. Considering that SUMOylation is dynamically regulated and can be rapidly removed by deSUMOylating enzymes, the transient nature of SUMO conjugation and technical limitations in enrichment and MS detection sensitivity may restrict identification of infection-induced SUMOylation events. Regarding the reviewer's concern that mutation of these residues may impair vDNA interaction independently of SUMO modification, we performed two sets of experiments to address this possibility. First, to evaluate the functional significance of SUMOylation in viral DNA binding, we examined SMCHD1 association with the HSV-1 genome in UBC9 knockout cells. Deletion of UBC9 significantly reduced SMCHD1 binding to viral DNA (revised Fig. S6C), supporting the conclusion that SUMOylation contributes to SMCHD1 interaction with viral DNA.

Figure S6

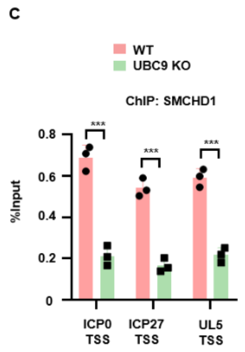

Second, we generated K1848R, K1852R, and K1872R single mutants to further examine the contribution of each residue. K1848R and K1852R did not alter SMCHD1 subcellular localization, whereas K1872R markedly affected SMCHD1 localization. Notably, all three single mutants exhibited reduced binding to viral DNA (revised Fig. S6E and S6F). In addition, our previous results (Figure 4F) indicate that K1848R, K1852R and K1872R each partially reduced HSV-1 infection-induced SMCHD1 SUMOylation, whereas the 3KR mutant almost completely abolished infection-induced SUMOylation. Importantly, because K1848R and K1852R did not alter SMCHD1 localization, the reduced viral DNA binding observed for these mutants is unlikely to result from localization defects. Together, these findings support that K1848 and K1852 are functionally linked to HSV-1 infection-induced SUMOylation and viral DNA binding, although direct MS evidence for modification at these residues is currently lacking.

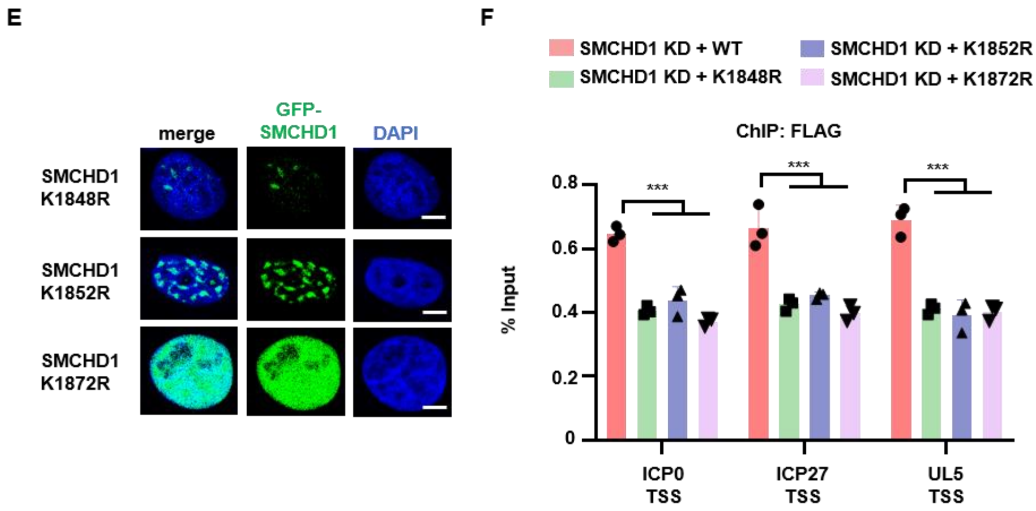

For K1872, because the mutation substantially alters SMCHD1 localization, we cannot exclude the possibility that the observed effects on SUMOylation and viral DNA binding are partially secondary to altered subcellular localization.

Accordingly, we have revised the manuscript to avoid overinterpretation and no longer conclude that K1848, K1852, and K1872 represent definitive HSV-1-induced SUMO acceptor sites.

Although K1848 and K1852 do not conform to the canonical ΨKxE consensus motif, previous proteomic studies have revealed that a substantial fraction of SUMOylation events occur at non-consensus sites (Impens *et al.*, *PNAS*, 2014; Xiao *et al.*, *Reproduction,* 2016). We have revised the Discussion section to include this point.

With respect to the residual SUMOylation observed in Fig. S2, our MS analysis also supports that SMCHD1 undergoes basal SUMOylation at additional lysine residues under steady-state conditions. Consistently, we performed new experiments and observed weak basal band shifts of SMCHD1, whereas SUMOylation was markedly enhanced upon co-expression of UBC9 and SUMO1-3 (revised Fig. S4C).

Figure S4

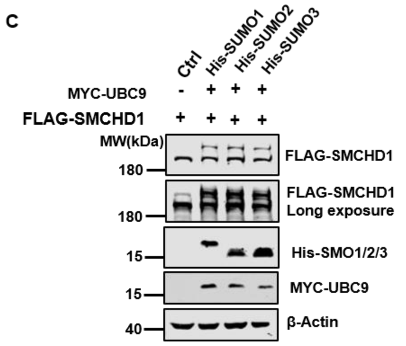

In summary, our current data support that SMCHD1 undergoes SUMOylation during HSV-1 infection and that SUMOylation contributes to viral DNA binding and antiviral restriction. However, we acknowledge that direct MS evidence demonstrating K1848, K1852 and K1872 as definitive infection-induced SUMO acceptor sites is currently lacking. Accordingly, we have revised the manuscript to avoid overinterpreting these residues as definitively identified SUMOylation sites during HSV-1 infection. Instead, we

now conclude that these residues are functionally linked to infection-induced SUMOylation and viral DNA binding.

Reviewer #3: N/A

Part III – Minor Issues: Editorial and Data Presentation Modifications

Please use this section for editorial suggestions as well as relatively minor modifications of existing data that would enhance clarity.

Reviewer #1: 1. The authors did not provide the degree of relative (and significant) decreases or increases (e.g., 5-fold) for most experiments described in the Results section. Furthermore, figure legends do not indicate the sample number and the number of times each experiment was repeated. Please include such information.

Answer: We thank the reviewer for the instructive comments.

We have revised the manuscript accordingly. The degree of relative changes is now included in the Results section where appropriate. In addition, all figure legends have been updated to indicate the sample size (n), the number of independent experiments performed, and the statistical analyses used. The Methods section has also been revised to provide a detailed description of experimental replication and statistical procedures.

2. The authors use U2OS cells, a cell type known to extensively or fully complement the replication of ICP0 mutants (null and RING-finger) based on several published reports. With this background, why do the authors observe reductions in the replication of ICP0 mutants relative to WT HSV-1 in U2OS cells (i.e., Fig. 7B, shCtrl)? Please explain.

Answer: We thank the reviewer for this critical point, which was also raised by reviewer #2.

We originally selected U2OS cells because they support replication of HSV-1 ICP0-null and ICP0-RFm viruses more efficiently. Although U2OS cells have been reported to extensively complement ICP0-null replication, under our experimental conditions we consistently observed that WT HSV-1 replicated more efficiently than ICP0-null or ICP0-RFm viruses in U2OS cells (revised Fig. S7D). This suggests that, although U2OS cells partially support replication of ICP0-deficient viruses, ICP0 still confers a measurable replicative advantage, likely by counteracting residual restriction factors, including SMCHD1. Consistently, depletion of SMCHD1 enhances viral replication in U2OS cells, supporting the conclusion that SMCHD1 retains antiviral activity in this cellular background.

To improve clarity and strengthen the physiological relevance of the study, we have reorganized the manuscript to emphasize data obtained in HFF cells in the main figures and have clarified the rationale for the use of U2OS cells in the revised Discussion section.

Figure S7

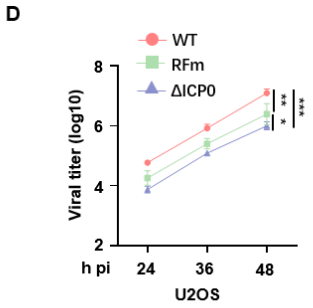

3. Description of Western blot procedures, densitometric analyses, the GST-ICP0 (1-241) construct for in vitro ubiquitination, and the amount of GST or GST-ICP0 used in this in vitro ubiquitination assay are not described in the Materials and Methods. Please incorporate these details in this subsection of the manuscript.

Answer: We thank the reviewer for the instructive comments.

We have revised the Materials and Methods section to include detailed descriptions of the Western blot procedures and densitometric analyses. In addition, we have provided information regarding the GST-ICP0 (1–241) construct and specify the amounts of GST and GST-ICP0 used in the in vitro ubiquitination assays.

4. The Discussion in its current form is not fully developed. There are several points to consider. Does SMCHD1 restrict HIV-1 and other viruses in a manner similar to HSV-1 (e.g., by binding to nucleic acid). What other cellular proteins does SMCHD1 bind to, allowing it to be an epigenetic repressor? How does SMCHD1 SUMOylation enhance its binding to HSV-1 DNA? What is the authors' model (in schematic form) based on their data? Answers to these questions will significantly strengthen the Discussion.

Answer: We thank the reviewer for these constructive suggestions.

In the revised manuscript, we have substantially expanded the Discussion to address the broader antiviral roles of SMCHD1. We discuss the possibility that SMCHD1 may restrict HIV-1 and other viruses through nucleic acid-associated mechanisms, although this remains to be directly tested. After HIV enters host cells, its RNA genome is reverse transcribed into DNA, raising the possibility that chromatin-associated restriction mechanisms may contribute to the control of viral transcription. Consistent with this, SMCHD1 has been shown to form a complex with LRIF1 and HP1 that associates with the AAV genome, maintaining it in a heterochromatic state and repressing transcription (Wang et al., *PLoS pathogens*, 2024). These findings suggest that SMCHD1 could potentially target viral DNA to limit viral transcription.

We have also discussed potential mechanisms by which SUMOylation enhances SMCHD1 recruitment to HSV-1 DNA and promotes transcriptional silencing. In addition, we have incorporated a schematic model summarizing our proposed mechanism based on the experimental data presented in this study (revised Fig. 7G). We believe these revisions significantly strengthen the conceptual framework of the study.

G

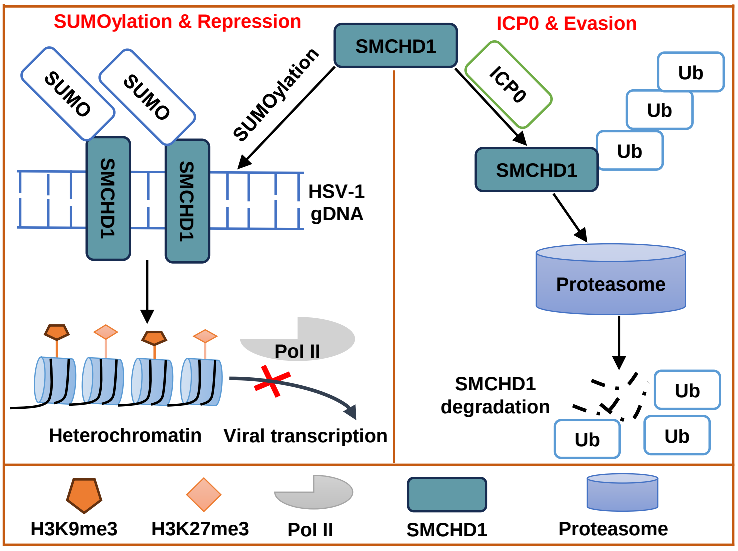

5. Information provided for many references are incomplete. Please recheck the references and ensure that their details are complete.

Answer: We thank the reviewer for pointing out this issue. We have carefully corrected the reference list, and all citations have been updated to ensure complete and accurate bibliographic information.

Reviewer #2: Minor comments:

1. The authors lay summary reads almost identically to that of the abstract. I would strongly encourage the authors to re-write this lay summary to increase the readership appeal of the MS to the broad readership of PLoS Pathogens.

Answer:

We thank the reviewer for this instructive comment. We have rewritten the lay summary to improve its clarity, accessibility, and appeal to a broad readership, ensuring that it is clearly distinct from the abstract and more effectively highlights the biological significance and broader implications of our findings.

2. The introduction lists known HSV-1 restriction factors (e.g., MxB, TRIM22) but fails to mention other chromatin-modifiers or binding proteins (e.g., Daxx, ATRX, HIRA) known to restrict HSV-1 infection that are counteracted (degraded or displaced) by the E3 ubiquitin ligase activity of ICP0. The authors should revise their introduction and discussion accordingly to reflect the potential role of SMCHD1 to mediate chromatin silencing (Fig. S4).

Answer:

We thank the reviewer for this critical suggestion. We have revised the Introduction and Discussion sections to include additional chromatin-associated restriction factors, such as Daxx, ATRX, and HIRA, which are known to mediate epigenetic silencing of HSV-1 genomes and are targeted by ICP0.

We have further discussed the potential role of SMCHD1 as a chromatin-associated repressor in this context and its possible functional parallels with these intrinsic immunity factors (lines 58-65, lines 397-405). These additions provide a clearer conceptual

framework linking SMCHD1-mediated restriction to established chromatin-based antiviral defense mechanisms.

3. Line 124; 'In contrast, HSV-1 ΔICP0 replication is severely impaired in HFF and HEK293T cells, whereas HSV-1 ICP0 RFm replicates substantially more efficiently in these cell types 30, 31.' This statement is confusing. There have been a substantial number of studies demonstrating the RING-finger (RF) to be essential for ICP0 E3 ubiquitin ligase activity and biological function to stimulate the progress of infection (e.g., PMID: 22278229). The authors should provide additional supporting data for this statement to stand for their KOS strain derived HSV-1 ICP0 RFm.

Answer:

We thank the reviewer for this critical comment. We agree that the RING-finger domain is well established as essential for ICP0 E3 ubiquitin ligase activity and for efficient progression of HSV-1 infection.

To directly address this concern, we have now performed multi-step growth curve analyses. Specifically, we compared WT, ICP0-RFm and ΔICP0 viruses derived from the KOS strain in HFF and HEK293T cells. These data indicate that, while both ΔICP0 and ICP0-RFm viruses exhibit significantly impaired replication compared to WT viruses, ICP0-RFm displays partial replication competence, whereas ΔICP0 shows more severe attenuation. These results are now presented in revised Fig. S4C.

Figure S4

B

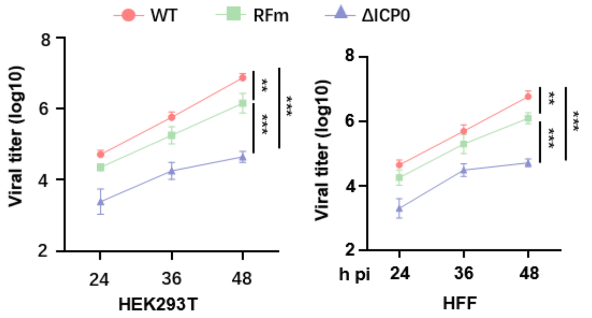

Consistent with the reviewer's point, our data confirm that the RING-finger domain is required for full ICP0 function, as RFm replication remains markedly reduced compared to WT virus. However, the observed difference between ΔICP0 and RFm viruses in our system likely reflects residual, RING-independent functions of ICP0 or cell-type-specific effects. We recognize that the original phrasing may have led to ambiguity and have revised the text for greater clarity and precision.

4. line 240; 'Because ICP0 is a putative RING-type E3 ubiquitin,⋯.'. This statement if inaccurate and should be revised. There are an abundance of studies demonstrating ICP0 to be a RING-finger E3 ubiquitin ligase that can interact with multiple host-cell factors to mediate their ubiquitination.

Answer: We thank the reviewer for this critical comment. We fully agree with the reviewer's point.

We have revised the text to describe ICP0 as a well-established RING-finger E3 ubiquitin ligase and have updated the Introduction and Discussion to include appropriate references supporting ICP0-mediated ubiquitination of multiple host factors.

5. As the interaction between ICP0 and SMCHD1 occurs within the first 100aa of the N-terminus of ICP0, which predominantly includes the CK1 ICP0-T67 phosphorylation site required for RNF8 FHA domain interaction. The authors should investigate, or at least discuss, the potential role for RNF8 and/or RNF168 to mediate the (co)degradation of SMCHD1 as a component of the DDR response that utilises host SUMOylation for recruitment of host factosr to sites of DNA damage.

Answer:

We thank the reviewer for this important suggestion.

To directly address the potential involvement of RNF8, we performed two sets of experiments. First, RNF8 or RNF168 knockout did not impair ICP0-mediated SMCHD1 degradation (Fig. S3B). Second, we tested an ICP0-T67A mutant that disrupts RNF8 recruitment. Although ICP0-T67A failed to recruit RNF8, it retained the ability to promote SMCHD1 ubiquitination and degradation (Fig. S3C). Together, these results indicate that RNF8 recruitment is not required for ICP0-induced SMCHD1 degradation. We have incorporated these data into the revised manuscript.

Figure S3

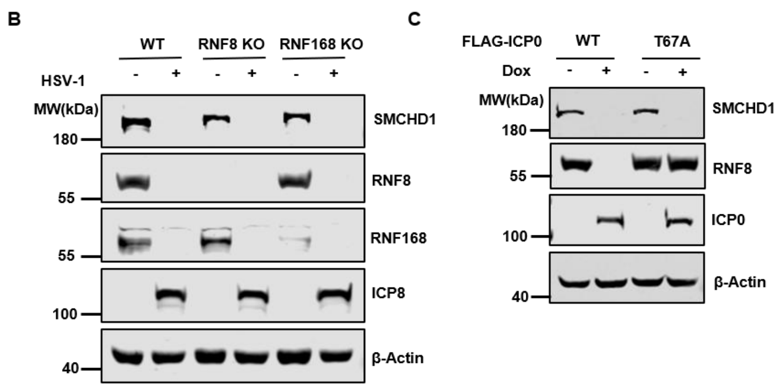

Reviewer #3: - The authors need to include a kinetic with the WT virus to indicate when SMCHD1 is actually degraded.

Answer:

We thank the reviewer for this instructive comment.

We have performed a time-course analysis during WT HSV-1 infection. The results indicate that SMCHD1 degradation begins at approximately 2 hours post-infection and is nearly complete by 4 hours post-infection (revised Fig. 2A and Fig. S2A).

Figure 2

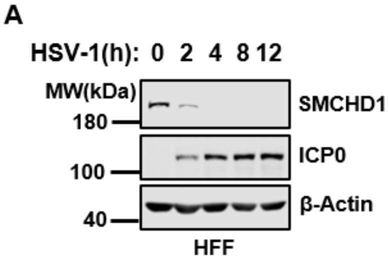

Figure S2

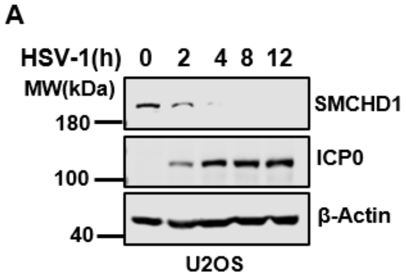

- Also, the authors need to include a kinetic with ΔICP0 both in U2OS and HFF to determine if degradation of SMCHD1 is observed later.

Answer:

We thank the reviewer for the instructive comments.

We have performed time-course analyses of SMCHD1 protein levels during ΔICP0 infection in both U2OS and HFF cells. In contrast to WT infection, SMCHD1 degradation was not observed at any of the examined time points in either cell type, including up to 12 hours post-infection (revised Fig. 2C and Fig. S2D). These data have been incorporated into the revised manuscript to further clarify the ICP0 dependence of SMCHD1 degradation.

Figure 2

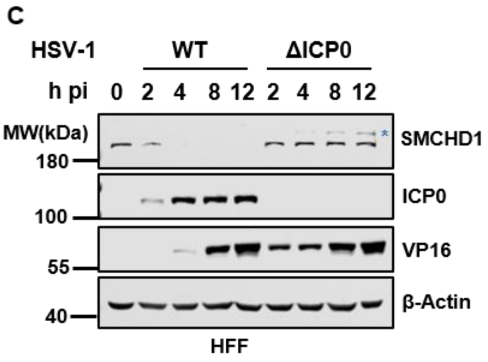

Figure S2

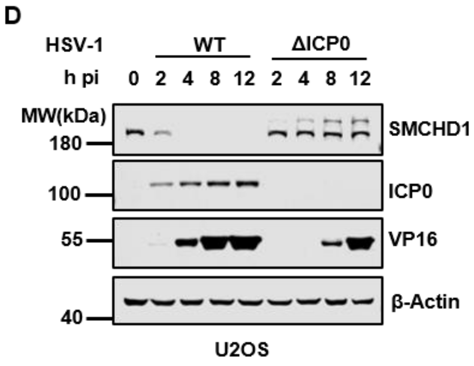

- The authors need to test colocalization of ICP0 with SMCHD1 at the ND10 bodies.

Answer:

We thank the reviewer for the instructive comments.

To address the reviewer's comment, we performed immunofluorescence assays to examine the subcellular localization of ICP0, SMCHD1, and PML during the early phase of HSV-1 infection. Our results demonstrate that upon HSV-1 infection, ICP0, SMCHD1, and PML exhibit clear colocalization (revised Fig. S3A). In contrast, in uninfected cells, PML and SMCHD1 do not show any detectable colocalization (revised Fig. S3A).

Figure S3

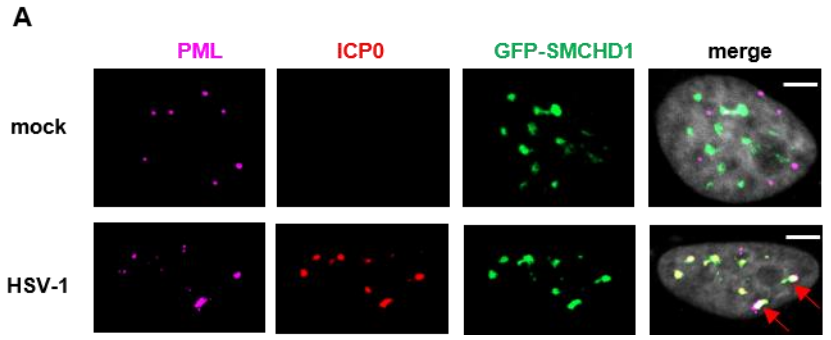

- In Fig. 7 authors need to provide growth curves and not a single time point for virus growth.

Answer:

We thank the reviewer for the instructive comments.

We have performed growth curve analyses for the indicated viruses. Viral titers were measured at multiple time points post-infection to assess replication kinetics. The results confirm our original conclusions and are now presented in revised Fig. 7A, 7B and Fig. S7B, S7C

Figure 7

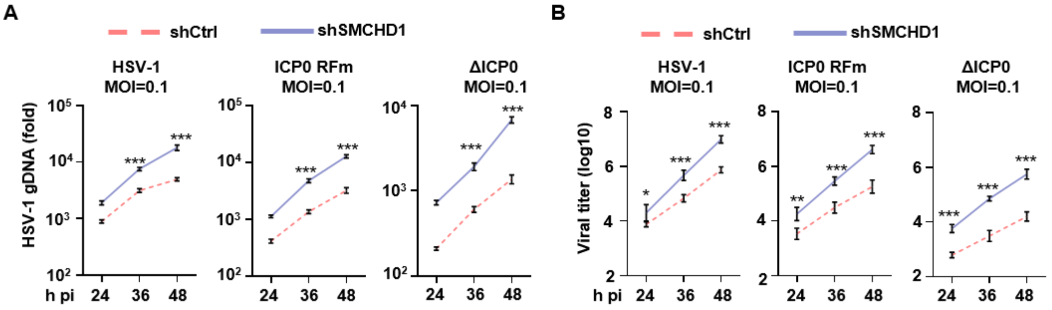

Figure S7

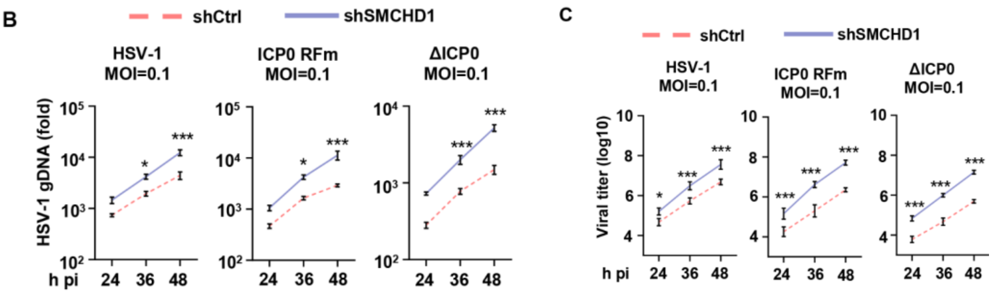

- In U2OS cells, HSV-1 and ΔICP0 generally grow to comparable levels, even though ΔICP0 cannot degrade SMCHD1. The authors need to provide growth curves of the 2 viruses in U2OS.

Answer:

We thank the reviewer for the instructive comment.

We agree that U2OS cells have been reported to substantially complement ICP0 deficiency. To directly address this concern, we have now performed multi-step growth

curve analyses comparing WT and ΔICP0 viruses in U2OS cells. Under our experimental conditions, ΔICP0 replication was consistently reduced relative to WT virus, although the magnitude of the defect was less pronounced than that observed in restrictive cell types (revised Fig. S7B). These data have now been incorporated into the revised manuscript to more comprehensively document viral growth kinetics in U2OS cells.

Figure S7

D

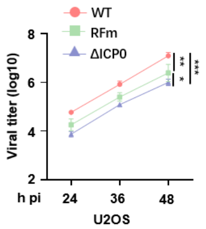

- Introduction and discussion are very similar.

Answer:

We thank the reviewer for this insightful comment.

We have revised the manuscript to clearly differentiate the Introduction and Discussion sections. The Introduction has been streamlined to focus on background and rationale, whereas the Discussion has been substantially rewritten to emphasize interpretation of our findings, mechanistic insights, and their broader implications. The revised manuscript now presents a clearer distinction between these sections.
