# Supplementary material for: SUMOylation and ubiquitination reciprocally regulate SMCHD1 antiviral activity against herpes simplex virus 1
Source: PLoS Pathog. 2026 Jun 24;22(6):e1014371. doi: 10.1371/journal.ppat.1014371 (PMC13313348; doi:10.1371/journal.ppat.1014371)
Supplement: Peer review history

# Peer review: Decision Letter 0

**DOI:** 10.1371/journal.ppat.1014371.r001

**Authors:** Robert Kalejta, Anthony Nicola

17 Feb 2026

-->PPATHOGENS-D-26-00111

SUMOylation and ubiquitination reciprocally regulate SMCHD1 antiviral activity against herpes simplex virus 1

PLOS Pathogens

Dear Dr. Zhang,

Thank you for submitting your manuscript to PLOS Pathogens. After careful consideration, we feel that it has merit but does not fully meet PLOS Pathogens's publication criteria as it currently stands. Therefore, we invite you to submit a revised version of the manuscript that addresses the points raised during the review process.

Please submit your revised manuscript by Apr 18 2026 11:59PM. If you will need more time than this to complete your revisions, please reply to this message or contact the journal office at plospathogens@plos.org. When you're ready to submit your revision, log on to https://www.editorialmanager.com/ppathogens/ and select the 'Submissions Needing Revision' folder to locate your manuscript file.

Please include the following items when submitting your revised manuscript:

* A letter that responds to each point raised by the editor and reviewer(s). You should upload this letter as a separate file labeled 'Response to Reviewers'. This file does not need to include responses to any formatting updates and technical items listed in the 'Journal Requirements' section below.

* A marked-up copy of your manuscript that highlights changes made to the original version. You should upload this as a separate file labeled 'Revised Manuscript with Track Changes'.

* An unmarked version of your revised paper without tracked changes. You should upload this as a separate file labeled 'Manuscript'.

If you would like to make changes to your financial disclosure, competing interests statement, or data availability statement, please make these updates within the submission form at the time of resubmission. Guidelines for resubmitting your figure files are available below the reviewer comments at the end of this letter.

We look forward to receiving your revised manuscript.

Kind regards,

Anthony Nicola

Academic Editor

PLOS Pathogens

Robert Kalejta

Section Editor

PLOS Pathogens

Sumita Bhaduri-McIntosh

Editor-in-Chief

PLOS Pathogens

orcid.org/0000-0003-2946-9497

Michael Malim

Editor-in-Chief

PLOS Pathogens

orcid.org/0000-0002-7699-2064

**Additional Editor Comments:**

Three experts have reviewed your manuscript and have provided detailed comments. All three recommended major revision. Please address the comments and include a point-by-point response with the revised manuscript. Thank you for submitting your work to PLOS Pathogens.

**Journal Requirements:**

1) Please ensure that the CRediT author contributions listed for every co-author are completed accurately and in full.

At this stage, the following Authors/Authors require contributions: Xuezhang Tian, Xinyue Wang, Shaowei Wang, Yunhong Zhong, Yanlin Xia, Yang Chen, Ling He, Dongli Pan, Ke Lan, Zhengjun Shang, and Junjie Zhang. Please ensure that the full contributions of each author are acknowledged in the "Add/Edit/Remove Authors" section of our submission form.

The list of CRediT author contributions may be found here: https://journals.plos.org/plospathogens/s/authorship#loc-author-contributions

2) We ask that a manuscript source file is provided at Revision. Please upload your manuscript file as a .doc, .docx, .rtf or .tex. If you are providing a .tex file, please upload it under the item type u2018LaTeX Source Fileu2019 and leave your .pdf version as the item type u2018Manuscriptu2019.

3) Please upload all main figures as separate Figure files in .tif or .eps format. For more information about how to convert and format your figure files please see our guidelines:

https://journals.plos.org/plospathogens/s/figures

4) Please amend your detailed Financial Disclosure statement. This is published with the article. It must therefore be completed in full sentences and contain the exact wording you wish to be published.

State what role the funders took in the study. If the funders had no role in your study, please state: "The funders had no role in study design, data collection and analysis, decision to publish, or preparation of the manuscript.".

If you did not receive any funding for this study, please simply state: u201cThe authors received no specific funding for this work.u201d

**Reviewers' Comments:**

Reviewer's Responses to Questions

**Part I - Summary**

Please use this section to discuss strengths/weaknesses of study, novelty/significance, general execution and scholarship.

Reviewer #1: Virus-host interactions can dictate the outcome of viral infections. For herpes simplex virus 1 (HSV-1), it encounters host restriction factors during lytic infection and in several instances, the virus has developed countermeasures against these defenses. It was previously shown that the host factor, SMCHD1, a known restriction factor for other viruses, possess antiviral activity against HSV-1. In the current study, the authors sought to determine how SMCHD1 limits HSV-1 replication and if a viral factor counteracts SMCHD1’s antiviral activity. The authors show that SMCHD1 restricts HSV-1 in at least 2 cell-types and requires SUMOylation of its hinge region for its inhibitory function, which is associated with binding to the HSV-1 genome to repress virus transcription. The authors go on to demonstrate that the HSV-1 E3 ubiquitin ligase, ICP0, binds to and promotes the degradation and ubiquitination of SMCHD1, leading to its inactivation. The ICP0-mediated degradation of SMCHD1 does not require its SUMOylation. Depletion or knockout of SMCHD1 can partially complement the replication of an ICP0 RING-finger or null mutant and to a lesser degree, WT HSV-1. The authors conclude that SUMOylation and ubiquitination differentially regulate SMCHD1’s restrictive activity on HSV-1. The results presented and their interpretation were relatively straightforward and support most of the authors conclusions; however, specific experiments lacked quantitation or details, and the Discussion lacked a degree of in-depth analyses.

Reviewer #2: SUMOylation and ubiquitination reciprocally regulate SMCHD1 antiviral activity against herpes simplex virus 1

Manuscript ref. number: PPATHOGENS-D-26-00111

Xuezhang Tian, Xinyue Wang, Shaowei Wang, Yunhong Zhong, Yanlin Xia, Yang Chen, Ling He, Dongli Pan, Ke Lan, Zhengjun Shang and Junjie Zhang

Summary

The paper by Tian et al., builds on previous work from this group that previously identified SMCHD1 (structural maintenance of chromosomes flexible hinge domain-containing protein 1 ) to be a general host-cell restriction factor against a number of herpesviruses, including herpes simplex virus 1 (HSV-1) [DOI: 10.1128/mbio.00549-23]. Here, Tian and colleagues specifically focus on HSV-1 mediated restriction and mechanisms of viral counteraction and inactivation. The authors show SMCHD1 to restrict HSV-1 replication through its sub-cellular re-localization to viral DNA (vDNA) replication centres and interaction with HSV-1 vDNA in a SMCHD1 hinge-domain depedent manner, analogous to their findings for Kaposi’s sarcoma associated virus (KSHV; DOI: 10.1128/mbio.00549-23). The authors to go on to show that HSV-1 counteracts this SMCHD1 restriction via the E3 ubiquitin ligase activity of the viral immediate (IE) protein ICP0, which mediates the SUMO-independent ubiquitination of SMCHD1 leading to its proteasomal degradation. In the absence of ICP0, infection with an HSV-1 ICP0-null mutant and/or catalytically inactive ICP0-RING mutant leads to the SUMOylation of SMCHD1. The authors propose SUMOylation of SMCHD1 is required to mediate HSV-1 restriction, positing a ubiquitination-SUMOylation axis in the regulation of SMCHD1 to regulate herpesvirus restriction. Overall, the quality of the data present is convincing, the standard of scientific writing high, and conclusions generally in line with their experimental observations. The authors should be congratulated on producing a very interesting and novel piece of science that could have wide readership appeal. I have listed my major and minor comments to this manuscript (MS) submission below.

Reviewer #3: In this manuscript, the authors focus on a novel restriction factor for HSV-1, SMCHD1, and have identified the mechanism through which the virus evades its antiviral function. SMCHD1 appears to bind the viral genome, in a sequence-independent manner, and impairs viral gene expression. The authors determined that sumoylated SMCHD1 restricts HSV-1. To counteract its effect HSV-1 ubiquitinates and degrades SMCHD1 in an ICP0-dependent manner.

The experimental design and the results are well done.

The study needs a few additions before publication listed below.

**********

**Part II – Major Issues: Key Experiments Required for Acceptance**

Please use this section to detail the key new experiments or modifications of existing experiments that should be absolutely required to validate study conclusions.

Generally, there should be no more than 3 such required experiments or major modifications for a "Major Revision" recommendation. If more than 3 experiments are necessary to validate the study conclusions, then you are encouraged to recommend "Reject".

Reviewer #1: 1. While Western blots indicated the relative quantitation of band intensities (e.g., Fig. 1A, 2A, C-F, Fig. 5A-F, Fig. 5D, Fig. 7, Fig. 8A-D), no statistical tests were provided to support their claims. Please include this information.

Reviewer #2: Major comments:

1. A significant proportion of the infection biology analysis conducted in this study is performed in U2OS cells, a cell line known to be permissive to HSV-1 ICP0-null mutant infection (i.e., there is no functional requirement for the presence of ICP0 in these cells to stimulate the progress of HSV-1 infection). U2OS cells are known to be deficient or mutated in multiple host-cell factors which (in)directly regulate the outcome of HSV-1 infection (e.g., cGAS and ATRX). While the authors show similar (although not identical) infection data in human foreskin fibroblast cells to support their experimental observations derived in U2OS cells, much of this data is in supporting information. While U2OS cells can be extremely beneficial under certain experimental circumstances, there relevance to HSV-1 tropism, infection biology, and requirement for ICP0 to target host factors for degradation that actively mediate HSV-1 restriction is limited. The authors should revise the presentation of their data to be focused on human fibroblast cells (or equivalent restrictive cell type) in which there is a direct requirement for ICP0 as an accessory (non-essential) viral gene product to stimulate the progress of infection. As presented, the rationale for studying SMCHD1 in a cell line that doesn’t require ICP0-mediated SMCHD1 degradation for infection to progress efficiently is confusing and undermines the interpretation of a substantial amount of their analysis. E.g., the authors show depletion of SMCHD1 in U2OS cells to lead to enhanced levels of IE gene transcription and viral replication (Figure 1). How do the authors reconcile this observation in a cell line that has been shown by multiple labs to be equally permissive to WT and ICP0-null mutant HSV-1? Notably, the authors appear to use multiple cell lines to titrate their viruses (line 659; ‘Viral titers were quantified by standard plaque assays using VERO or U2OS cells25.’. As Vero cells are partially restrictive to ICP0-RFm and ICP0-null mutant HSV-1, which will skew the relative PFU/genome ratio of their titres, the authors should confirm that their infection experiments have been conducted at equivalent genome loads at the moi used throughout their study.

2. The authors should adjust the presentation order of their data currently presented in Figure 5 to match the narration in the text; i.e., move Fig. 5 to become Fig. 2. As presented, there is no clear rationale for investigating the SUMOylation of SMCHD1 prior to the discovery that in the absence of ICP0 during HSV-1 ICP0-null mutant infection induces its SUMOylation. This requires the reader to understand data present and described in Figure 5 first, prior to the current Figure 2.

3. A key conclusion in this study is the identification of SMCHD1 SUMOylation and the role this PTM plays in regulating the restriction of HSV-1. However, the rationale and data underlying SMCHD1 SUMOylation at K1848, K1852, and K1872 is inconclusive as currently presented. The PTMs for SMCHD1 have been extensively map, where multiple lysine residues have been identified to be ubiquitinated and/or SUMOylated (https://www.uniprot.org/uniprotkb/A6NHR9/entry). These data fail to identify K1848, K1852, and K1872 as acceptor sites for ubiquitin and/or SUMO modification. Moreover, these lysine residues do not comprise known consensus SUMO modification and/or SIM-motif sites. As mutation of these residues abolishes the ability of SMCHD1 to localise to vDNA, a function that occurs independently of its SUMO modification (Fig. 6C), the greater likelihood is that these residues mediate (directly or indirectly) vDNA interaction independently of SUMO-modification. Thus, the authors need to explicitly demonstrate that these residues are SUMO modified using mass spectrometry for their conclusions to stand. A loss of SUMOylation in their 3KR mutant could equally be explained by a failure to re-localize to sites at which SUMO modification occurs at alternative lysine residues that comprise known SUMO consensus motifs. While the data is clear that SMCHD1 is SUMOylated, the data as presented do not conclusively demonstrate the residues K1848, K1852, and K1872 are indeed the acceptor sites for this modification. Indeed, data present in Fig. S2 shows iteratively that transfection of flag-tagged SMCHD1 hinge domain mutant or 3KR mutant to undergo what appears to be SUMOylation. As this represents a major finding of the study, the authors need to conclusively demonstrate which residues are SUMOylated during ICP0-RFm or ICP0-null mutant HSV-1 infection and whether K1848, K1852, and K1872 are indeed SUMOylated and/or mediate vDNA binding independently of their respective SUMOylation status.

Reviewer #3: N/A

**********

**Part III – Minor Issues: Editorial and Data Presentation Modifications**

Please use this section for editorial suggestions as well as relatively minor modifications of existing data that would enhance clarity.

Reviewer #1: 1. The authors did not provide the degree of relative (and significant) decreases or increases (e.g., 5-fold) for most experiments described in the Results section. Furthermore, figure legends do not indicate the sample number and the number of times each experiment was repeated. Please include such information.

2. The authors use U2OS cells, a cell type known to extensively or fully complement the replication of ICP0 mutants (null and RING-finger) based on several published reports. With this background, why do the authors observe reductions in the replication of ICP0 mutants relative to WT HSV-1 in U2OS cells (i.e., Fig. 7B, shCtrl)? Please explain.

3. Description of Western blot procedures, densitometric analyses, the GST-ICP0 (1-241) construct for in vitro ubiquitination, and the amount of GST or GST-ICP0 used in this in vitro ubiquitination assay are not described in the Materials and Methods. Please incorporate these details in this subsection of the manuscript.

4. The Discussion in its current form is not fully developed. There are several points to consider. Does SMCHD1 restrict HIV-1 and other viruses in a manner similar to HSV-1 (e.g., by binding to nucleic acid). What other cellular proteins does SMCHD1 bind to, allowing it to be an epigenetic repressor? How does SMCHD1 SUMOylation enhance its binding to HSV-1 DNA? What is the authors’ model (in schematic form) based on their data? Answers to these questions will significantly strengthen the Discussion.

5. Information provided for many references are incomplete. Please recheck the references and ensure that their details are complete.

Reviewer #2: Minor comments:

1. The authors lay summary reads almost identically to that of the abstract. I would strongly encourage the authors to re-write this lay summary to increase the readership appeal of the MS to the broad readership of PLoS Pathogens.

2. The introduction lists known HSV-1 restriction factors (e.g., MxB, TRIM22) but fails to mention other chromatin-modifiers or binding proteins (e.g., Daxx, ATRX, HIRA) known to restrict HSV-1 infection that are counteracted (degraded or displaced) by the E3 ubiquitin ligase activity of ICP0. The authors should revise their introduction and discussion accordingly to reflect the potential role of SMCHD1 to mediate chromatin silencing (Fig. S4).

3. Line 124; ‘In contrast, HSV-1 ΔICP0 replication is severely impaired in HFF and HEK293T cells, whereas HSV-1 ICP0 RFm replicates substantially more efficiently in these cell types 30, 31.’ This statement is confusing. There have been a substantial number of studies demonstrating the RING-finger (RF) to be essential for ICP0 E3 ubiquitin ligase activity and biological function to stimulate the progress of infection (e.g., PMID: 22278229). The authors should provide additional supporting data for this statement to stand for their KOS strain derived HSV-1 ICP0 RFm.

4. line 240; ‘Because ICP0 is a putative RING-type E3 ubiquitin,….’. This statement if inaccurate and should be revised. There are an abundance of studies demonstrating ICP0 to be a RING-finger E3 ubiquitin ligase that can interact with multiple host-cell factors to mediate their ubiquitination.

5. As the interaction between ICP0 and SMCHD1 occurs within the first 100aa of the N-terminus of ICP0, which predominantly includes the CK1 ICP0-T67 phosphorylation site required for RNF8 FHA domain interaction. The authors should investigate, or at least discuss, the potential role for RNF8 and/or RNF168 to mediate the (co)degradation of SMCHD1 as a component of the DDR response that utilises host SUMOylation for recruitment of host factosr to sites of DNA damage.

Reviewer #3: - The authors need to include a kinetic with the WT virus to indicate when SMCHD1 is actually degraded.

- Also, the authors need to include a kinetic with ΔICP0 both in U2OS and HFF to determine if degradation of SMCHD1 is observed later.

- The authors need to test colocalization of ICP0 with SMCHD1 at the ND10 bodies.

- In Fig. 7 authors need to provide growth curves and not a single time point for virus growth.

- In U2OS cells, HSV-1 and ΔΙCP0 generally grow to comparable levels, even though ΔΙCP0 cannot degrade SMCHD1. The authors need to provide growth curves of the 2 viruses in U2OS.

- Introduction and discussion are very similar.

**********

PLOS authors have the option to publish the peer review history of their article (what does this mean?). If published, this will include your full peer review and any attached files.

If you choose “no”, your identity will remain anonymous but your review may still be made public.

**Do you want your identity to be public for this peer review?**  For information about this choice, including consent withdrawal, please see our Privacy Policy.

Reviewer #1: No

Reviewer #2: No

Reviewer #3: No

[NOTE: If reviewer comments were submitted as an attachment file, they will be attached to this email and accessible via the submission site. Please log into your account, locate the manuscript record, and check for the action link "View Attachments". If this link does not appear, there are no attachment files.]

**Figure resubmission:**

-->While revising your submission, we strongly recommend that you use PLOS’s NAAS tool (https://ngplosjournals.pagemajik.ai/artanalysis) to test your figure files. NAAS can convert your figure files to the TIFF file type and meet basic requirements (such as print size, resolution), or provide you with a report on issues that do not meet our requirements and that NAAS cannot fix.

After uploading your figures to PLOS’s NAAS tool - https://ngplosjournals.pagemajik.ai/artanalysis, NAAS will process the files provided and display the results in the "Uploaded Files" section of the page as the processing is complete. If the uploaded figures meet our requirements (or NAAS is able to fix the files to meet our requirements), the figure will be marked as "fixed" above. If NAAS is unable to fix the files, a red "failed" label will appear above. When NAAS has confirmed that the figure files meet our requirements, please download the file via the download option, and include these NAAS processed figure files when submitting your revised manuscript.

**Reproducibility:**

To enhance the reproducibility of your results, we recommend that authors of applicable studies deposit laboratory protocols in protocols.io, where a protocol can be assigned its own identifier (DOI) such that it can be cited independently in the future. Additionally, PLOS ONE offers an option to publish peer-reviewed clinical study protocols. Read more information on sharing protocols at https://plos.org/protocols?utm_medium=editorial-email&utm_source=authorletters&utm_campaign=protocols

# Author comment: Author response to Decision Letter 1

**DOI:** 10.1371/journal.ppat.1014371.r002

21 May 2026

Attachment Submitted filename: Response to Reviewers.pdf

# Peer review: Decision Letter 1

**DOI:** 10.1371/journal.ppat.1014371.r003

**Authors:** Robert Kalejta, Anthony Nicola, Robert Kalejta, Anthony Nicola

11 Jun 2026

Dear Dr. Zhang,

We are pleased to inform you that your manuscript 'SUMOylation and ubiquitination reciprocally regulate SMCHD1 antiviral activity against herpes simplex virus 1' has been provisionally accepted for publication in PLOS Pathogens.

Before your manuscript can be formally accepted you will need to complete some formatting changes, which you will receive in a follow up email. A member of our team will be in touch with a set of requests.

Please note that your manuscript will not be scheduled for publication until you have made the required changes, so a swift response is appreciated.

IMPORTANT: The editorial review process is now complete. PLOS will only permit corrections to spelling, formatting or significant scientific errors from this point onwards. Requests for major changes, or any which affect the scientific understanding of your work, will cause delays to the publication date of your manuscript.

Should you, your institution's press office or the journal office choose to press release your paper, you will automatically be opted out of early publication. We ask that you notify us now if you or your institution is planning to press release the article. All press must be co-ordinated with PLOS.

Thank you again for supporting Open Access publishing; we are looking forward to publishing your work in PLOS Pathogens.

Best regards,

Anthony Nicola

Academic Editor

PLOS Pathogens

Robert Kalejta

Section Editor

PLOS Pathogens

Sumita Bhaduri-McIntosh

Editor-in-Chief

PLOS Pathogens

orcid.org/0000-0003-2946-9497

Michael Malim

Editor-in-Chief

PLOS Pathogens

orcid.org/0000-0002-7699-2064

***********************************************************

Reviewer Comments (if any, and for reference):

Reviewer's Responses to Questions

**Part I - Summary**

Please use this section to discuss strengths/weaknesses of study, novelty/significance, general execution and scholarship.

Reviewer #1: The following is a revised manuscript of a previous submission describing how the host antiviral factor, SMCHD1, restricts HSV-1 transcription and lytic replication. In this revised manuscript, the authors have included additional data requested by reviewers and modified the manuscript accordingly. These modifications have significantly strengthen the authors' conclusions, adding to the field's understanding of how SMCHD1 impairs the lifecycle of DNA viruses.

Reviewer #3: The authors have provided significant piece of evidence supporting that SMCHD1 restricts HSV-1 and suppresses virus replication. It appears that the sumoylated form of SMHD1 suppresses virus replication. In turn, HSV-1 ICP0 targets this restriction factor for degradation.

The study is well done and major concerns have been addressed.

**********

**Part II – Major Issues: Key Experiments Required for Acceptance**

Please use this section to detail the key new experiments or modifications of existing experiments that should be absolutely required to validate study conclusions.

Generally, there should be no more than 3 such required experiments or major modifications for a "Major Revision" recommendation. If more than 3 experiments are necessary to validate the study conclusions, then you are encouraged to recommend "Reject".

Reviewer #1: There authors have addressed major issues noted in their previous submission.

Reviewer #3: The authors have addressed the major concerns and now the manuscript is suitable for publication.

**********

**Part III – Minor Issues: Editorial and Data Presentation Modifications**

Please use this section for editorial suggestions as well as relatively minor modifications of existing data that would enhance clarity.

Reviewer #1: The authors need to check for minor typos (spelling and grammar) in their revised manuscript.

Reviewer #3: (No Response)

**********

PLOS authors have the option to publish the peer review history of their article (what does this mean?). If published, this will include your full peer review and any attached files.

If you choose “no”, your identity will remain anonymous but your review may still be made public.

**Do you want your identity to be public for this peer review?**  For information about this choice, including consent withdrawal, please see our Privacy Policy.

Reviewer #1: No

Reviewer #3: No

# Editor report: Acceptance letter

**DOI:** 10.1371/journal.ppat.1014371.r004

**Authors:** Robert Kalejta, Anthony Nicola, Robert Kalejta, Anthony Nicola

Dear Dr. Zhang,

We are delighted to inform you that your manuscript, "SUMOylation and ubiquitination reciprocally regulate SMCHD1 antiviral activity against herpes simplex virus 1," has been formally accepted for publication in PLOS Pathogens.

We have now passed your article onto the PLOS Production Department who will complete the rest of the pre-publication process. All authors will receive a confirmation email upon publication.

The corresponding author will soon be receiving a typeset proof for review, to ensure errors have not been introduced during production. Please review the PDF proof of your manuscript carefully, as this is the last chance to correct any scientific or type-setting errors. Please note that major changes, or those which affect the scientific understanding of the work, will likely cause delays to the publication date of your manuscript. Note: Proofs for Front Matter articles (Pearls, Reviews, Opinions, etc...) are generated on a different schedule and may not be made available as quickly.

Soon after your final files are uploaded, the early version of your manuscript, if you opted to have an early version of your article, will be published online. The date of the early version will be your article's publication date. The final article will be published to the same URL, and all versions of the paper will be accessible to readers.

For Research Articles, you will receive an invoice from PLOS for your publication fee after your manuscript has reached the completed accept phase. If you receive an email requesting payment before acceptance or for any other service, this may be a phishing scheme. Learn how to identify phishing emails and protect your accounts at https://explore.plos.org/phishing.

Thank you again for supporting open-access publishing; we are looking forward to publishing your work in PLOS Pathogens.

Best regards,

Sumita Bhaduri-McIntosh

Editor-in-Chief

PLOS Pathogens

orcid.org/0000-0003-2946-9497

Michael Malim

Editor-in-Chief

PLOS Pathogens

orcid.org/0000-0002-7699-2064
